# Investigations of amination reactions on an antimalarial 1,2,4-triazolo[4,3-*a*]pyrazine scaffold

**DOI:** 10.3762/bjoc.21.90

**Published:** 2025-06-10

**Authors:** Henry S T Smith, Ben Giuliani, Kanchana Wijesekera, Kah Yean Lum, Sandra Duffy, Aaron Lock, Jonathan M White, Vicky M Avery, Rohan A Davis

**Affiliations:** 1 Institute for Biomedicine and Glycomics, Griffith University, Brisbane, QLD 4111, Australia; 2 Discovery Biology, Griffith University, Brisbane, QLD 4111, Australia; 3 School of Chemistry and Bio21 Institute, The University of Melbourne, Melbourne, VIC 3010, Australia; 4 NatureBank, Griffith University, Brisbane, QLD 4111, Australia

**Keywords:** amine substitution, antimalarial, open source malaria, triazolopyrazine, 1,2,4-triazolo[4,3-*a*]pyrazines

## Abstract

1,2,4-Triazolo[4,3-*a*]pyrazines have previously been explored by the Open Source Malaria project as potent in vitro and in vivo antimalarial drug leads. With a view to generating a library of unique antimalarial 1,2,4-triazolo[4,3-*a*]pyrazines and exploring regiochemical preference for nucleophilic amines, we utilised the known synthetic 5-chloro-3-(4-chlorophenyl)-[1,2,4]triazolo[4,3-*a*]pyrazine (**1**) as a scaffold for aminations with 14 commercially available primary amines. Reacting scaffold **1** with excess primary amine at room temperature for 16 h generated the desired amine analogues in respectable yields (18–87%) and high purity (≥95%) following chromatography workup. The structures of the 14 previously undescribed amine analogues **2**–**15** were fully characterised following 1D/2D NMR, UV, and HRMS data analyses. X-ray crystallographic analysis of crystals obtained from the aminated products **2**, **7**, **10**, and **15** are also reported here. The new library of amine-substituted triazolopyrazines was screened against the *Plasmodium falciparum* 3D7 strain. The tertiary alkylamine products **10**–**14** displayed antimalarial activity with IC_50_ values ranging from 9.90 to 23.30 µM against *P. falciparum* 3D7, with compounds **10**–**12** demonstrating no toxicity at 80 µM against the human embryonic kidney cell line HEK293.

## Introduction

Malarial disease is a potentially fatal, acute febrile illness caused by infection with any of several species of vector-borne apicomplexan parasites in the genus *Plasmodium* [1]. The African endemic parasite, *P. falciparum* and the more broadly distributed species *P. vivax* are responsible for the preponderance of new cases and deaths globally [1]. The World Health Organization (WHO) estimated that in 2023, there were 263 million cases of malarial disease and 597,000 deaths caused by infection with *Plasmodium* parasites and recommends artemisinin combination therapy (ACT) for the treatment of both uncomplicated and severe malaria as a frontline treatment [1]. ACT includes the use of an artemisinin agent in concert with a partner drug (most often a quinoline drug), which possesses a different mechanism of action and a longer half-life (to improve total cure rates and forestall the development of drug resistance) [2]. Unfortunately, partial artemisinin resistance has been documented in Southeast Asia for more than a decade, and parasites with partial artemisinin resistance have been detected in patient isolates in African geographies [1–2]. *P. falciparum* isolates have also been detected in various global regions with at least some measure of resistance to all frontline ACT partner drugs [1–2]. New chemotherapeutics are urgently needed to manage malarial disease and forestall or circumvent ACT resistant parasites [1].

Open Source Malaria (OSM) is a drug development and medicinal chemistry platform established by Matthew Todd formerly of The University of Sydney (currently at University College London) with funding from the product development partnership platform, Medicines for Malaria Venture (MMV) [3]. OSM’s aim is to “find a drug for malaria as quickly as possible” [3]. In the past, OSM synthesised and screened various derivatives of three related parent scaffolds (series 1–3) provided by GlaxoSmithKline (GSK) in non-commercial antimalarial screening datasets [4]. The group’s current primary research focus surrounds a fourth series of antimalarial 1,2,4-triazolo[4,3-*a*]pyrazine analogues [5] identified from screening a Pfizer compound library, which was conducted by Discovery Biology (Griffith University). The OSM series 4 database contains hundreds of unique 1,2,4-triazolo[4,3-*a*]pyrazine (hereafter referred to as “triazolopyrazine”) analogues, including derivatives inherited from Pfizer and newly synthesised analogues [5]. Triazolopyrazine compounds of OSM series 4 are believed to dysregulate *P. falciparum* ATP4ase (*Pf*ATP4), possibly as a direct inhibitor [5]. Series 4 compounds have demonstrated in vitro potency against *P. falciparum* with IC_50_ values in the micromolar and low nanomolar range and appear to have little polypharmacology or cytotoxicity [6–8]. Some members of series 4 have also proven effective for the rapid clearance of the *P. falciparum* 3D7 strain in mice engrafted with human erythrocytes, after oral administration of the compounds [5,9].

**Figure 1:**
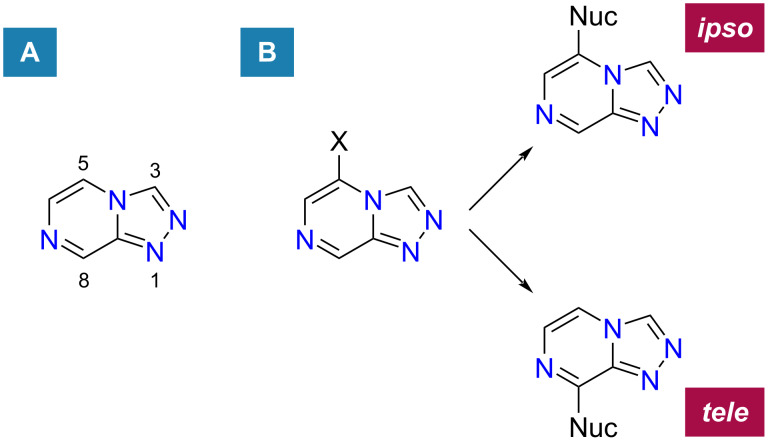
(A) Position numbering on the pyrazine ring of 1,2,4-triazolo[4,3-*a*]pyrazine. (B) Illustration of *ipso*- and *tele*-substitution products for reactions of nucleophiles with 5-halogenated 1,2,4-triazolo[4,3-*a*]pyrazine, where X = halogen and Nuc = nucleophile.

Triazolopyrazines halogenated on the pyrazine ring represent synthetically useful targets for elaboration of this active scaffold, and the regioselectivity of amine nucleophiles (amongst others) has been explored by Korsik et al. for 5-position halogenated scaffolds [10]. Several different triazolopyrazine compounds bearing chlorine, bromine or iodine at the 5-position, gave no *ipso*-substituted (5-substituted) products when refluxed in toluene with phenethylamine, with all major products being substituted at the 8-position (*tele*-substituted; Figure 1), distant from the halogen leaving group at position 5 [10]. A plausible reaction mechanism was proposed by Korsik et al. [10]. The antimalarial activity of series 4 triazolopyrazine scaffolds is generally reduced by substitution of an amine functionality at the 8-position of the pyrazine ring [10–11].

During small-scale late-stage functionalisation of natural products, we have observed that amination or amidation with primary amines often involves less of a kinetic barrier than might otherwise be expected [12–14]. The use of readily available liquid amines in excess can, sometimes, ostensibly compensate for low rates of conversion to aminated or amidated products, without the need for high temperatures [12–14]. We set out to ascertain whether aminated triazolopyrazines can be obtained from 5-chloro-3-(4-chlorophenyl)-[1,2,4]triazolo[4,3-*a*]pyrazine in good yield with this straightforward methodology (and without the need for toluene reflux), and to determine the distribution of products obtained for this synthetic approach, by exhaustive analysis of reaction mixtures by HPLC, MS and NMR. Ultimately, 14 aminated derivatives of 5-chloro-3-(4-chlorophenyl)-[1,2,4]triazolo[4,3-*a*]pyrazine were synthesised, purified (≥95% purity, determined by ^1^H and ^13^C NMR and HPLC–MS analysis) and assayed for in vitro antimalarial activity.

## Results and Discussion

### Synthesis of aminated triazolopyrazine analogues

**Scheme 1:**
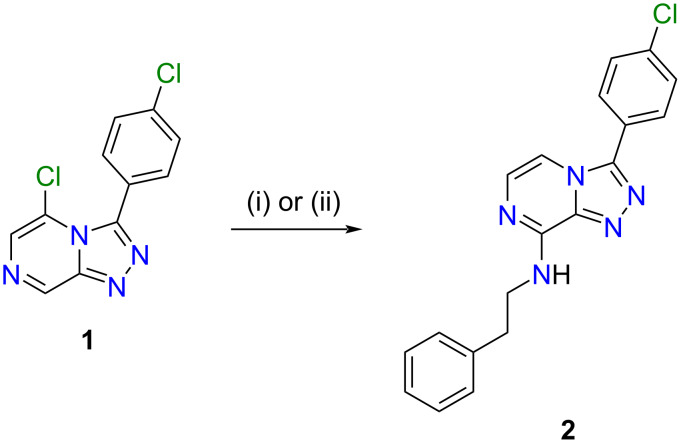
Treatment of **1** with phenethylamine (PEA) under two different reaction conditions, (i) or (ii), gave **2** in 70% and 82% yield, respectively. Reagents and conditions: (i) 3 equiv PEA, PhCH_3_, silica, rt, 6 h; (ii) 10 equiv PEA, rt, 6 h.

The amination of 5-chloro-3-(4-chlorophenyl)-[1,2,4]triazolo[4,3-*a*]pyrazine (compound **1**; Scheme 1) according to the approach previously described by Korsik et al. [10] was performed, but without reflux, in order to compare the yield obtained for the aminated product under our further modified conditions (neat phenethylamine only, room temperature; Scheme 1). Reactions were set up in tandem and samples from both reaction vessels were taken, at 2, 4 and 6 h time points and used for TLC analysis to monitor reaction progress. The reactions proceeded in parallel, and both reactions had reached completion at 6 h. After isolation in high purity (≥95%) by silica flash column chromatography (and additionally by HPLC for the toluene reaction), the reactions also gave comparable yields of compound **2** (70% for toluene/silica and 82% for only phenethylamine at room temperature). Only the *tele*-substituted product was observed in either reaction mixture, consistent with previous reports [10].

**Figure 2:**
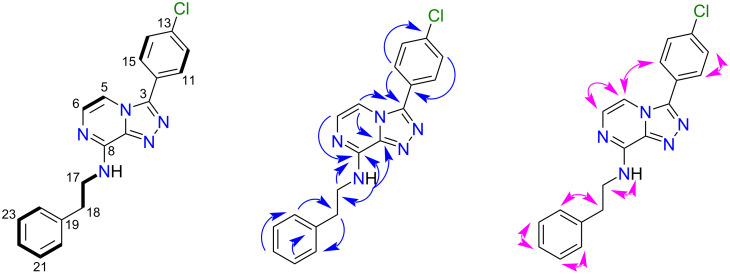
Key COSY (**–**), HMBC (→) and ROESY (↔) correlations for compound **2**.

**Figure 3:**
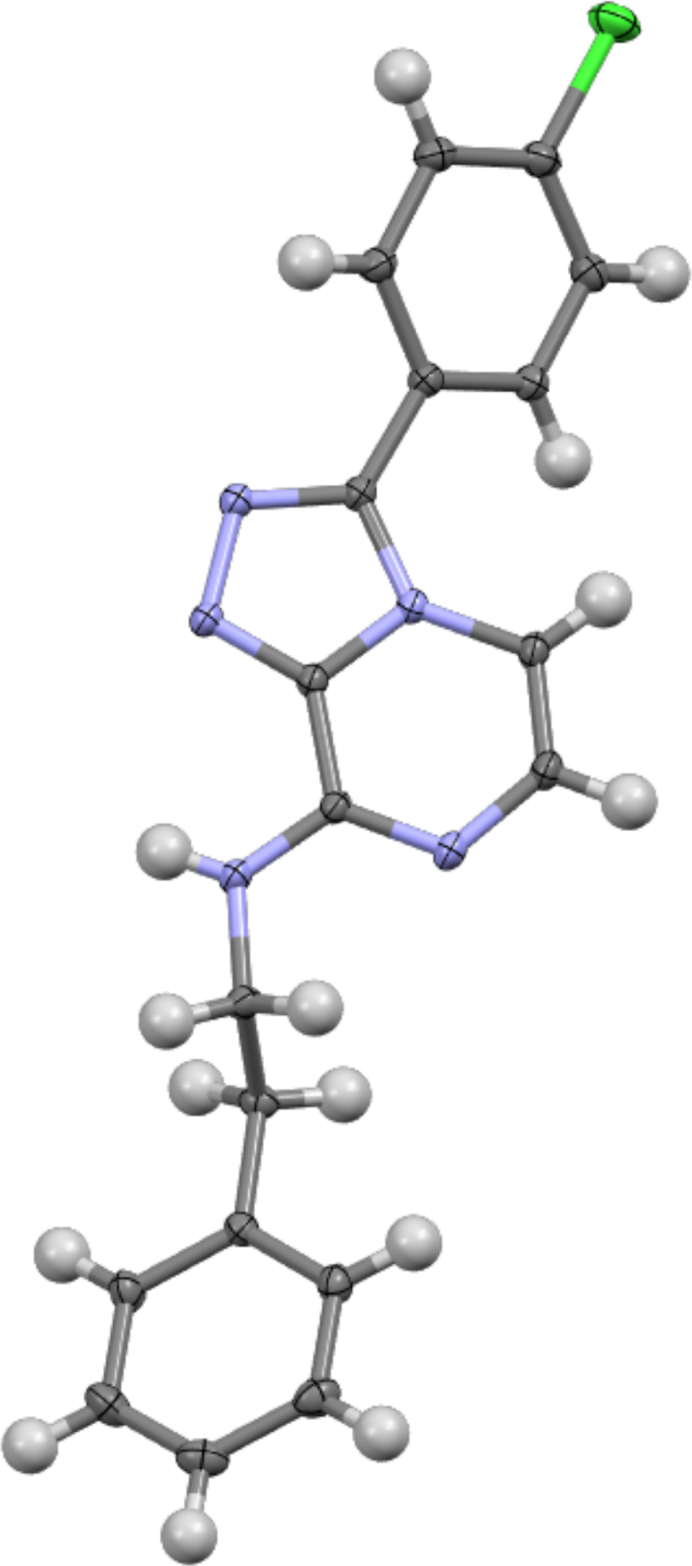
Thermal ellipsoid plot of compound **2**.

A single and identical product was obtained from each reaction and the product, **2**, was characterised following analysis of HRESIMS and 1D (^1^H, ^13^C) and 2D (COSY, HSQC, HMBC, ROESY) NMR data. The HRESIMS data of **2** showed a Na adduct ion at *m*/*z* 372.0991 [M + Na]^+^ (calcd for C_19_H_16_^35^ClN_5_Na, 372.0991), that enabled a molecular formula of C_19_H_16_ClN_5_ to be assigned to **2**. The ^1^H NMR spectrum of **2** displayed two aromatic doublets corresponding to nuclei on the triazolopyrazine scaffold [δ_H_ 7.76 (H-5), 7.38 (H-6)], and displayed two resonances [δ_H_ 7.93 (H-11, H-15), 7.69 (H-12, H-14)] corresponding to the four protons on the 4-chlorophenyl substituent of the starting material. Substitution with phenethylamine was confirmed by the presence of an ethylamino pendant chain, showing an exchangeable amine proton triplet at δ_H_ 8.30 (H-16, *J* = 5.8 Hz), which was COSY coupled to an ethyl spin system [δ_H_ 3.74 (H-17, *J* = 5.8, 7.3 Hz), δ_H_ 2.98 (H-18, *J* = 7.3 Hz)]. Three aromatic resonances were also observed, which corresponded to the phenyl moiety [(δ_H_ 7.30 (H-21, H-23), 7.28 (H-20, H-24), 7.20 (H-22)], which we have previously characterised for similar triazolopyrazines [12]. The ^13^C NMR, HSQC and HMBC spectrum of **2** allowed unambiguous assignment of the amine substitution at C-8 with the amine proton (δ_H_ 8.30) showing strong HMBC correlations into the triazolopyrazine scaffold [δ_C_ 147.9 (C-8), 139.7 (C-9)]. 2D NMR correlations used to characterise the structure of **2** are shown in Figure 2, along with relevant position numbering. Crystals obtained for **2** were analysed by X-ray crystallographic studies and confirmed the NMR-based structure assignment. The crystal structure of **2** is shown as a thermal ellipsoid plot below (Figure 3).

**Scheme 2:**
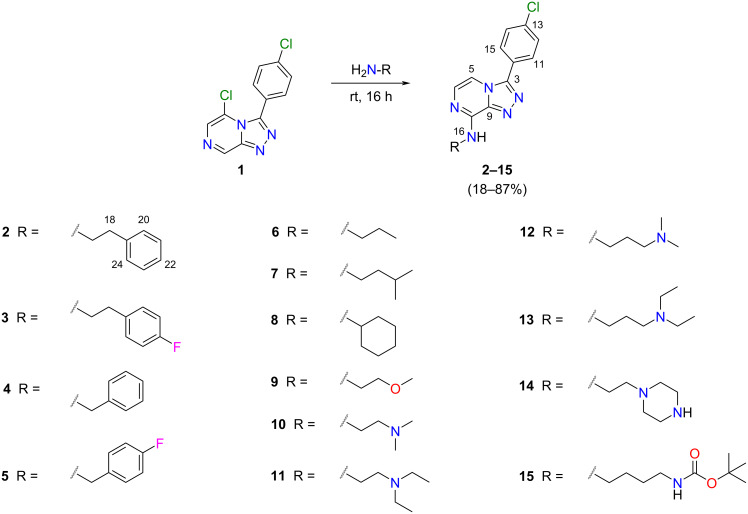
Chemical structures, reagents and conditions used to synthesise the new aminated triazolopyrazines **2**–**15**.

**Figure 4:**
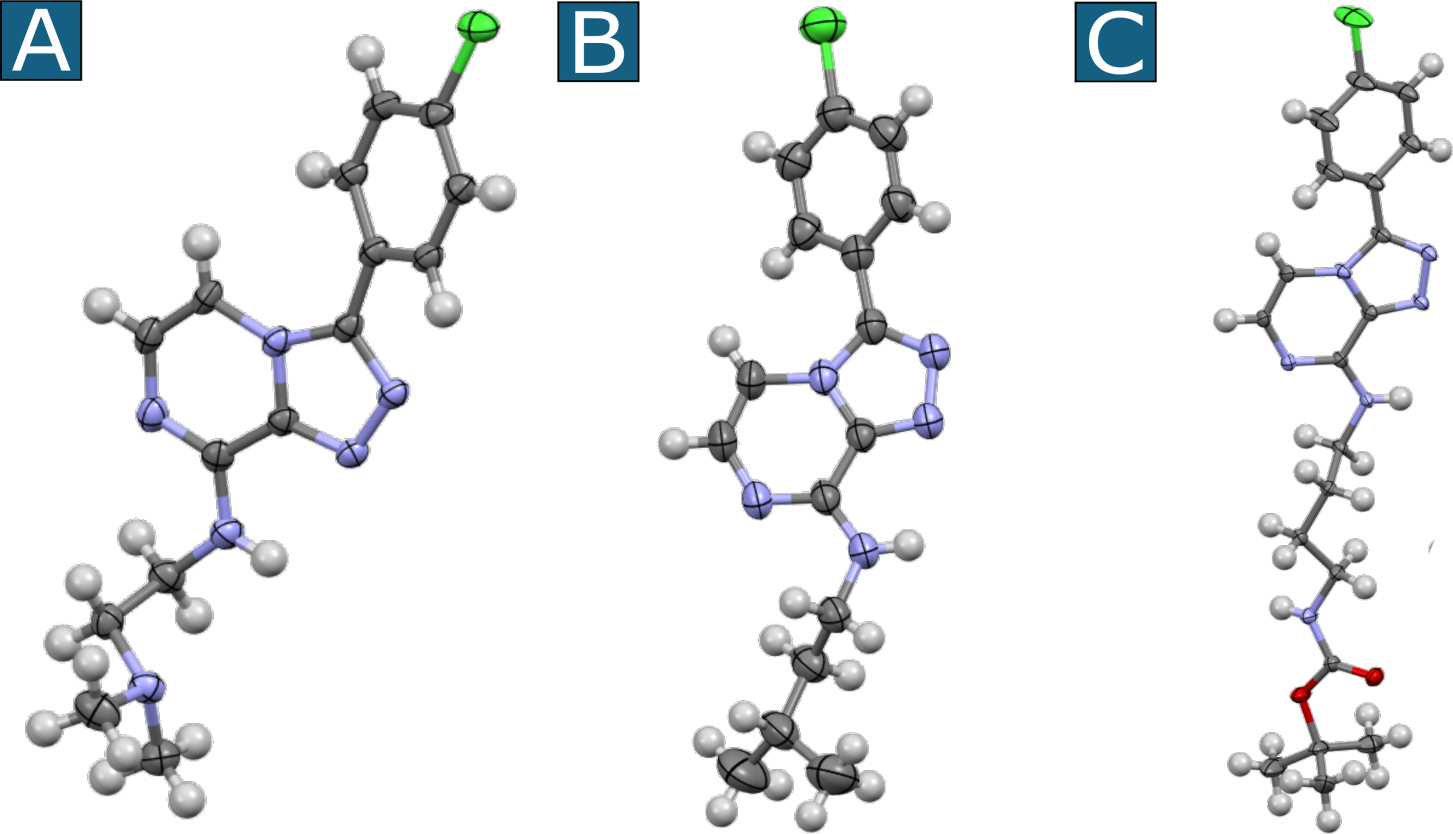
Thermal ellipsoid plots for compounds **7** (A), **10** (B) and **15** (C).

With a simplified approach for the synthesis of aminated triazolopyrazines from 5-halogen substituted triazolopyrazines and primary amines now in hand, 13 other commercially available liquid primary amines were employed for the synthesis of additional aminated triazolopyrazine analogues (Scheme 2). TLC analysis of the room temperature reaction with phenethylamine (as previously discussed), and later with propylamine, indicated that the reaction had reached completion at 6 h. However, to account for the possibility of slower rates of conversion for other primary amine reagents, the reactions for **3**–**15** were allowed to proceed at room temperature for 16 h. Crude reaction mixtures were separated by silica flash column chromatography, and in some cases, were subsequently separated by HPLC where required. All reactions gave easily isolated product in high purity (≥95%), with yields ranging from 18% to 87%. No *ipso*-substituted products, and no triazole–imidazole rearrangement products [10] were detected by ^1^H NMR in any UV-active fractions following chromatographic separations. To the best of our knowledge, none of these triazolopyrazine compounds (**2**–**15**) have yet been reported [15]. Compounds **3**–**15** all had their chemical structures confirmed following detailed analysis of 1D/2D NMR and HRESIMS data. Further X-ray crystallographic studies were undertaken on crystalline material of compounds **7**, **10**, and **15**. The thermal ellipsoid plots for these compounds are shown in Figure 4.

It is notable that piperidine, both at room temperature and under reflux, has been reported to give the *ipso*-product when reacted with 5-chloro-[1,2,4]triazolo[4,3-*a*]pyrazine, albeit as a minor product [10], but none of the 14 primary amines explored in this work gave *ipso*-products. This suggests that the 8-substituted *tele-*product is the thermodynamic (preferred) product for treatment of 5-halogenated triazolopyrazines with amine nucleophiles, and the only likely product from reaction with primary amines at room temperature.

### Biological studies

All compounds (**1**–**15**) were assayed for antimalarial activity against the chloroquine sensitive *P. falciparum* 3D7 strain, and a preliminary assessment of compound selectivity was made using the human embryonic kidney cell line HEK293, according to previously reported protocols [16–17]. The results are shown in Table 1, including IC_50_ values for the reference compounds puromycin, chloroquine, pyrimethamine, and dihydroartemisinin.

**Table 1:** Biological data for activity of triazolopyrazine analogues **1**–**15** against *P. falciparum* 3D7 and the non-cancerous cell line HEK293.

Compound	*Pf* 3D7^a^ IC_50_ ± SD [µM]	HEK293 % inhibition ± SD^b^	Selectivity index^c^

**1**	12.62 ± 1.90	–	>6
**2**	–	–	–
**3**	–	74 ± 7	–
**4**	–	–	–
**5**	–	–	–
**6**	–	–	–
**7**	–	–	–
**8**	–	–	–
**9**	–	–	–
**10**	23.30 ± 1.70	–	>3
**11**	22.0 ± 2.40	–	>4
**12**	22.0 ± 1.56	–	>4
**13**	11.30 ± 0.57	48 ± 5	–
**14**	9.90 ± 1.27	84 ± 4	–
**15**	–	57 ± 4	–

Reference compounds	*Pf* 3D7^a^ IC_50_ ± SD [nM]	HEK293 IC_50_ ± SD [nM]	Selectivity index^c^

PMY	33.80 ± 5.66	430.0 ± 28.30	13
CQ	21.2 ± 5.66	–	–
PMA	11.2 ± 2.12	–	–
DHA	0.475 ± 0.09	–	–

^a^*Pf* 3D7 = *P. falciparum* 3D7 (chloroquine-sensitive strain). ^b^The mean % inhibition of the compounds (duplicate, *n* = 1) against HEK293 ± SD (%). ^c^Selectivity index (fold difference) with respect to compound activity against HEK293, i.e., IC_50_^(HEK293)^/IC_50_^(^*^Pf^*^3D7)^*.* SD = standard deviation, PMY = puromycin, CQ = chloroquine, PMA = pyrimethamine, DHA = dihydroartemisinin.

Compounds **2**–**9** and **15** did not reach 100% growth inhibition at the assay top dose (80 µM), against *Pf* 3D7. Thus, determination of accurate IC_50_ values was not possible. The mean IC_50_ values (duplicate, *n* = 1) for compounds **10**–**14** against *Pf* 3D7 were calculated and are shown in Table 1. The antimalarial activity of **10**–**14** was comparable to **1**, which is unusual for 8-substituted triazolopyrazines [6,8,11]. It is notable that compounds **10**–**14** are all tertiary alkylamines, and these were the only 8-aminated products to display antimalarial activity. Unlike some OSM compounds for which SAR data is available, the starting material **1** has not itself been assayed for anti-*Pf*ATP4 activity [9]. The data presented here for **10**–**14** strongly suggest the possibility that tertiary alkylamine substitution at position 8 may be tolerated in regard to anti-*Pf*ATP4 activity. However, data involving triazolopyrazine scaffolds with confirmed anti-*Pf*ATP4 activity would be required to definitively assess the tolerability of tertiary alkylamines at position 8.

Whilst compounds **1**, **2** and **4**–**12** showed no cytotoxicity against HEK293 cells at the top concentration tested (80 µM), for those compounds with concomitant antimalarial activity (**1**, **10**–**12**), their limited activity against *Pf* 3D7 resulted in low selectivity indices, as indicated in Table 1. Compounds **3** and **13**–**15** demonstrated some inhibition of HEK293 at the highest concentrated tested, but did not display sigmoidal dose-dependent kinetics, thus accurate IC_50_ values were unable to be determined. Meaningful selectivity indices for these compounds could, therefore, likewise not be calculated. Hence, the mean % growth inhibition (duplicate, *n* = 1) at the top concentration is indicated in Table 1.

## Conclusion

A modified approach for amine substitution of 5-chloro-3-(4-chlorophenyl)-[1,2,4]triazolo[4,3-*a*]pyrazine was explored here for phenethylamine and 13 other liquid primary amines. This convenient synthesis involving only addition of liquid amine to starting material, and stirring at room temperature, was found to proceed readily within 6 to 16 h, in generally high yield. Exclusive *tele*-substitution (8-substitution) occurred in all reactions carried out. All of the 8-substituted products synthesised for these studies have added to the Davis Open Access Compound Library which is curated by Compounds Australia (Griffith University) and made available to biomedical researchers [18–19]. Tertiary alkylamines appeared to be unusually well tolerated in regard to antimalarial activity when substituted at the 8-position on **1**. It is potentially possible, but not conclusive, that similar substitution would be tolerated in other [1,2,4]triazolo[4,3-*a*]pyrazine leads with known anti-*Pf*ATP4 activity.

## Experimental

### General experimental

Melting points were measured using a Cole-Parmer (Chicago, IL, USA) melting point apparatus and were uncorrected. UV spectra were recorded using an Ocean Optics (USB-ISS-UV/VIS) spectrophotometer. NMR spectra were recorded at 25 °C on a Bruker (Billerica, MA, USA) AVANCE III™ HD 500 MHz NMR spectrometer equipped with a cryoprobe. MestreNova™ version 14.3.3 software was used for NMR data analysis. The ^1^H and ^13^C NMR chemical shifts were referenced to solvent peaks for (CD_3_)_2_SO (δ_H_ 2.50, δ_C_ 39.52). LRESIMS data was recorded on a Thermo Scientific (Waltham, MA, USA) UltiMate™ 3000 RS UHPLC coupled to a Thermo Scientific ISQ™ EC single quadruple ESI mass spectrometer. HRESIMS data were acquired on a Bruker maXis II ETD ESI-qTOF. TLC was carried out on Merck (Kenilworth, NJ, USA) silica gel 60 F254 pre-coated aluminium plates and developed plates were visualised using UV light at 254 and 365 nm.

Reaction mixtures were pre-adsorbed onto Merck silica gel (40–63 µm, 143 Å) and Isolute™ silica (30 × 40 mm, 10 g, 55 µm, 54 Å) SPE cartridges were used for normal-phase, small-scale separations. Alltech (Lexington, KY, USA) Davisil™ C_18_-bonded silica (35–70 µm, 60 Å) were used for pre-adsorption work before reversed-phase HPLC separations. Pre-adsorbed material was packed into an Alltech stainless steel guard cartridge (10 × 30 mm) then attached to an HPLC column prior to fractionation. Thermo Electron Betasil™ C_18_-bonded silica (5 μm, 100 Å, 150 × 21.2 mm) columns were used for reversed-phase HPLC separations. A Thermo Scientific Dionex Ultimate™ 3000 UHPLC was used for HPLC separations. All solvents used for chromatography and MS were Honeywell Burdick & Jackson (Muskegon, MI, USA) or RCI Labscan (Bangkok, Thailand) HPLC grade. H_2_O was filtered using a Sartorius (Göttingen, Lower Saxony, Germany) Arium™ Pro VF ultrapure water system. Synthetic reagents were purchased from Sigma-Aldrich (St. Louis, MO, USA) and used without further purification. The starting material, 5-chloro-3-(4-chlorophenyl)-[1,2,4]triazolo[4,3-*a*]pyrazine (**1**), was previously synthesised and purified by the Davis group [8].

### In vitro antiplasmodial image-based assay

*Plasmodium falciparum* 3D7 were cultured in RPMI1640 (Life Technologies, Camarillo, CA, USA) supplemented with 2.5 mg/mL Albumax II, 5% AB human serum, 25 mM HEPES, and 0.37 mM hypoxanthine. Human red blood cells (RBC) (O+) were supplied by Australian Red Cross LifeBlood in accordance with agreement 23-05QLD-23. Use of human RBC for antiplasmodial experimentation was in accordance with Griffith University Human Ethics Exemption Approval #03/08/11019. Ring-stage parasites were treated with compounds following two rounds of sorbitol synchronisation, as previously described [16]. Puromycin, chloroquine, pyrimethamine, and dihydroartemisinin were used as reference compounds. Following incubation of assay plates for 72 h at 37 °C, and 5% CO_2_ and 5% O_2_, parasites were stained with 2-(4-amidinophenyl)-1*H*-indole-6-carboxamidine (DAPI) and imaged using an Opera PhenixPlus™ High Content Screening System (PerkinElmer, Waltham, MA, USA). Images were analysed using the Harmony software (PerkinElmer, Waltham, MA, USA).

### In vitro cytotoxicity assay

Human embryonic kidney (HEK293) cells were maintained in DMEM (Life Technologies, Camarillo, CA, USA) containing 10% FBS (Hyclone™ ThermoFisher, Melbourne, Australia). Cytotoxicity testing was undertaken as previously described [17]. In brief, 5 μL of test compound was added to the well of black/clear tissue culture-treated, 384-well plates containing 2000 adherent HEK293 cells/well and incubated 72 h at 37 °C in 5% CO_2_. At 6 h, 5 µL of 600 µM resazurin, diluted in growth media, was added. Plates were further incubated for 6 h and measured for fluorescence at 530 nm excitation and 595 nm emission. The % inhibition was calculated using 0.4% DMSO (no inhibition) and 50 μM puromycin (100% inhibition) data. Puromycin was included as a reference compound to assess assay validity. IC_50_ values were obtained by plotting % inhibition against log dose using GraphPad Prism v.6 (San Diego, CA, USA) nonlinear regression with a variable slope plot.

### Modified synthesis of 3-(4-chlorophenyl)-*N*-phenethyl-[1,2,4]triazolo[4,3-*a*]pyrazin-8-amine (**2**)

5-Chloro-3-(4-chlorophenyl)-[1,2,4]triazolo[4,3-*a*]pyrazine (106 mg, 0.4 mmol), was dissolved in phenethylamine (500 µL, 1.6 mmol, 10 equiv) and the reaction mixture stirred at room temperature for 16 h, then pre-adsorbed to silica (≈1 g) overnight. Purification was performed as described in Supporting Information File 1. Pure fractions (≥95%) obtained from reversed-phase HPLC separation were then combined to obtain the desired product, 3-(4-chlorophenyl)-*N*-phenethyl-[1,2,4]triazolo[4,3-*a*]pyrazin-8-amine (**2**) as yellow needles (115 mg, 82%).

### General method for the synthesis of the aminated triazolopyrazine library (**3**–**15**)

5-Chloro-3-(4-chlorophenyl)-[1,2,4]triazolo[4,3-*a*]pyrazine (**1**, 53 mg, 0.20 mmol) was dissolved in excess liquid amine (500 µL) and the mixture was stirred at room temperature for 16 h. Purification was performed as described in Supporting Information File 1, and relevant fractions containing high purity product (≥95%) were combined.

**3-(4-Chlorophenyl)-*N*-phenethyl-[1,2,4]triazolo[4,3-*a*]pyrazin-8-amine (2).** Yellow needles (115 mg, 82%); mp 224–226 °C; UV (MeOH) λ_max_, nm (log ε): 250 (4.09), 293 (3.83); ^1^H NMR (500 MHz, (CD_3_)_2_SO) δ_H_ 8.30 (t, *J* = 5.8 Hz, 1H, H-16), 7.93 (m, 2H, H-11, H-15), 7.76 (d, *J* = 4.8 Hz, 1H, H-5), 7.69 (m, 2H, H-12, H-14), 7.38 (d, *J* = 4.8 Hz, 1H, H-6), 7.30 (m, 2H, H-21, H-23), 7.28 (m, 2H, H-20, H-24), 7.20 (tt, *J* = 6.8, 2.0 Hz, 1H, H-22), 3.74 (dt, *J* = 5.8, 7.3 Hz, 2H, H-17), 2.98 (t, *J* = 7.3 Hz, 2H, H-18); ^13^C NMR (125 MHz, (CD_3_)_2_SO) δ_C_ 147.9 (C-8), 146.8 (C-3), 139.7 (C-9), 139.5 (C-19), 135.0 (C-13), 130.3 (C-6), 129.8 (2C, C-11, C-15), 129.4 (2C, C-12, C-14), 128.7 (2C, C-20, C-24), 128.3 (2C, C-21, C-23), 126.1 (C-22), 125.1 (C-10), 106.0 (C-5), 41.6 (C-17), 34.5 (C-18); LRMS (ESI-SQ) *m*/*z*: 350 [M + H]^+^_,_ 372 [M + Na]^+^; HRESIMS–qTOF (*m*/*z*): [M + Na]^+^ calcd for C_19_H_16_ClN_5_Na, 372.0991; found, 372.0991.

**3-(4-Chlorophenyl)-*N*-isopentyl-[1,2,4]triazolo[4,3-*a*]pyrazin-8-amine (7).** White needles (56 mg, 89%); mp 193–195 °C; UV (MeOH) λ_max_, nm (log ε): 250 (4.33), 297 (4.05); ^1^H NMR (500 MHz, (CD_3_)_2_SO) δ_H_ 8.22 (brt, *J* = 5.7 Hz, 1H, H-16), 7.93 (m, 2H, H-11, H-15), 7.73 (d, *J* = 4.8 Hz, 1H, H-5), 7.69 (m, 2H, H-12, H-14), 7.35 (d, *J* = 4.8 Hz, 1H, H-6), 3.52 (m, 2H, H-17), 1.65 (m, 1H, H-19), 1.54 (m, 2H, H-18), 0.92 (d, *J* = 6.6 Hz, 6H, H-20, H-21); ^13^C NMR (125 MHz, (CD_3_)_2_SO) δ_C_ 148.0 (C-8), 146.8 (C-3), 139.7 (C-9), 134.9 (C-13), 130.3 (C-6), 129.8 (2C, C-11, C-15), 129.3 (2C, C-12, C-14), 125.2 (C-10), 105.7 (C-5), 38.4 (C-17), 37.5 (C-18), 25.4 (C-19), 22.5 (2C, C-20, C-21); LRMS (ESI-SQ) *m*/*z*: 316 [M + H]^+^; HRESIMS–qTOF (*m*/*z*): [M + H]^+^ calcd for C_16_H_19_^35^ClN_5_, 316.1329; found, 316.1323; [M + Na]^+^ calcd for C_16_H_18_^35^ClN_5_Na, 338.1143; found, 338.1142; [2M + H]^+^ calcd for C_32_H_37_^35^Cl_2_N_10_, 631.2580; found, 631.2572.

***N*^1^-(3-(4-Chlorophenyl)-[1,2,4]triazolo[4,3-*a*]pyrazin-8-yl)-*N*^2^,*N*^2^-dimethylethane-1,2-diamine (10).** Pale yellow needles (43 mg, 68%); mp 153–155 °C; UV (MeOH) λ_max_, nm (log ε): 253 (4.36), 294 (4.08); ^1^H NMR (500 MHz, (CD_3_)_2_SO) δ_H_ 7.95 (t, *J* = 5.5 Hz, 1H, H-16), 7.91 (m, 2H, H-11, H-15), 7.74 (d, *J* = 4.8 Hz, 1H, H-5), 7.67 (m, 2H, H-12, H-14), 7.34 (d, *J* = 4.8 Hz, 1H, H-6), 3.60 (dt, *J* = 5.5, 6.6 Hz, 2H, H-17), 2.55 (t, *J* = 6.6 Hz, 2H, H-18), 2.22 (s, 6H, H-20, H-21); ^13^C NMR (125 MHz, (CD_3_)_2_SO) δ_C_ 148.0 (C-8), 146.8 (C-3), 139.7 (C-9), 135.0 (C-13), 130.3 (C-6), 129.8 (2C, C-11, C-15), 129.4 (2C, C-12, C-14), 125.1 (C-10), 106.0 (C-5), 57.4 (C-18), 45.1 (2C, C-20, C-21), 37.9 (C-17); LRMS (ESI-SQ) *m*/*z*: 317 [M + H]^+^; HRESIMS–qTOF (*m*/*z*): [M + H]^+^ calcd for C_15_H_18_^35^ClN_6_, 317.1276; found, 317.1279.

***tert*-Butyl(4-((3-(4-chlorophenyl)-[1,2,4]triazolo[4,3-*a*]pyrazin-8 yl)amino)butyl)carbamate (15).** Yellow needles (47 mg, 57%); mp 154–156 °C; UV (MeOH) λ_max_, nm (log ε): 250 (4.71), 295 (4.33); ^1^H NMR (500 MHz, (CD_3_)_2_SO) δ_H_ 8.24 (brt, *J* = 5.7 Hz, 1H, H-16), 7.90 (m, 2H, H-11, H-15), 7.71 (d, *J* = 4.8 Hz, 1H, H-5), 7.66 (m, 2H, H-12, H-14), 7.33 (d, *J* = 4.8 Hz, 1H, H-6), 6.79 (t, *J* = 5.6 Hz, 1H, H-21), 3.48 (m, 2H, H-17), 2.94 (m, 2H, H-20), 1.61 (m, 2H, H-18), 1.44 (m, 2H, H-19), 1.35 (s, 9H, H-25, H-26, H-27); ^13^C NMR (125 MHz, (CD_3_)_2_SO) δ_C_ 155.7 (C-22), 148.1 (C-8), 146.8 (C-3), 139.8 (C-9), 135.0 (C-13), 130.4 (C-6), 129.8 (2C, C-11, C-15), 129.4 (2C, C-12, C-14), 125.2 (C-10), 105.8 (C-5), 77.4 (C-24), 39.8 (C-17), 39.4 (C-20), 28.3 (3C, C-25, C-26, C-27), 27.2 (C-19), 26.0 (C-18). LRESIMS (SQ) *m*/*z*: 417 [M + H]^+^; HRESIMS–qTOF (*m*/*z*): [M + H]^+^ calcd for C_20_H_26_^35^ClN_6_O_2_, 417.1800; found, 417.1798; [M + Na]^+^ calcd for C_20_H_26_^35^ClN_6_NaO_2_, 439.1620; found, 439.1616.

## Supporting Information

File 1 Complete experimental methods, crystallographic data for **2**, **7**, **10** and **15**, characterisation data and 1D/2D NMR spectra (^1^H, ^13^C, COSY, HSQC and HMBC) for **2**–**15**.

## Data Availability

Data generated and analyzed during this study is available from the corresponding author upon reasonable request.
