# Supplementary material for: Investigations of amination reactions on an antimalarial 1,2,4-triazolo[4,3-a]pyrazine scaffold
Source: Beilstein J Org Chem. 2025 Jun 10;21:1126–34. doi: 10.3762/bjoc.21.90 (PMC12207250; doi:10.3762/bjoc.21.90)

BEILSTEIN JOURNAL OF ORGANIC CHEMISTRY

Supporting Information

for

Investigations of amination reactions on an antimalarial 1,2,4-triazolo[4,3-*a*]pyrazine scaffold

Henry S. T. Smith, Ben Giuliani, Kanchana Wijesekera, Kah Yean Lum, Sandra Duffy, Aaron Lock, Jonathan M. White, Vicky M. Avery and Rohan A. Davis

Beilstein J. Org. Chem. **2025,** *21,* 1126–1134. doi:10.3762/bjoc.21.90

Complete experimental methods, crystallographic data for 2, 7, 10 and 15, characterisation data and 1D/2D NMR spectra (^{1}H, ^{13}C, COSY, HSQC and HMBC) for 2–15

License and Terms: This is a supporting information file under the terms of the Creative Commons Attribution License (https://creativecommons.org/licenses/by/4.0). Please note that the reuse, redistribution and reproduction in particular requires that the author(s) and source are credited and that individual graphics may be subject to special legal provisions.
The license is subject to the *Beilstein Journal of Organic Chemistry* terms and conditions: (https://www.beilstein-journals.org/bjoc/terms)

Table of contents

S1: Experimental methods S1
S2: Supporting information references S14
S3: NMR data table for compound **2** S15
S4: ^{1}H NMR spectrum of compound **2** in $(CD_3)_2SO$ S16
S5: ^{13}C NMR spectrum of compound **2** in $(CD_3)_2SO$ S17
S6: NMR data table for compound **3** S18
S7: ^{1}H NMR spectrum of compound **3** in $(CD_3)_2SO$ S19
S8: ^{13}C NMR spectrum of compound **3** in $(CD_3)_2SO$ S20
S9: NMR data table for compound **4** S21
S10: ^{1}H NMR spectrum of compound **4** in $(CD_3)_2SO$ S22
S11: ^{13}C NMR spectrum of compound **4** in $(CD_3)_2SO$ S23
S12: NMR data table for compound **5** S24
S13: ^{1}H NMR spectrum of compound **5** in $(CD_3)_2SO$ S25
S14: ^{13}C NMR spectrum of compound **5** in $(CD_3)_2SO$ S26
S15: NMR data table for compound **6** S27
S16: ^{1}H NMR spectrum of compound **6** in $(CD_3)_2SO$ S28
S17: ^{13}C NMR spectrum of compound **6** in $(CD_3)_2SO$ S29
S18: NMR data table for compound **7** S30
S19: ^{1}H NMR spectrum of compound **7** in $(CD_3)_2SO$ S31
S20: ^{13}C NMR spectrum of compound **7** in $(CD_3)_2SO$ S32
S21: NMR data table for compound **8** S33
S22: ^{1}H NMR spectrum of compound **8** in $(CD_3)_2SO$ S34
S23: ^{13}C NMR spectrum of compound **8** in $(CD_3)_2SO$ S35
S24: NMR data table for compound **9** S36
S25: ^{1}H NMR spectrum of compound **9** in $(CD_3)_2SO$ S37
S26: ^{13}C NMR spectrum of compound **9** in $(CD_3)_2SO$ S38
S27: NMR data table for compound **10** S39
S28: ^{1}H NMR spectrum of compound **10** in $(CD_3)_2SO$ S40
S29: ^{13}C NMR spectrum of compound **10** in $(CD_3)_2SO$ S41
S30: NMR data table for compound **11** S42
S31: ^{1}H NMR spectrum of compound **11** in $(CD_3)_2SO$ S43
S32: ^{13}C NMR spectrum of compound **11** in $(CD_3)_2SO$ S44
S33: NMR data table for compound **12** S45
S34: ^{1}H NMR spectrum of compound **12** in $(CD_3)_2SO$ S46
S35: ^{13}C NMR spectrum of compound **12** in $(CD_3)_2SO$ S47

S36: NMR data table for compound **13** S48
S37: ^{1}H NMR spectrum of compound **13** in $(CD_3)_2SO$ S49
S38: ^{13}C NMR spectrum of compound **13** in $(CD_3)_2SO$ S50
S39: NMR data table for compound **14** S51
S40: ^{1}H NMR spectrum of compound **14** in $(CD_3)_2SO$ S52
S41: ^{13}C NMR spectrum of compound **14** in $(CD_3)_2SO$ S53
S42: NMR data table for compound **15** S54
S43: ^{1}H NMR spectrum of compound **15** in $(CD_3)_2SO$ S55
S44: ^{13}C NMR spectrum of compound **15** in $(CD_3)_2SO$ S56

S1: Experimental methods

General experimental

Melting points were measured using a Cole-Parmer (Chicago, IL, USA) melting point apparatus and were uncorrected. UV spectra were recorded using an Ocean Optics (USB-ISS-UV/VIS) spectrophotometer. NMR spectra were recorded at 25 °C on a Bruker (Billerica, MA, USA) AVANCE III™ HD 500 MHz NMR spectrometer equipped with a cryoprobe. MestreNova™ version 14.3.3 software was used for NMR data analysis. The ^{1}H and ^{13}C chemical shifts were referenced to solvent peaks for $(CD_3)_2SO$ (δ_H 2.50, δ_C 39.52). LRESIMS data was recorded on a Thermo Scientific (Waltham, MA, USA) UltiMate™ 3000 RS UHPLC coupled to a Thermo Scientific ISQ™ EC single quadruple ESI mass spectrometer. HRESIMS data were acquired on a Bruker maXis II ETD ESI-qTOF. TLC was carried out on Merck (Kenilworth, NJ, USA) silica gel 60 F254 pre-coated aluminium plates and developed plates were visualised using UV light at 254 and 365 nm.

Reaction mixtures were pre-adsorbed onto Merck silica gel (40–63 µm, 143 Å) and Isolute™ silica (30 × 40 mm, 10 g, 55 µm, 54 Å) SPE cartridges were used for normal-phase small-scale separations. Alltech (Lexington, KY, USA) Davisil™ C_{18}-bonded silica (35–70 µm, 60 Å) were used for pre-adsorption work before reversed-phase HPLC separations. Pre-adsorbed material was packed into an Alltech stainless steel guard cartridge (10 × 30 mm) then attached to an HPLC column prior to fractionation. Thermo Electron Betasil™ C_{18}-bonded silica (5 µm, 100 Å, 150 × 21.2 mm) columns were used for reversed-phase HPLC separations. A Thermo Scientific Dionex Ultimate™ 3000 UHPLC was used for HPLC separations. All solvents used for chromatography and MS were Honeywell Burdick & Jackson (Muskegon, MI, USA) or

RCI Labscan (Bangkok, Thailand) HPLC grade. H_2O was filtered using a Sartorius (Gottingen, Lower Saxony, Germany) Arium™ Pro VF ultrapure water system. Synthetic reagents were purchased from Sigma-Aldrich (St. Louis, MO, USA) and used without further purification. The starting material, 5-chloro-3-(4-chlorophenyl)-[1,2,4]triazolo[4,3-*a*]pyrazine (**1**), was previously synthesised and purified by the Davis group [1].

Crystallography studies

Intensity data for compounds **2**, **7**, **10** and **15** were collected with an Oxford Diffraction Synergy diffractometer with Cu or Mo Kα radiation, and the temperature during data collection was maintained at 100.0(1) K, but 250(1) K for **7**, which underwent a destructive phase change at lower temperatures, using an Oxford Cryosystems cooling device. The structures of **2**, **7**, **10** and **15** were solved by direct methods and difference Fourier synthesis. Hydrogen atoms bound to the carbon atoms were placed at their idealised positions using appropriate HFIX instructions in SHELXL [2] and included in subsequent refinement cycles. The hydrogen atom attached to nitrogen was located from difference Fourier maps and refined freely with isotropic displacement parameters. Thermal ellipsoid plots were generated using the program Mercury [3] integrated within the WINGX suite of programs [4]. Complete crystallographic data for compounds **2** (CCDC 2406262), **7** (CCDC 2420136), **10** (CCDC 2420137) and **15** (CCDC 2420244) have been deposited with the Cambridge Crystallographic Data Centre. These data can be obtained free of charge from the Cambridge Crystallographic Data Centre via http://www.ccdc.cam.ac.uk/data_request/cif.

3-(4-Chlorophenyl)-*N*-phenethyl-[1,2,4]triazolo[4,3-*a*]pyrazin-8-amine (2)

$C_{19}H_{16}ClN_5$, *M* = 349.82, *T* = 100.0(1) K, λ = 10.71073 Å, triclinic, space group *P*-1 *a* = 9.53590(1) Å, *b* = 9.6391(2) Å, *c* = 18.9247(3) Å, α= 91.480(1) β= 95.492(1)° γ = 103.950(1)° *V* = 1678.30(5) Å3, *Z* = 4, *Z'* = 2, D_c = 1.384 mg M^{-3} *μ* (Mo-K*α*) 0.239 mm^{-1}, F(000) = 728, crystal size 0.60 x 0.27 x 0.23 mm^3; reflections measured, 21295, θmax = 41.17°, 57839 independent reflections (R_{int} = 0.0356); final R = 0.0455 [I > 2σ(I), 15417data] and $wR(F^2)$ = 0.1413 (all data); GOOF = 1.116.

3-(4-Chlorophenyl)-*N*-isopentyl-[1,2,4]triazolo[4,3-*a*]pyrazin-8-amine (7)

C_{16} H_{18} Cl N_5, *M* = 315.80, *T* = 250.0(1) K, λ = 1.54184 Å, triclinic, space group *P*-1 *a* = 6.8541(2) Å, *b* = 10.4434(3) Å, *c* = 11.7545(3) Å, α= 92.017(2) β= 105.095(2)° γ = 93.263(2)° *V* = 809.95(4) $Å^3$, *Z* = 2, D_c = 1.295 mg M^{-3} *μ* (Cu- K*α*) 2.113 mm^{-1}, F(000) = 332, crystal size 0.28 × 0.06 × 0.04 mm^3; reflections measured, 8861, θmax = 79.335°, 3372 independent reflections (R_{int} = 0.0339); final R = 0. 0448 [I > 2σ(I), 2895 data] and $wR(F^2)$ = 0. 1320 (all data); GOOF = 1.070.

***N*¹-(3-(4-Chlorophenyl)-[1,2,4]triazolo[4,3-*a*]pyrazin-8-yl)-*N*²,*N*²-dimethylethane-1,2-diamine (10)**

C_{15} H_{17} Cl N_6, *M* = 316.79, *T* = 100.0(1) K, λ = 1.54184 Å, triclinic, space group *P*-1 *a* = 7.3282(3) Å, *b* = 12.6354(6) Å, *c* = 17.5968(7) Å, α= 69.251(4) β= 87.931(3)° γ = 85.826(3)° *V* = 1519.56(12) $Å^3$, *Z* = 4, *Z'* =2, D_c = 1.385 mg M^{-3} *μ* (Cu-K*α*) 2.274 mm^{-1}, F(000) = 664, crystal size 0.49 x 0.27 x 0.06 mm^3; reflections measured, 19842, θmax = 80.025°, 6344 independent reflections (R_{int} = 0.0463); final R = 0. 0479 [I > 2σ(I), 15417 data] and $wR(F^2)$ = 0. 1356 (all data); GOOF = 1.091.

***Tert*-butyl(4-((3-(4-chlorophenyl)-[1,2,4]triazolo[4,3-*a*]pyrazin-8 yl)amino)butyl)carbamate (15)**

$C_{20}H_{25}ClN_6O_2$, *M* = 416.91, *T* = 100.0(1) K, λ = 1.54184 Å, triclinic, space group *P*-1 *a* = 9.39890(10) Å, *b* = 10.22800(10) Å, *c* = 22.5357(3) Å, α = 69.251(4) β = 87.931(3)° γ = 102.5610(10)° *V* = 2050.26(4) $Å^3$, *Z* = 4, *Z'* =2, D_c = 1.351 mg M^{-3} *μ* (Cu-K*α*) 1.893 mm^{-1}, F(000) = 880, crystal size 0.52 × 0.13 × 0.07 mm^3; reflections measured, 24392, θmax = 79.437°, 8526 independent reflections (R_{int} = 0.0281); final R = 0. 0638 [I > 2σ(I), 7616 data] and $wR(F^2)$ = 0. 1811 (all data); GOOF = 1.092.

In vitro antiplasmodial image-based assay

Plasmodium falciparum 3D7 were cultured in RPMI1640 (Life Technologies, Camarillo, CA, USA) supplemented with 2.5 mg/mL Albumax II, 5% AB human serum, 25 mM HEPES, and 0.37mM hypoxanthine. Human red blood cells (RBC) (O+) were supplied by Australian Red Cross LifeBlood in accordance with agreement 23-05QLD-23. Use of human RBC for anti-plasmodial experimentation was in accordance with Griffith University Human Ethics Exemption Approval #03/08/11019. Ring stage parasites were treated with compounds following two rounds of sorbitol synchronisation, as previously described [5]. Puromycin, chloroquine, pyrimethamine, and dihydroartemisinin were used as reference compounds. Following incubation of assay plates for 72 h at 37 °C, and 5% CO_2 and 5% O_2, parasites were stained with 2-(4-amidinophenyl)-1*H*-indole-6-carboxamidine (DAPI) and imaged using an Opera PhenixPlus™ High Content Screening System (PerkinElmer, Waltham, MA, USA). Images were analysed using Harmony software (PerkinElmer, Waltham, MA, USA).

In vitro cytotoxicity assay

Human embryonic kidney (HEK293) cells were maintained in DMEM (Life Technologies, Camarillo, CA, USA) containing 10% FBS (Hyclone™ ThermoFisher, Melbourne, Australia). Cytotoxicity testing was undertaken as previously described [6]. In brief, 5 µL of test compound was added to the well of black/clear tissue culture-treated, 384-well plates containing 2000 adherent HEK293 cells/well and incubated for 72 h at 37 °C in 5% CO_2. At 6 h, 5 µL of 600 µM resazurin, diluted in growth media, was added. Plates were further incubated for 6 h and measured for fluorescence at 530 nm excitation and 595 nm emission. The % inhibition was calculated using 0.4% DMSO (no inhibition) and 50 µM puromycin (100% inhibition) data. Puromycin was included as a reference compound to assess assay validity. IC_{50} values were obtained by plotting % inhibition against log dose using GraphPad Prism v.6 (San Diego, CA, USA) nonlinear regression with a variable slope plot.

Synthesis of 3-(4-chlorophenyl)-*N*-phenethyl-[1,2,4]triazolo[4,3-*a*]pyrazin-8-amine (2) using modified Korsic et al. method [7]

5-Chloro-3-(4-chlorophenyl)-[1,2,4]triazolo[4,3-*a*]pyrazine (**1**, 106 mg, 0.4 mmol), phenethylamine (150 µL, 1.2 mmol, 3.0 equiv), and silica (0.5 g) were dissolved in anhydrous $PhCH_3$ (2 mL) and the reaction mixture was stirred at room temperature for 6 h then pre-adsorbed to silica (≈1 g) overnight. Purification was performed on a silica Isolute™ SPE cartridge using a 5% stepwise elution from 100% CH_2Cl_2 to 20% MeOH/80% CH_2Cl_2 with five fractions (10 × 5 mL) collected for each flush (50 mL in total). The 25 resulting fractions were all analysed by silica TLC (solvent system: 7.5% MeOH/92.5% CH_2Cl_2) and UV-active (254 nm) samples were further evaluated by ^1H NMR spectroscopy and LCMS to identify product of interest; only fractions containing

the desired product in high purity (≥ 95%) were combined. Some fractions were not of desired purity (≥95%) and were subjected to further reversed-phase HPLC separation using a 10% linear gradient from 50% MeOH/50% H_2O/0.1% TFA to 100% MeOH/0.1% TFA over 60 min at a flow rate of 9 mL/min and again analysed by ^{1}H NMR and LCMS. Pure fractions obtained from reversed-phase HPLC separation were then combined to obtain the desired product, 3-(4-chlorophenyl)-N-phenethyl-[1,2,4]triazolo[4,3-*a*]pyrazin-8-amine (**2**) as yellow needles (124 mg, 70%).

Modified synthesis of 3-(4-chlorophenyl)-*N*-phenethyl-[1,2,4]triazolo[4,3-*a*]pyrazin-8-amine (2)

5-Chloro-3-(4-chlorophenyl)-[1,2,4]triazolo[4,3-*a*]pyrazine (**1**, 106 mg, 0.4 mmol), was dissolved in phenethylamine (500 µL, 1.6 mmol, 10 equiv) and the reaction mixture stirred at room temperature for 16 h then pre-adsorbed to silica (≈1 g) overnight. Purification was performed as described above. Pure fractions (≥95%) obtained from reversed-phase HPLC separation were then combined to obtain the desired product, 3-(4-chlorophenyl)-*N*-phenethyl-[1,2,4]triazolo[4,3-*a*]pyrazin-8-amine (**2**) as yellow needles (115 mg, 82%).

General method for the synthesis of the aminated triazolopyrazine library (3–15)

5-Chloro-3-(4-chlorophenyl)-[1,2,4]triazolo[4,3-*a*]pyrazine (**1**, 53 mg, 0.20 mmol) was dissolved in excess liquid amine (500 µL) and the mixture was stirred at room temperature for 16 h. Purification was performed as described above for **2**, and relevant fractions containing high purity product (≥95%) were combined.

3-(4-Chlorophenyl)-*N*-phenethyl-[1,2,4]triazolo[4,3-*a*]pyrazin-8-amine (2). Yellow needles (115 mg, 82%), mp 224–226 °C. UV (MeOH) λ_{max} (log ε) 250 (4.09), 293 (3.83) nm. ^{1}H NMR (500 MHz, $(CD_3)_2SO$): δ_H 8.30 (1H, t, *J* = 5.8 Hz, H-16), 7.93 (2H, m, H-11, H-15), 7.76 (1H, d, *J* = 4.8 Hz, H-5), 7.69 (2H, m, H-12, H-14), 7.38 (1H, d, *J* = 4.8 Hz, H-6), 7.30 (2H, m, H-21, H-23), 7.28 (2H, m, H-20, H-24), 7.20 (1H, tt, *J* = 6.8, 2.0 Hz, H-22), 3.74 (2H, dt, *J* = 5.8, 7.3 Hz, H-17), 2.98 (2H, t, *J* = 7.3 Hz, H-18). ^{13}C NMR (125 MHz, $(CD_3)_2SO$) δ_C 147.9 (C-8), 146.8 (C-3), 139.7 (C-9), 139.5 (C-19), 135.0 (C-13), 130.3 (C-6), 129.8 (2C, C-11, C-15), 129.4 (2C, C-12, C-14), 128.7 (2C, C-20, C-24), 128.3 (2C, C-21, C-23), 126.1 (C-22), 125.1 (C-10), 106.0 (C-5), 41.6 (C-17), 34.5 (C-18). LRMS (ESI-SQ) *m/z*: 350 $[M + H]^+$, 372 $[M + Na]^+$. HRMS (ESI-qTOF) *m/z*: $[M + Na]^+$ Calcd for $C_{19}H_{16}{}^{35}ClN_5Na$ 372.0991; Found 372.0991.

3-(4-Chlorophenyl)-*N*-(4-fluorophenethyl)-[1,2,4]triazolo[4,3-*a*]pyrazin-8-amine (3). Yellow amorphous solid (51 mg, 69%). UV (MeOH) λ_{max} (log ε) 254 (3.57), 302 (3.56) nm. ^{1}H NMR (500 MHz, $(CD_3)_2SO$) δ_H 8.64 (1H, brt, *J* = 6.1 Hz, H-16), 7.92 (2H, m, H-11, H-15), 7.78 (1H, d, *J* = 4.9 Hz, H-5), 7.69 (2H, m, H-12, H-14), 7.36 (1H, d, *J* = 4.9 Hz, H-6), 7.31 (2H, m, H-20, H-24), 7.12 (2H, m, H-21, H-23), 3.74 (2H, dt, *J* = 6.1, 7.4 Hz, H-17), 2.97 (2H, t, *J* = 7.4 Hz, H-18). ^{13}C NMR (125 MHz, $(CD_3)_2SO$) δ_C 160.8 (d, $^1J_{CF}$= 241.5 Hz, C-22), 147.6 (C-8), 147.1 (C-3), 139.7 (C-9), 135.5 (C-19), 135.1 (C-13), 130.5 (2C, d, $^3J_{CF}$= 8.0 Hz, C-20, C-24), 129.9 (2C, C-11, C-15), 129.4 (2C, C-12, C-14), 128.9 (C-6), 125.0 (C-10), 115.0 (2C, d, $^2J_{CF}$= 20.9 Hz, C-21, C-23), 106.4 (C-5), 41.9 (C-17), 33.4 (C-18). LRMS (ESI-SQ) *m/z*: 368 $[M + H]^+$, 390 $[M + Na]^+$, 757 $[2M + Na]^+$. HRMS (ESI-qTOF) *m/z*: $[M + Na]^+$ Calcd for $C_{19}H_{15}{}^{35}ClFN_5Na$ 390.0892; Found 390.0895.

***N*-Benzyl-3-(4-chlorophenyl)-[1,2,4]triazolo[4,3-*a*]pyrazin-8-amine (4)**. Yellow amorphous solid (31 mg, 46%). UV (MeOH) λ_{max} (log ε) 251 (4.14), 295 (3.83) nm. ^{1}H NMR (500 MHz, $(CD_3)_2SO$) δ_H 9.12 (1H, brt, *J* = 5.4 Hz, H-16), 7.93 (2H, m, H-11, H-15), 7.79 (1H, d, *J* = 4.9 Hz, H-5), 7.70 (2H, m, H-12, H-14), 7.38 (2H, m, H-19, H-23), 7.32 (1H, d, *J* = 4.9 Hz, H-6), 7.30 (2H, m, H-20, H-22), 7.23 (1H, tt, *J* = 1.3, 7.3 Hz, H-21), 4.74 (2H, d, *J* = 5.4 Hz, H-17). ^{13}C NMR (125 MHz, $(CD_3)_2SO$) δ_C 147.6 (C-8), 147.2 (C-3), 139.8 (C-9), 139.1(C-18), 135.1(C-13), 129.9 (2C, C-11, C-15), 129.4 (2C, C-12, C-14), 128.9 (C-6), 128.3 (2C, C-20, C-22), 127.2 (2C, C-19, C-23), 126.8 (C-21), 125.0 (C-10), 106.7 (C-5), 43.4 (C-17). LRMS (ESI-SQ) *m/z*: 336 $[M + H]^+$, 358 $[M + Na]^+$. HRMS (ESI-qTOF) *m/z*: $[M + Na]^+$ Calcd for $C_{18}H_{14}{}^{35}ClN_5Na$ 358.0830; Found 358.0830.

3-(4-Chlorophenyl)-*N*-(4-fluorobenzyl)-[1,2,4]triazolo[4,3-*a*]pyrazin-8-amine (5). Yellow amorphous solid (15 mg, 21%). UV (MeOH) λ_{max} (log ε) 253 (4.41), 295 (4.10) nm. ^{1}H NMR (500 MHz, $(CD_3)_2SO$) δ_H 8.87 (1H, brt, *J* = 6.1 Hz, H-16), 7.92 (2H, m, H-11, H-15), 7.77 (1H, d, *J* = 4.9 Hz, H-5), 7.69 (2H, m, H-12, H-14), 7.42 (2H, m, H-19, H-23), 7.32 (1H, d, *J* = 4.9 Hz, H-6), 7.13 (2H, m, H-20, H-22), 4.69 (2H, d, *J* = 6.1 Hz, H-17). ^{13}C NMR (125 MHz, $(CD_3)_2SO$) δ_C 161.1 (d, $^{1}J_{CF}$= 242.0 Hz, C-21), 147.7 (C-8), 146.9 (C-3), 139.7 (C-9), 135.7 (d, $^{4}J_{CF}$= 3.1 Hz, C-18), 135.0 (C-13), 130.0 (C-6), 129.8 (2C, C-11, C-15), 129.4 (2C, C-12, C-14), 129.2 (2C, $^{2}J_{CF}$= 8.1 Hz, C-19, C-23), 125.1 (C-10), 114.8 (2C, $^{2}J_{CF}$= 21.3 Hz, C-20, C-22), 106.5 (C-5), 42.5 (C-17). LRMS (ESI-SQ) *m/z*: 354 $[M + H]^+$, 729 $[2M + Na]^+$. HRMS (ESI-qTOF) *m/z*: $[M + Na]^+$ Calcd for $C_{18}H_{13}{}^{35}ClFN_5Na$ 376.0736; Found 376.0737.

3-(4-Chlorophenyl)-*N*-propyl-[1,2,4]triazolo[4,3-*a*]pyrazin-8-amine (6). Yellow amorphous solid (41 mg, 72%). UV (MeOH) λ_{max} (log ε) 251 (3.78), 298 (3.50) nm. ^{1}H

NMR (500 MHz, $(CD_3)_2SO$): δ_H 8.26 (1H, t, *J* = 5.7 Hz, H-16), 7.92 (2H, m, H-11, H-15), 7.73 (1H, d, *J* = 4.9 Hz, H-5), 7.69 (2H, m, H-12, H-14), 7.34 (1H, d, *J* = 4.9 Hz, H-6), 3.46 (2H, m, H-17), 1.65 (2H, m, H-18), 0.92 (3H, t, *J* = 7.4 Hz, H-19). ^{13}C NMR (125 MHz, $(CD_3)_2SO$) δ_C 148.0 (C-8), 146.8 (C-3), 139.7 (C-9), 134.9 (C-13), 130.3 (C-6), 129.8 (2C, C-11, C-15), 129.4 (2C, C-12, C-14), 125.2 (C-10), 105.8 (C-5), 41.8 (C-17), 21.8 (C-18), 11.4 (C-19). LRMS (ESI-SQ) *m/z*: 288 $[M + H]^+$, 310 $[M + Na]^+$. HRMS (ESI-qTOF) *m/z*: $[M + H]^+$ Calcd for $C_{14}H_{15}{}^{35}ClN_5$ 288.1010; Found 288.1012; $[M + Na]^+$ Calcd for $C_{14}H_{14}{}^{35}ClN_5Na$ 310.0830; Found 310.0831.

3-(4-Chlorophenyl)-*N*-isopentyl-[1,2,4]triazolo[4,3-*a*]pyrazin-8-amine (7). White needles (56 mg, 89%), mp 193–195 °C. UV (MeOH) λ_{max} (log ε) 250 (4.33), 297 (4.05) nm. ^1H NMR (500 MHz, $(CD_3)_2SO$): δ_H 8.22 (1H, brt, *J* = 5.7 Hz, H-16), 7.93 (2H, m, H-11, H-15), 7.73 (1H, d, *J* = 4.8 Hz, H-5), 7.69 (2H, m, H-12, H-14), 7.35 (1H, d, *J* = 4.8 Hz, H-6), 3.52 (2H, m, H-17), 1.65 (1H, m, H-19), 1.54 (2H, m, H-18), 0.92 (6H, d, *J* = 6.6 Hz, H-20, H-21). ^{13}C NMR (125 MHz, $(CD_3)_2SO$) δ_C 148.0 (C-8), 146.8 (C-3), 139.7 (C-9), 134.9 (C-13), 130.3 (C-6), 129.8 (2C, C-11, C-15), 129.3 (2C, C-12, C-14), 125.2 (C-10), 105.7 (C-5), 38.4 (C-17), 37.5 (C-18), 25.4 (C-19), 22.5 (2C, C-20, C-21). LRMS (ESI-SQ) *m/z*: 316 $[M + H]^+$. HRMS (ESI-qTOF) *m/z*: $[M + H]^+$ Calcd for $C_{16}H_{19}{}^{35}ClN_5$ 316.1329; Found 316.1323; $[M + Na]^+$ Calcd for $C_{16}H_{18}{}^{35}ClN_5Na$ 338.1143; Found 338.1142; $[2M + H]^+$ Calcd for $C_{32}H_{37}{}^{35}Cl_2N_{10}$ 631.2580; Found 631.2572.

3-(4-Chlorophenyl)-*N*-cyclohexyl-[1,2,4]triazolo[4,3-*a*]pyrazin-8-amine (8). Yellow amorphous solid (18 mg, 27%). UV (MeOH) λ_{max} (log ε) 251 (4.32), 296 (4.02) nm. ^1H NMR (500 MHz, $(CD_3)_2SO$): δ_H 7.98 (1H, d, *J* = 8.2 Hz, H-16), 7.92 (2H, m, H-11, H-15), 7.72 (1H, d, *J* = 4.8 Hz, H-5), 7.68 (2H, m, H-12, H-14), 7.34 (1H, d, *J* = 4.8 Hz,

H-6), 4.07 (1H, m, H-17), 1.91 (2H, m, H-18, H-22), 1.76 (2H, m, H-19, H-21), 1.63 (1H, m, H-20), 1.46 (2H, m, H-18, H-22), 1.33 (2H, m, H-19, H-21), 1.16 (1H, m, H-20). ^{13}C NMR (125 MHz, $(CD_3)_2SO$) δ_C 147.2 (C-8), 146.8 (C-3), 139.6 (C-9), 134.9 (C-13), 130.3 (C-6), 129.8 (2C, C-11, C-15), 129.3 (2C, C-12, C-14), 125.2 (C-10), 105.7 (C-5), 49.0 (C-17), 31.9 (2C, C-18, C-22), 25.3 (C-20), 25.0 (2C, C-19, C-21). LRMS (ESI-SQ) *m/z*: 328 $[M + H]^+$. HRMS (ESI-qTOF) *m/z*: $[M + H]^+$ Calcd for $C_{17}H_{19}{}^{35}ClN_5$ 328.1324; Found 328.1326.

3-(4-Chlorophenyl)-*N*-(2-methoxyethyl)-[1,2,4]triazolo[4,3-*a*]pyrazin-8-amine (9). Yellow amorphous solid (34 mg, 57%). UV (MeOH) λ_{max} (log ε) 253 (4.37), 294 (4.08) nm. ^{1}H NMR (500 MHz, $(CD_3)_2SO$): δ_H 8.13 (1H, t, *J* = 5.8 Hz, H-16), 7.92 (2H, m, H-11, H-15), 7.76 (1H, d, *J* = 4.8 Hz, H-5), 7.69 (2H, m, H-12, H-14), 7.35 (1H, d, *J* = 4.8 Hz, H-6), 3.68 (2H, dt, *J* = 5.8, 6.2 Hz, H-17), 3.57 (2H, t, *J* = 6.2 Hz, H-18), 3.28 (3H, s, H-20). ^{13}C NMR (125 MHz, $(CD_3)_2SO$) δ_C 148.0 (C-8), 146.8 (C-3), 139.7 (C-9), 135.0 (C-13), 130.2 (C-6), 129.8 (2C, C-11, C-15), 129.4 (2C, C-12, C-14), 125.1 (C-10), 106.2 (C-5), 69.8 (C-18), 57.9 (C-20), 39.5 (C-17). LRMS (ESI-SQ) *m/z*: Found 304 $[M + H]^+$. HRMS (ESI-qTOF) m/z: $[M + Na]^+$ Calcd for $C_{14}H_{14}{}^{35}ClN_5NaO$ 326.0779; Found 326.0776; $[2M + Na]^+$ Calcd for $C_{28}H_{28}{}^{35}Cl_2N_{10}NaO_2$ 629.1671; Found 629.1662.

***N*¹-(3-(4-Chlorophenyl)-[1,2,4]triazolo[4,3-*a*]pyrazin-8-yl)-*N*²,*N*²-dimethylethane-1,2-diamine (10)**. Pale yellow needles (43 mg, 68%), mp 153–155 °C. UV (MeOH) λ_{max} (log ε) 253 (4.36), 294 (4.08) nm. ^{1}H NMR (500 MHz, $(CD_3)_2SO$): δ_H 7.95 (1H, t, *J* = 5.5 Hz, H-16), 7.91 (2H, m, H-11, H-15), 7.74 (1H, d, *J* = 4.8 Hz, H-5), 7.67 (2H, m, H-12, H-14), 7.34 (1H, d, *J* = 4.8 Hz, H-6), 3.60 (2H, dt, *J* = 5.5, 6.6 Hz, H-17), 2.55 (2H, t, *J* = 6.6 Hz, H-18), 2.22 (6H, s, H-20, H-21). ^{13}C NMR (125 MHz, $(CD_3)_2SO$) δ_C

148.0 (C-8), 146.8 (C-3), 139.7 (C-9), 135.0 (C-13), 130.3 (C-6), 129.8 (2C, C-11, C-15), 129.4 (2C, C-12, C-14), 125.1 (C-10), 106.0 (C-5), 57.4 (C-18), 45.1 (2C, C-20, C-21), 37.9 (C-17). LRMS (ESI-SQ) *m/z*: 317 $[M + H]^+$. HRMS (ESI-qTOF) *m/z*: $[M + H]^+$ Calcd for $C_{15}H_{18}{}^{35}ClN_6$ 317.1276; Found 317.1279.

***N*^{1}-(3-(4-Chlorophenyl)-[1,2,4]triazolo[4,3-a]pyrazin-8-yl)-*N*^{2},*N*^{2}-diethylethane-1,2-diamine (11)**. Pale yellow amorphous solid (19 mg, 27%). UV (MeOH) λ_{max} (log ε) 253 (4.38), 292 (4.10) nm. ^{1}H NMR (500 MHz, $(CD_3)_2SO$) δ_H 7.94 (1H, t, *J* = 5.8 Hz, H-16), 7.92 (2H, m, H-11, H-15), 7.75 (1H, d, *J* = 4.8 Hz, H-5), 7.68 (2H, m, H-12, H-14), 7.35 (1H, d, *J* = 4.8 Hz, H-6), 3.57 (2H, dt, *J* = 5.8, 7.1 Hz, H-17), 2.69 (2H, t, *J* = 7.1 Hz, H-18), 2.55 (4H, q, *J* = 7.1 Hz, H-20, H-22), 0.98 (6H, t, *J* = 7.1 Hz, H-21, H-23). ^{13}C NMR (125 MHz, $(CD_3)_2SO$) δ_C 148.0 (C-8), 146.8 (C-3), 139.7 (C-9), 135.0 (C-13), 130.3 (C-6), 129.8 (2C, C-11, C-15), 129.4 (2C, C-12, C-14), 125.1 (C-10), 106.0 (C-5), 50.8 (C-18), 46.6 (2C, C-10, C-22), 38.0 (C-17), 11.9 (2C, C-21, C-23). LRMS (ESI-SQ) *m/z*: 345 $[M + H]^+$. HRMS (ESI-qTOF) *m/z*: $[M + H]^+$ Calcd for $C_{17}H_{22}{}^{35}ClN_6$ 345.1589; Found 345.1599; $[M + Na]^+$ Calcd for $C_{17}H_{21}{}^{35}ClN_6Na$ 367.1408; Found 367.1409.

***N*^{1}-(3-(4-Chlorophenyl)-[1,2,4]triazolo[4,3-*a*]pyrazin-8-yl)-*N*^{3},*N*^{3}-dimethylpropane-1,3-diamine (12).** Yellow amorphous solid (38 mg, 57%). UV (MeOH) λ_{max} (log ε) 253 (4.02), 295 (3.75) nm. ^{1}H NMR (500 MHz $(CD_3)_2SO$): δ_H 8.34 (1H, brt, *J* = 5.5 Hz, H-16), 7.92 (2H, m, H-11, H-15), 7.75 (1H, d, *J* = 4.8 Hz, H-5), 7.69 (2H, m, H-12, H-14), 7.35 (1H, d, *J* = 4.8 Hz, H-6), 3.54 (2H, m, H-17), 2.49 (2H, t, *J* = 7.1 Hz, H-19), 2.29 (6H, s, H-21, H-22), 1.83 (2H, m, H-18). ^{13}C NMR (125 MHz, $(CD_3)_2SO$) δ_C 148.0 (C-8), 146.8 (C-3), 139.7 (C-9), 135.0 (C-13), 130.3 (C-6), 129.8 (2C, C-11, C-15), 129.4 (2C, C-12, C-14), 125.2 (C-10), 105.9 (C-5), 56.5 (C-19), 44.5

(2C, C-21, C-22), 38.4 (C-17), 25.7 (C-18). LRMS (ESI-SQ) *m/z*: 331 $[M + H]^+$. HRMS (ESI-qTOF) *m/z*: $[M + H]^+$ Calcd for $C_{16}H_{20}{}^{35}ClN_6$ 331.1432; Found 331.1441.

***N*^{1}-(3-(4-Chlorophenyl)-[1,2,4]triazolo[4,3-*a*]pyrazin-8-yl)-*N*^{3},*N*^{3}-diethylpropane-1,3-diamine (13).** Yellow amorphous solid (62 mg, 87%). UV (MeOH) λ_{max} (log ε) 252 (4.50), 292 (4.24) nm. ^{1}H NMR (500 MHz, $(CD_3)_2SO$): δ_H 8.44 (1H, t, *J* = 5.3 Hz, H-16), 7.91 (2H, m, H-11, H-15), 7.73 (1H, d, *J* = 4.8 Hz, H-5), 7.66 (2H, m, H-12, H-14), 7.33 (1H, d, *J* = 4.8 Hz, H-6), 3.55 (2H, m, H-17), 2.65 (2H, t, *J* = 8.0 Hz, H-19), 2.62 (4H, q, *J* = 7.2 Hz, H-21, H-23), 1.84 (2H, m, H-18), 1.03 (6H, t, *J* = 7.2 Hz, H-22, H-24). ^{13}C NMR (125 MHz, $(CD_3)_2SO$) δ_C 148.0 (C-8), 146.7 (C-3), 139.7 (C-9), 134.9 (C-13), 130.3 (C-6), 129.7 (2C, C-11, C-15), 129.3 (2C, C-12, C-14), 125.1 (C-10), 105.9 (C-5), 50.1 (C-19), 46.2 (2C, C-21, C-23), 38.9 (C-17), 24.8 (C-18), 10.7 (2C, C-22, C-24). LRMS (ESI-SQ) *m/z*: 359 $[M + H]^+$. HRMS (ESI-qTOF) *m/z*: $[M + Na]^+$ Calcd for $C_{18}H_{23}{}^{35}ClN_6Na$ 381.1565; Found 381.1564.

3-(4-Chlorophenyl)-*N*-(2-(piperazin-1-yl)ethyl)-[1,2,4]triazolo[4,3-*a*]pyrazin-8-amine (14). White amorphous solid (13 mg, 18%). UV (MeOH) λ_{max} (log ε) 256 (4.04), 304 (3.86) nm. ^{1}H NMR (500 MHz, $(CD_3)_2SO$): δ_H 7.88 (2H, m, H-11, H-15), 7.81 (1H, d, *J* = 4.7 Hz, H-5), 7.69 (2H, m, H-12, H-14), 7.40 (1H, d, *J* = 4.7 Hz, H-6), 4.26 (4H, m, H-21, H-23), 2.73 (2H, t, *J* = 6.4 Hz, H-17), 2.54 (4H, m, H-20, H-24), 2.40 (2H, t, *J* = 6.4 Hz, H-18). ^{13}C NMR (125 MHz, $(CD_3)_2SO$) δ_C 147.7 (C-8), 146.3 (C-3), 140.6 (C-9), 135.2 (C-13), 130.1 (2C, C-11, C-15), 129.6 (C-6), 129.4 (2C, C-12, C-14), 125.0 (C-10), 107.0 (C-5), 59.6 (C-18), 52.9 (2C, C-20, C-24), 45.8 (2C, C-21, C-23), 38.0 (C-17). LRMS (ESI-SQ) *m/z*: 358 $[M + H]^+$. HRMS (ESI-qTOF) *m/z*: $[M + H]^+$ Calcd for $C_{17}H_{21}{}^{35}ClN_7$ 358.1541; Found 358.1546; $[M + Na]^+$ Calcd for $C_{17}H_{20}{}^{35}ClN_7Na$ 380.1361; Found 380.1359.

***tert*-Butyl(4-((3-(4-chlorophenyl)-[1,2,4]triazolo[4,3-*a*]pyrazin-8 yl)amino)butyl)carbamate (15).** Yellow needles (47 mg, 57%), mp 154–156 °C. UV (MeOH) λ_{max} (log ε) 250 (4.71), 295 (4.33) nm. ^{1}H NMR (500 MHz, $(CD_3)_2SO$): δ_H 8.24 (1H, brt, *J* = 5.7 Hz, H-16), 7.90 (2H, m, H-11, H-15), 7.71 (1H, d, *J* = 4.8 Hz, H-5), 7.66 (2H, m, H-12, H-14), 7.33 (1H, d, *J* = 4.8 Hz, H-6), 6.79 (1H, t, *J* = 5.6 Hz, H-21), 3.48 (2H, m, H-17), 2.94 (2H, m, H-20), 1.61 (2H, m, H-18), 1.44 (2H, m, H-19), 1.35 (9H, s, H-25, H-26, H-27). ^{13}C NMR (125 MHz, $(CD_3)_2SO$) δ_C 155.7 (C-22), 148.1 (C-8), 146.8 (C-3), 139.8 (C-9), 135.0 (C-13), 130.4 (C-6), 129.8 (2C, C-11, C-15), 129.4 (2C, C-12, C-14), 125.2 (C-10), 105.8 (C-5), 77.4 (C-24), 39.8 (C-17), 39.4 (C-20), 28.3 (3C, C-25, C-26, C-27), 27.2 (C-19), 26.0 (C-18). LRMS (ESI-SQ) *m/z*: 417 $[M + H]^+$. HRMS (ESI-qTOF) *m/z*: $[M + H]^+$ Calcd for $C_{20}H_{26}{}^{35}ClN_6O_2$ 417.1800; Found 417.1798; $[M + Na]^+$ Calcd for $C_{20}H_{25}{}^{35}ClN_6NaO_2$ 439.1620; Found 439.1616.

S2: Supporting information references

1. Johnson, D. J. G.; Jenkins, I. D.; Huxley, C.; Coster, M. J.; Lum, K. Y.; White, J. M.; Avery, V. M.; Davis, R. A.; *Molecules* **2021,** *26*, 2421.

2. Sheldrick, G.M.; *Acta Crystallogr C Struct Chem.* **2015,** *71*, 3−8.

3. Macrae, C. F.; Bruno, I. J.; Chisholm, J. A.; Edgington, P. R.; McCabe, P.; Pidcock, E.; Rodriguez-Monge, L.; Taylor, R.; Van De Streek, J.; Wood, P. A.; *J. Appl. Crystallogr.* **2008,** *41*, 466−470.

4. Farrugia, L. J.; *J. Appl. Crystallogr.* **1999,** *32***,** 837−838.

5. Duffy, S.; Avery, V. M.; *Am. J. Trop. M. Hyg.* **2012,** *86*, 84−92.

6. Fletcher, S.; Avery, V. M.; *Malar. J.* **2014,** *13*, 343.

7. Korsik, M.; Tse, E.G.; Smith, D. G.; Lewis, W.; Rutledge, P. J.; Todd, M. H.; The *J. Org. Chem.* **2020,** *85*, 13438−13452.

S3: NMR data table for compound **2**[a]

Position	δ_H, mult. (*J* in Hz), int.	δ_C, mult.	COSY	HMBC	ROESY
3		146.8, C			
5	7.76, d (4.8), 1H	106.0, CH	6	3, 6, 9	6, 11, 15
6	7.38, d (4.8), 1H	130.3, CH	5	5, 8	5
8		147.9, C			
9		139.7, C			
10		125.1, C			
11	7.93, m, 1H	129.8, CH	12	3, 13, 15	5, 12
12	7.69, m, 1H	129.4, CH	11	10, 14	11
13		135.0, C			
14	7.69, m, 1H	129.4, CH	15	10, 12	15
15	7.93, m, 1H	129.8, CH	14	3, 11, 13	5, 14
16	8.30, t (5.8), 1H		17	8, 9, 17	17, 18
17	3.74, dt (5.8, 7.3), 2H	41.6, CH_2	16, 18	8, 18, 19	16, 18
18	2.98, t (7.3), 2H	34.5, CH_2	17	17, 19, 20, 24	16, 17, 20, 24
19		139.5, C			
20	7.28, m, 1H	128.7, CH	21, 22	18, 21, 22	18, 21
21	7.30, m, 1H	128.3, CH	20, 22	19, 22, 23	20, 22
22	7.20, tt (6.8, 2.0), 1H	126.1, CH	20, 21, 23, 24	20, 21, 23, 24	21, 23
23	7.30, m, 1H	128.3, CH	22, 24	19, 21, 22	22, 24
24	7.28, m, 1H	128.7, CH	22, 23	18, 20, 22	18, 23

[a] Recorded in $(CD_3)_2SO$ 500 MHz (^1H NMR) and 125 MHz (^{13}C NMR) at 25 °C.

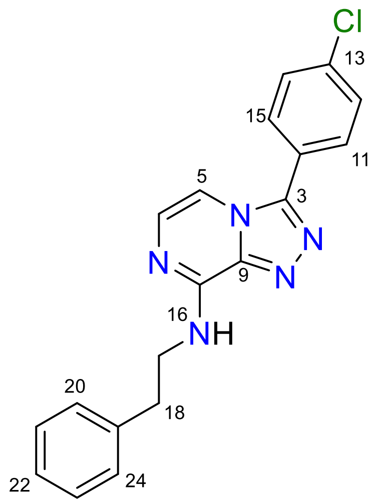

S4: ^{1}H NMR spectrum of compound **2** in $(CD_3)_2SO$

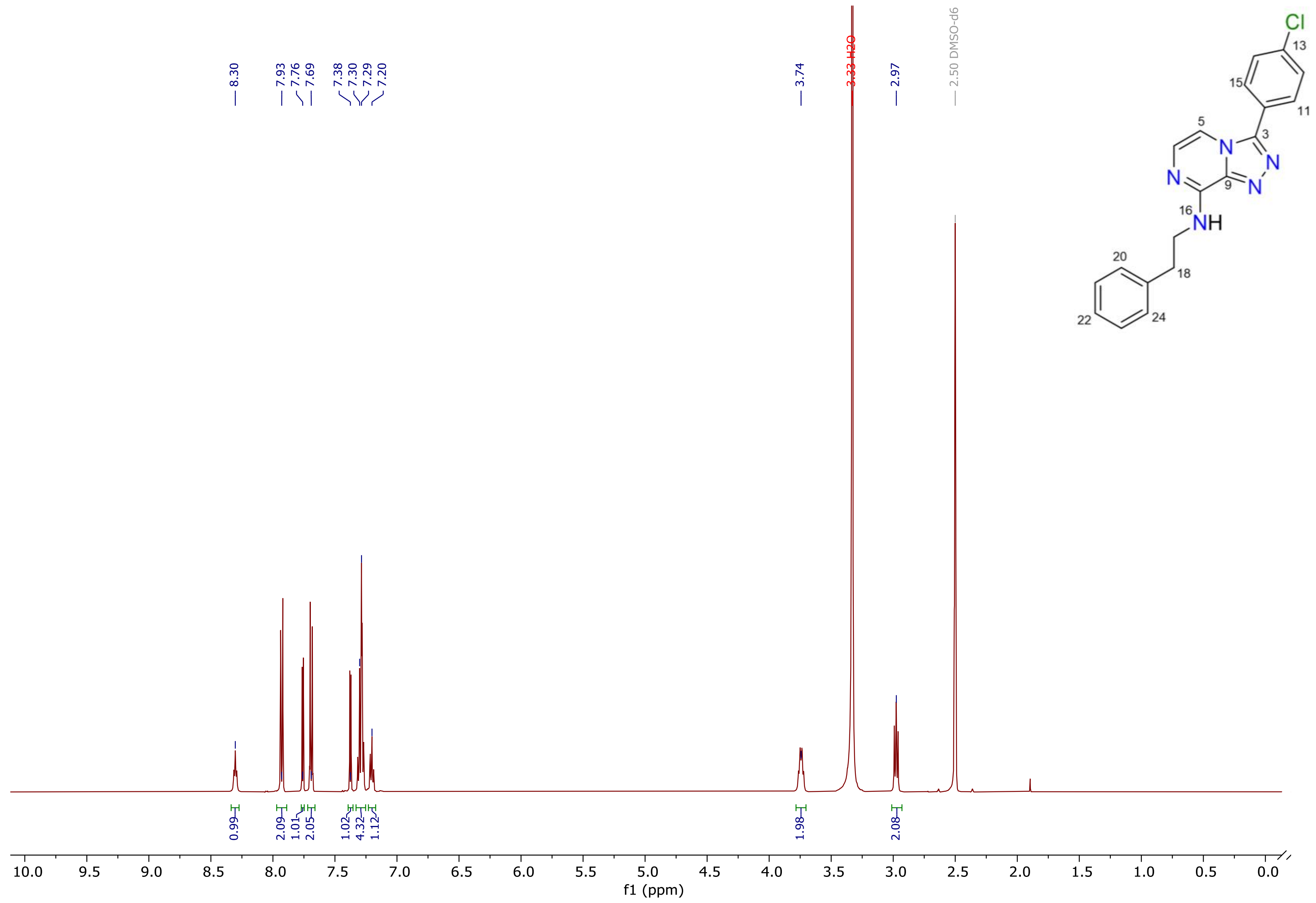

S5: ^{13}C NMR spectrum of compound **2** in $(CD_3)_2SO$

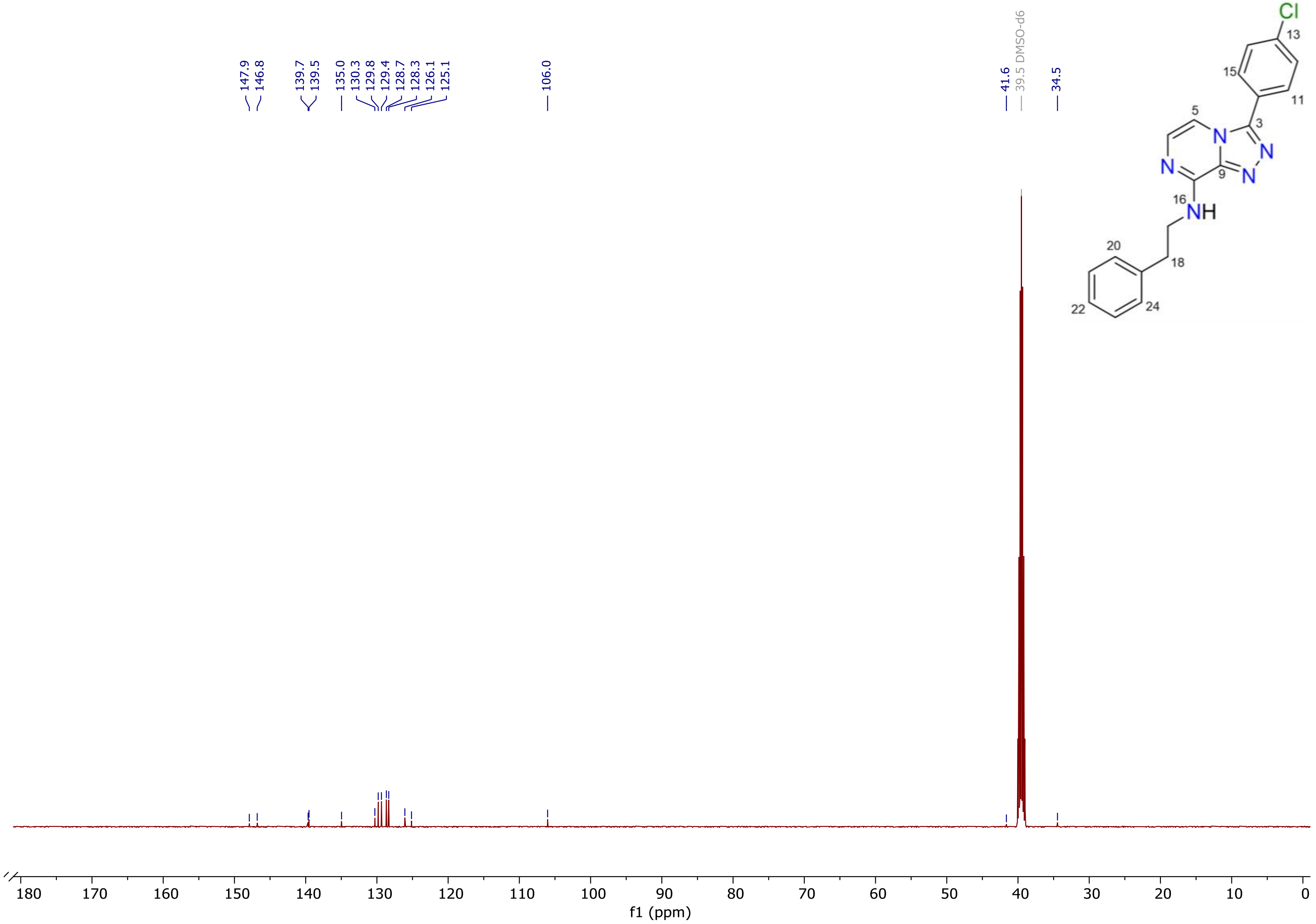

S6: NMR data table for compound **3**[a]

Position	δ_H, mult. (*J* in Hz), int.	δ_C, mult. (*J* in Hz)	COSY	HMBC	ROESY
3		147.1, C			
5	7.78, d (4.9), 1H	106.4, CH	6	3, 6, 9	6, 11, 15
6	7.36, d (4.9), 1H	128.9, CH	5	5, 8	5
8		147.6, C			
9		139.7, C			
10		125.0, C			
11	7.92, m, 1H	129.9, CH	12	3, 13,15	5, 12
12	7.69, m, 1H	129.4, CH	11	10, 14	5, 11
13		135.1, C			
14	7.69, m, 1H	129.4, CH	15	10, 12	5, 15
15	7.92, m, 1H	129.9, CH	14	3, 11, 13	5, 14
16	8.64, brt (6.1), 1H		17		
17	3.74, dt (6.1, 7.4), 2H	41.9, CH_2	16, 18		18, 20, 24
18	2.97, t (7.4), 2H	33.4, CH_2	17	17, 19, 20, 24	17, 20, 24
19		135.5, C			
20	7.31, m, 1H	130.5, d (8.0), CH	21	18, 22, 24	17, 18, 21
21	7.12, m, 1H	115.0, d (20.9), CH	20	19, 23	20
22		160.8, d (241.5), C			
23	7.12, m, 1H	115.0, d (20.9), CH	24	19, 21	24
24	7.31, m, 1H	130.5, d (8.0), CH	23	18, 20, 22,	17, 18, 23

[a] Recorded in $(CD_3)_2SO$ 500 MHz (^1H NMR) and 125 MHz (^{13}C NMR) at 25 °C.

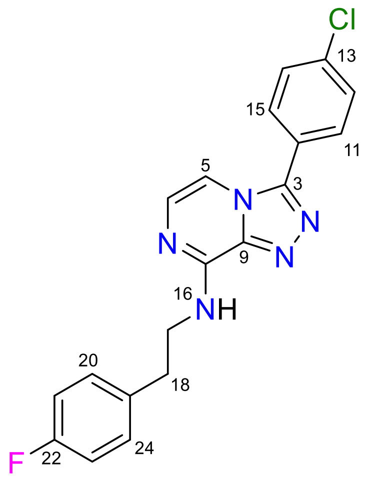

S7: ^{1}H NMR spectrum of compound **3** in $(CD_3)_2SO$

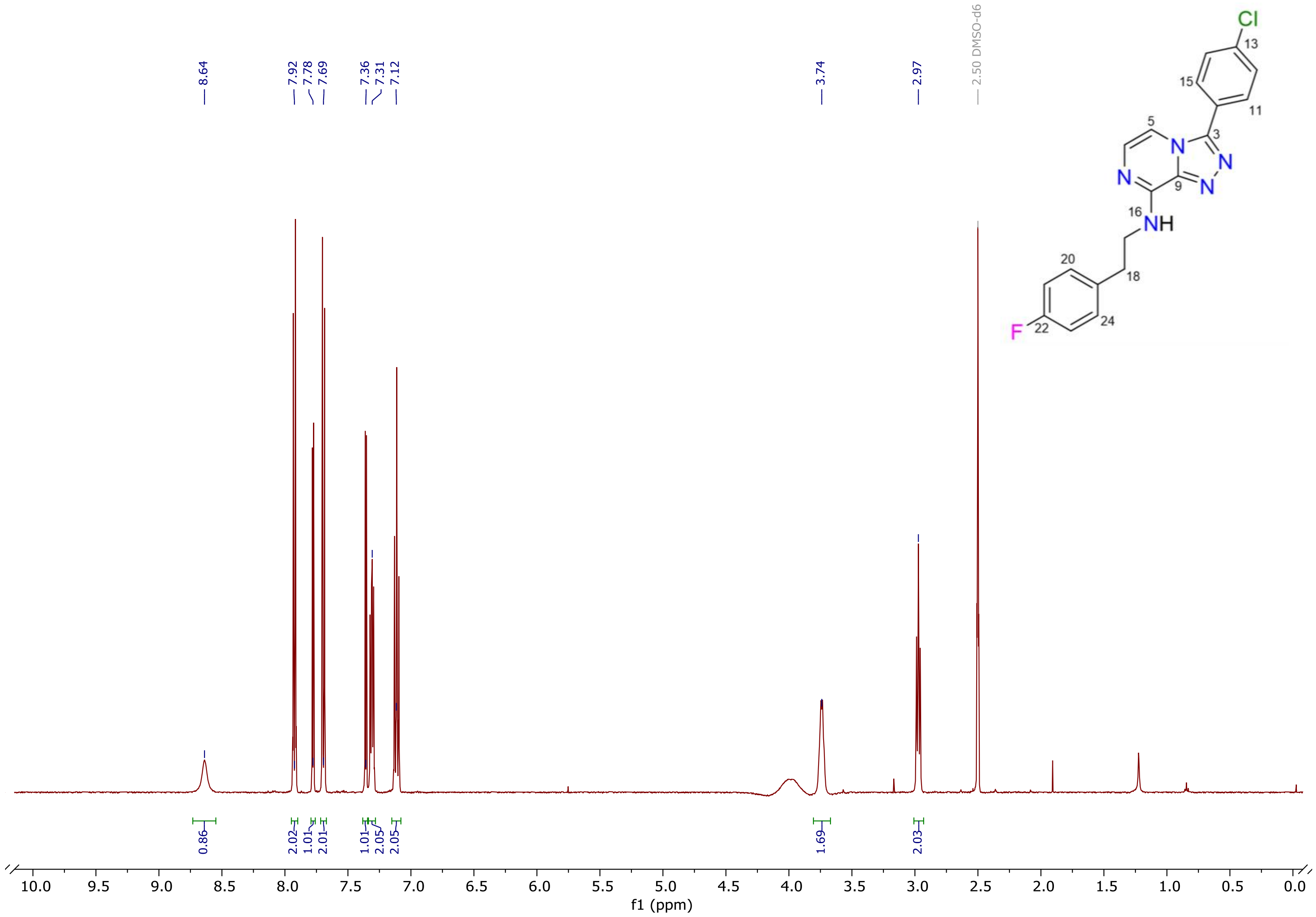

S8: ^{13}C NMR spectrum of compound **3** in $(CD_3)_2SO$

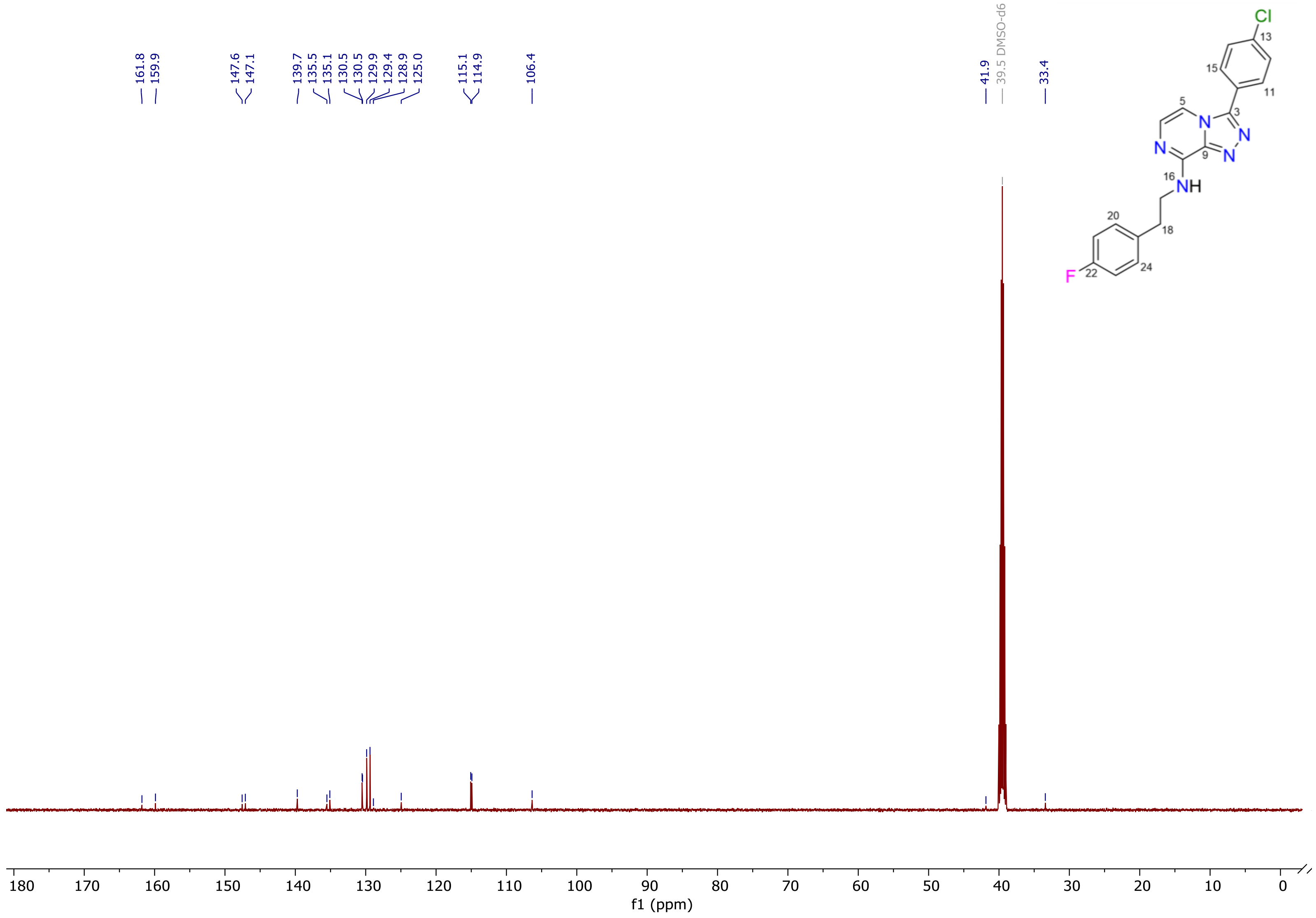

S9: NMR data table for compound **4**[a]

Position	δ_H, mult. (*J* in Hz), int.	δ_C, mult. (*J* in Hz)	COSY	HMBC	ROESY
3		147.2, C			
5	7.79, d (4.9), 1H	106.7, CH	6	3, 6, 9	6, 11, 15
6	7.32, d (4.9), 1H	128.9, CH	5	5, 8	5
8		147.6, C			
9		139.8, C			
10		125.0, C			
11	7.93, m, 1H	129.9, CH	12	3, 13, 15	5, 12, 14
12	7.70, m, 1H	129.4, CH	11	10, 14	11
13		135.1, C			
14	7.70, m, 1H	129.4, CH	15	10, 12	15
15	7.93, m, 1H	129.9, CH	14	3, 11, 13	5, 12, 14
16	9.12, brt (5.4), 1H		17		17
17	4.74, d (5.4), 2H	43.4, CH_2	16		16, 19, 23
18		139.1, C			
19	7.38, m, 1H	127.2, CH	20	17, 21, 23	17, 20
20	7.30, m, 1H	128.3, CH	19, 21	18, 22	
21	7.23, tt (1.3, 7.3), 1H	126.8, CH	19, 20, 22, 23	19, 23	20, 22
22	7.30, m, 1H	128.3, CH	21, 23	18, 20	
23	7.38, m, 1H	127.2, CH	22	17, 19, 21	17, 22

[a] Recorded in $(CD_3)_2SO$ 500 MHz (^1H NMR) and 125 MHz (^{13}C NMR) at 25 °C.

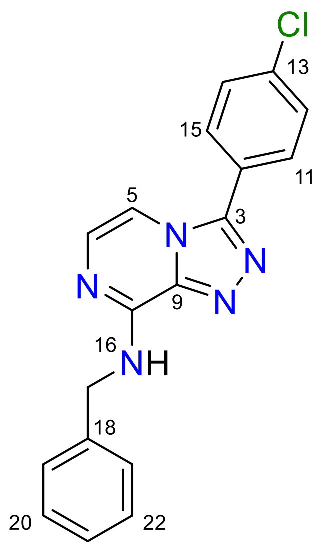

S10: ^{1}H NMR spectrum of compound **4** in $(CD_3)_2SO$

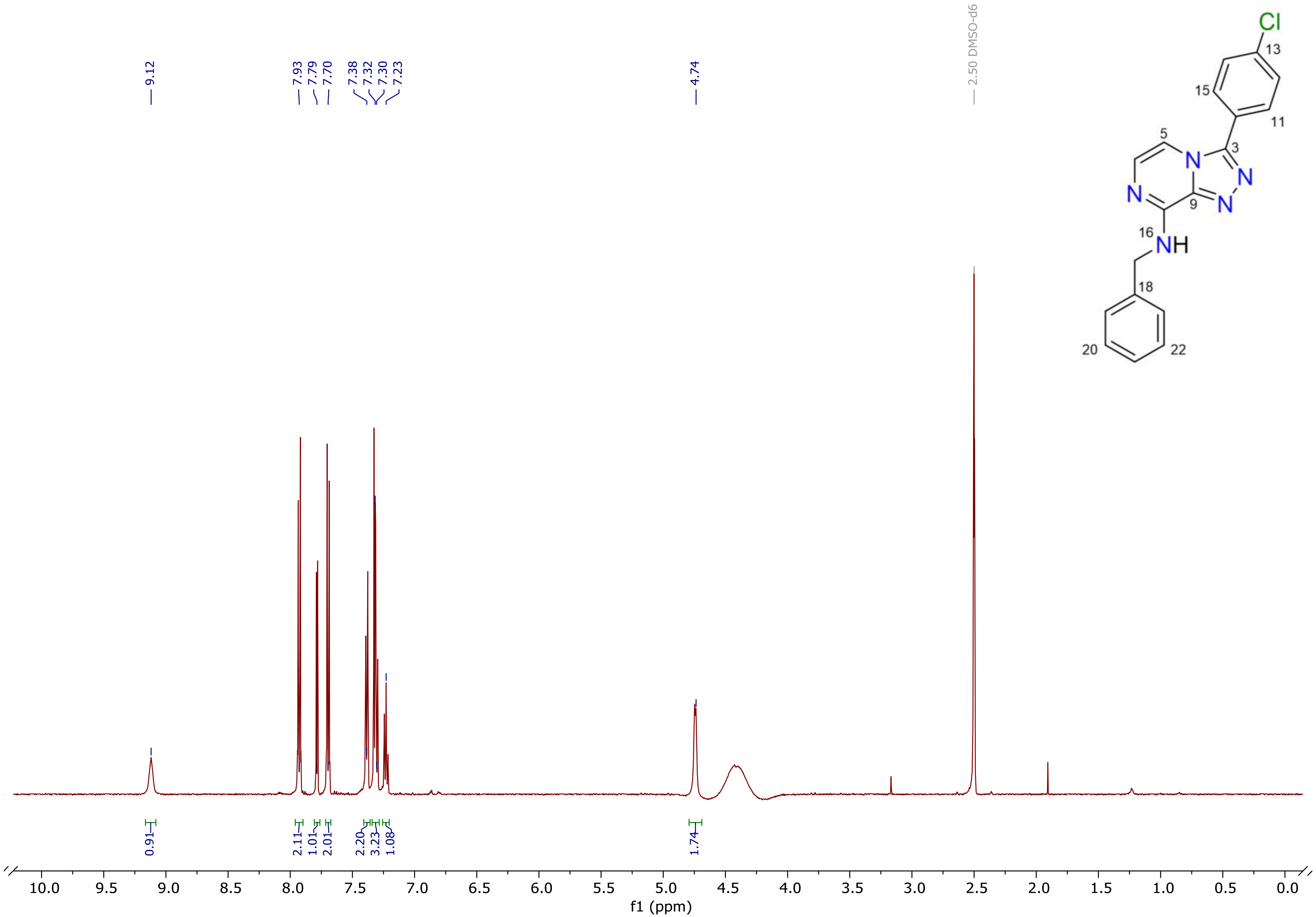

S11: ^{13}C NMR spectrum of compound **4** in $(CD_3)_2SO$

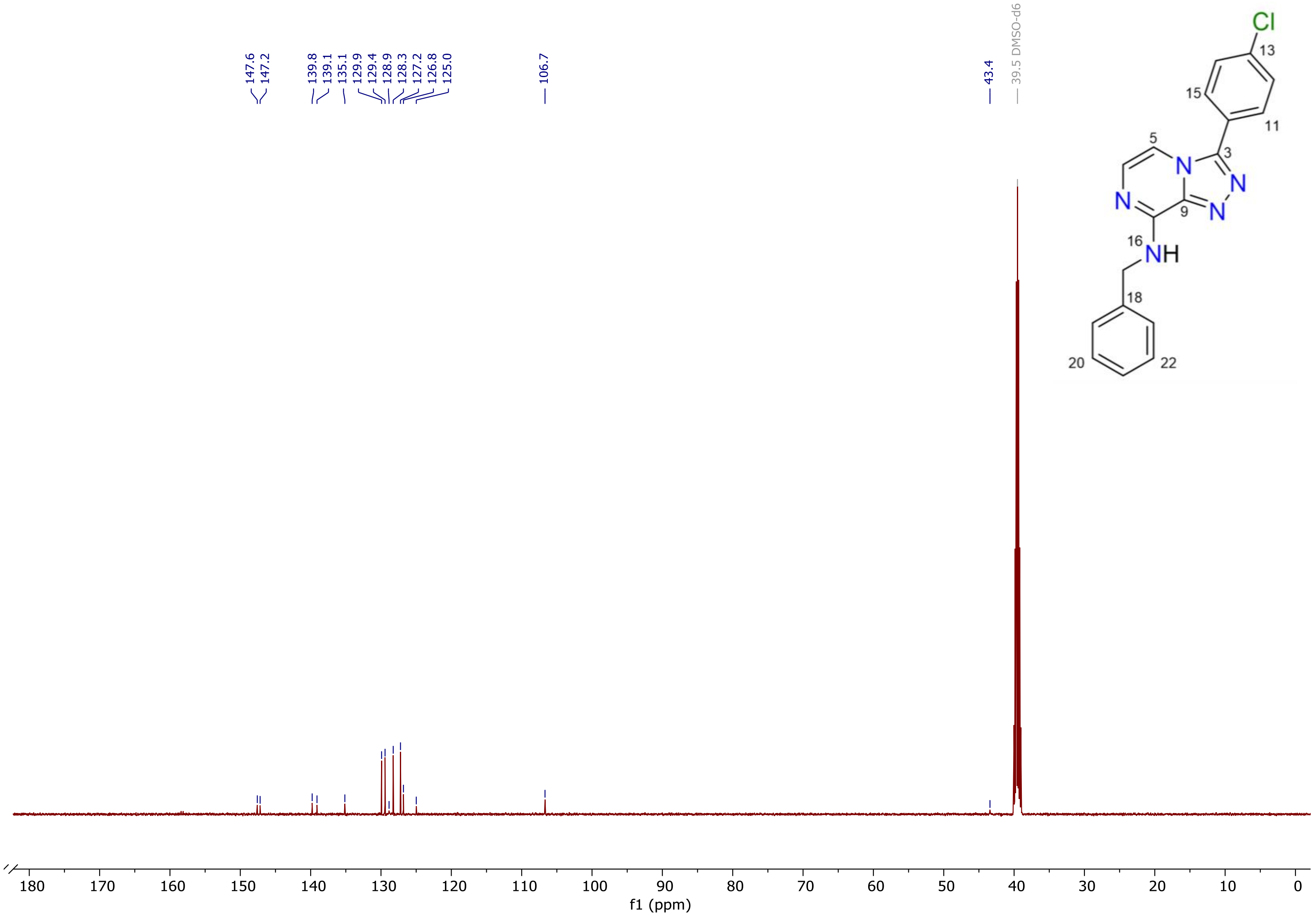

S12: NMR data table for compound **5**[a]

Position	δH, mult. (J in Hz), int.	δC, mult. (J in Hz)	COSY	HMBC	ROESY
3		146.9, C			
5	7.77, d (4.9), 1H	106.5, CH	6	3, 6, 9	6, 11, 15
6	7.32, d (4.9), 1H	130.0, CH	5	5, 8	5
8		147.7, C			
9		139.7, C			
10		125.1, C			
11	7.92, m, 1H	129.8, CH	12	3, 13, 15	12
12	7.69, m, 1H	129.4, CH	11	10, 14	11
13		135.0, C			
14	7.69, m, 1H	129.4, CH	15	10, 12	15
15	7.92, m, 1H	129.8, CH	14	3, 11, 13	14
16	8.87, brt (6.1), 1H		17		17
17	4.69, d (6.1), 2H	42.5, CH_2	16	8, 18, 19, 23	16, 19
18		135.7, d (3.1)			
19	7.42, m, 1H	129.2, d (8.1)	20	17, 21, 23	17, 20
20	7.13, m, 1H	114.8, d (21.3)	19	18, 22	19
21		161.1, d (242.0)			
22	7.13, m, 1H	114.8, d (21.3)	23	18, 20	23
23	7.42, m, 1H	129.2, d (8.1)	22	17, 19, 21	17, 22

[a] Recorded in $(CD_3)_2SO$ 500 MHz (^{1}H NMR) and 125 MHz (^{13}C NMR) at 25 °C.

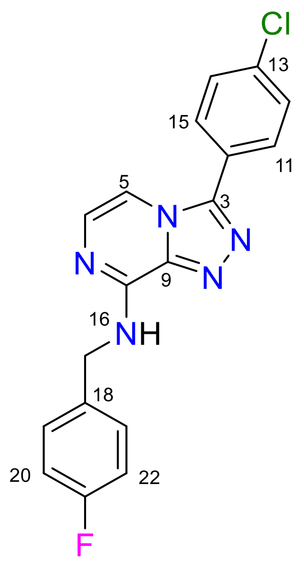

S13: ^{1}H NMR spectrum of compound **5** in $(CD_3)_2SO$

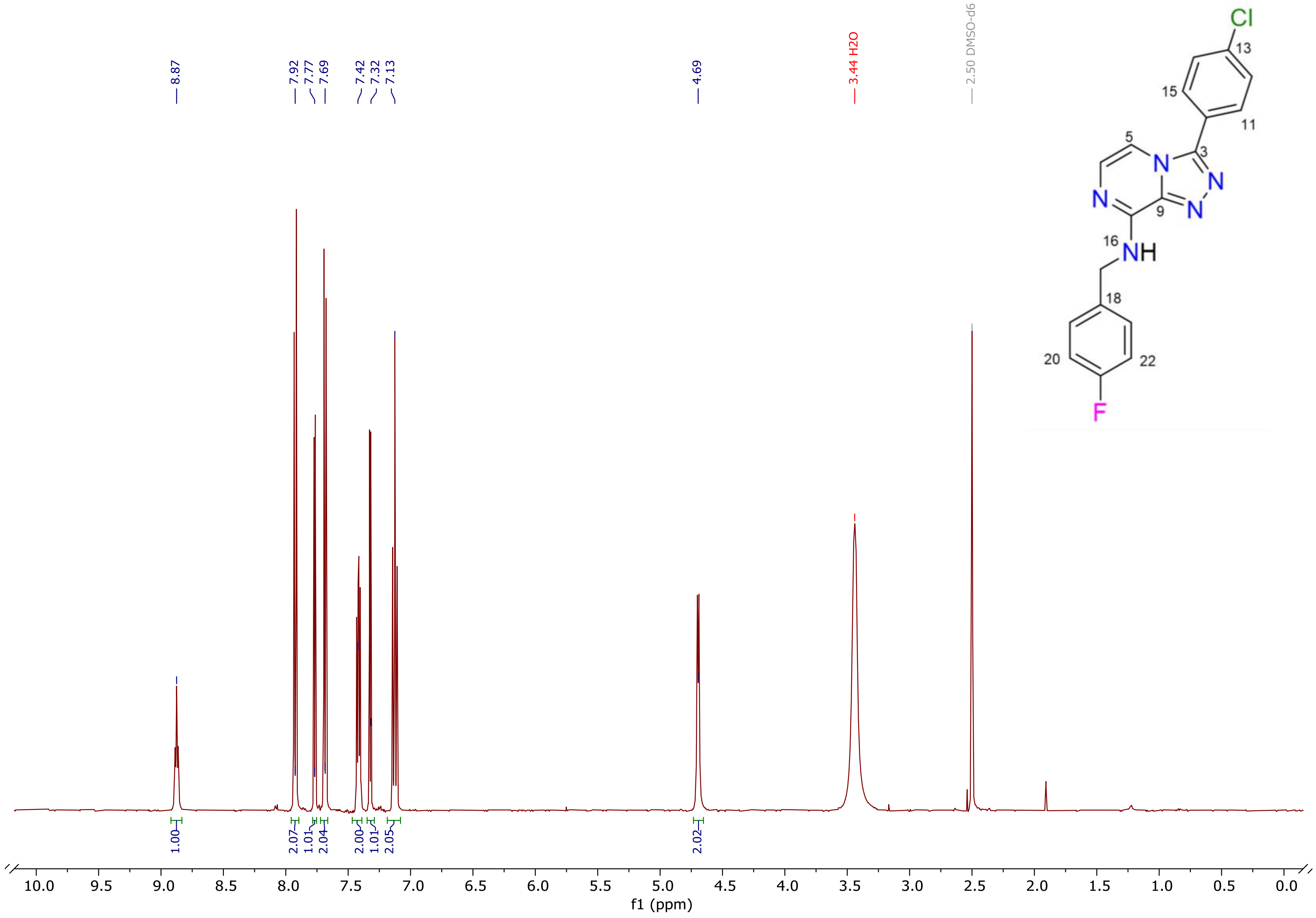

S14: ^{13}C NMR spectrum of compound **5** in $(CD_3)_2SO$

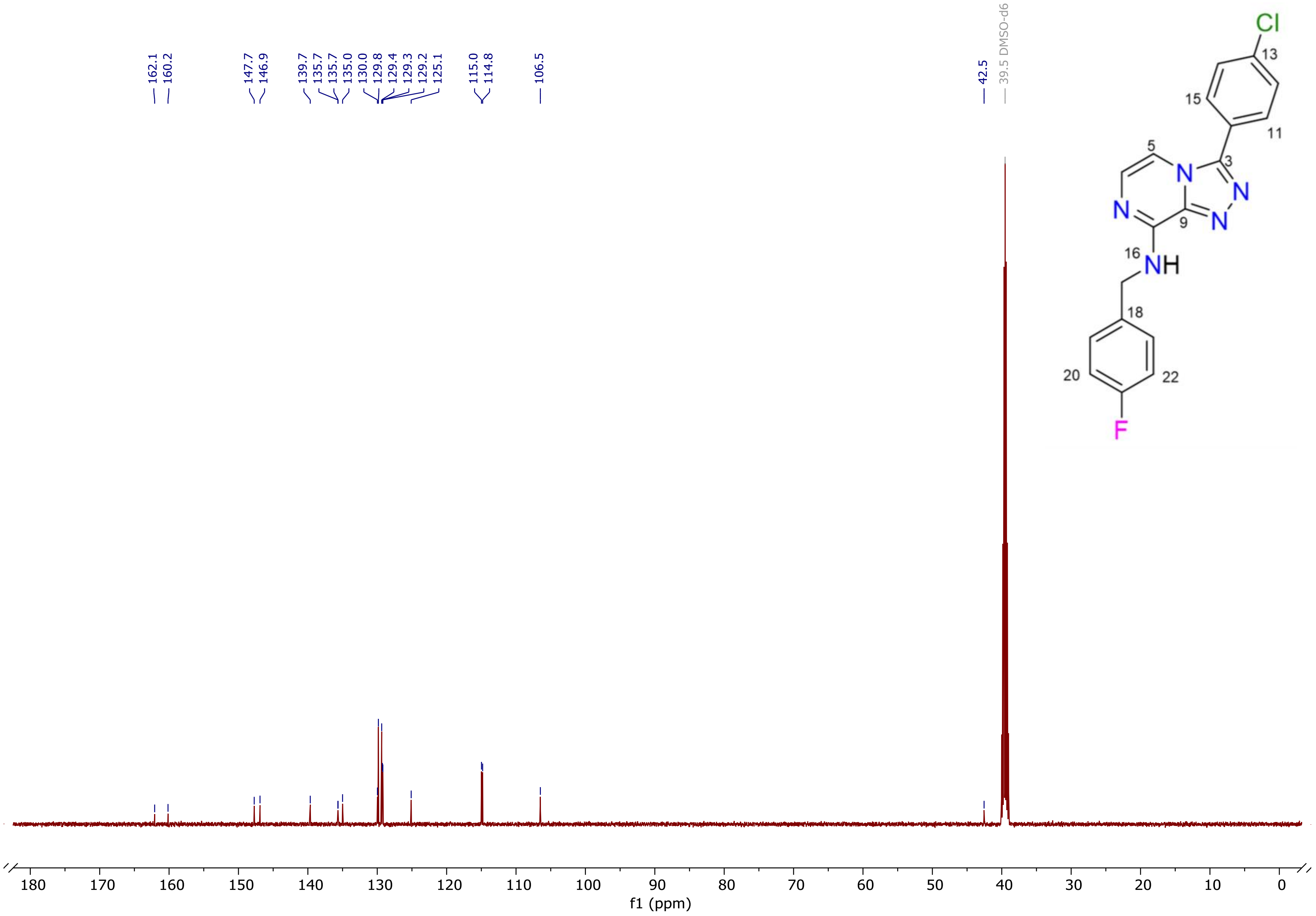

S15: NMR data table for compound **6**[a]

Position	δ_H, mult. (*J* in Hz), int.	δ_C, mult.	COSY	HMBC	ROESY
3		146.8, C			
5	7.73, d (4.9), 1H	105.8, CH	6	3, 6, 9	6, 11, 15
6	7.34, d (4.9), 1H	130.3, CH	5	5, 8	5
8		148.0, C			
9		139.7, C			
10		125.2, C			
11	7.92, m, 1H	129.8, CH	12	3, 13, 15	5, 12
12	7.69, m, 1H	129.4, CH	11	10, 14	11
13		134.9, C			
14	7.69, m, 1H	129.4, CH	15	10, 12	15
15	7.92, m, 1H	129.8, CH	14	3, 11, 13	5, 14
16	8.26, t (5.7), 1H		17	8, 9	17
17	3.46, m, 2H	41.8, CH_2	16, 18	8, 18, 19	16, 18, 19
18	1.65, m, 2H	21.8, CH_2	17, 19	17, 19	17, 19
19	0.92, t (7.4), 3H	11.4, CH_3	18	17, 18	18

[a] Recorded in $(CD_3)_2SO$ 500 MHz (^1H NMR) and 125 MHz (^{13}C NMR) at 25 °C.

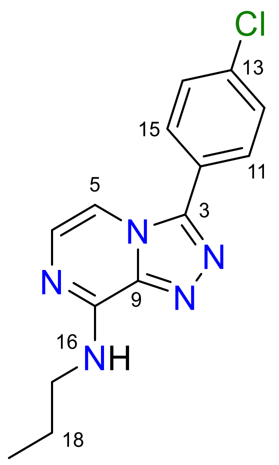

S16: ^{1}H NMR spectrum of compound **6** in $(CD_3)_2SO$

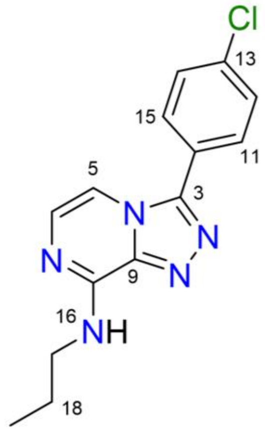

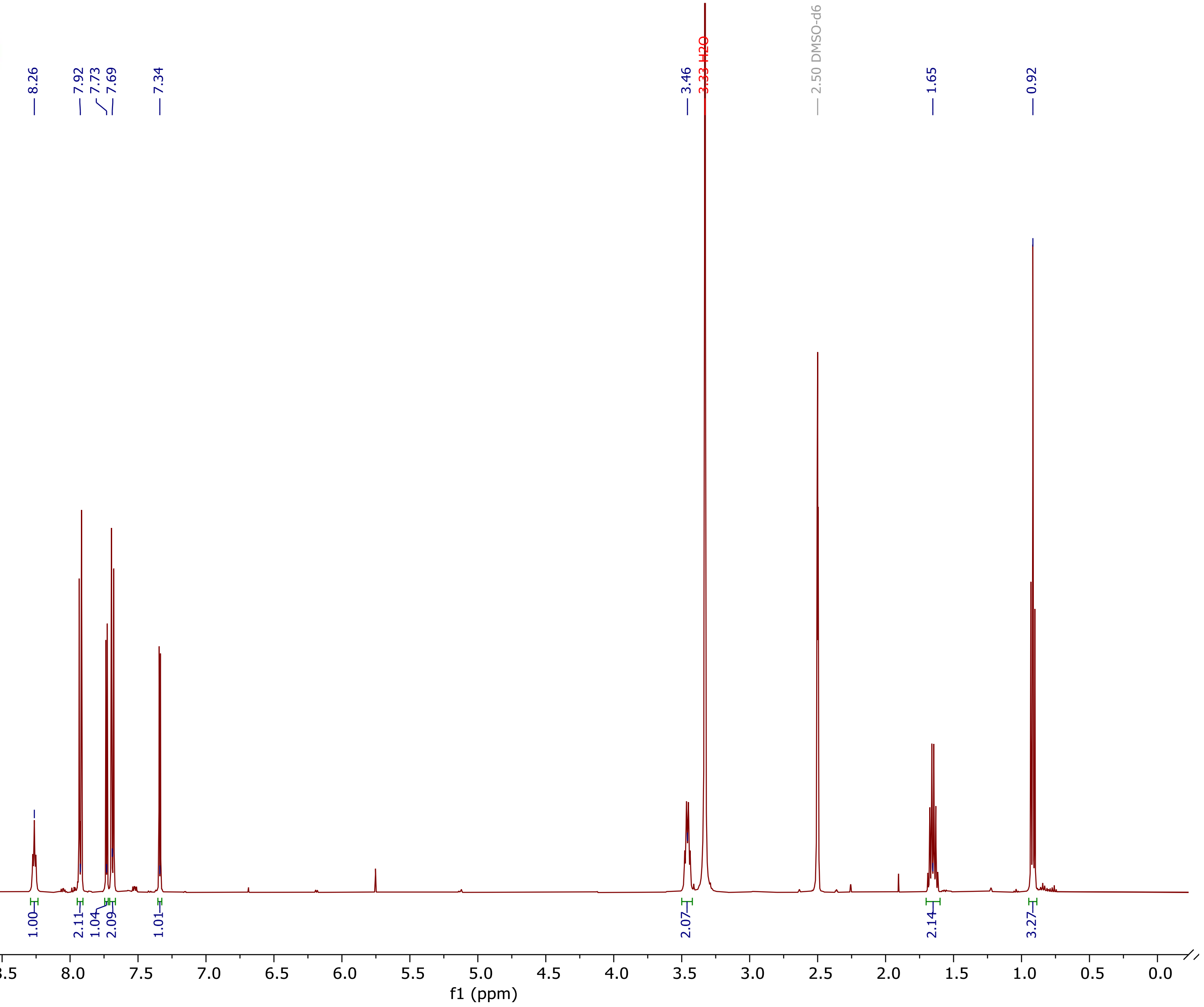

S17: ^{13}C NMR spectrum of compound **6** in $(CD_3)_2SO$

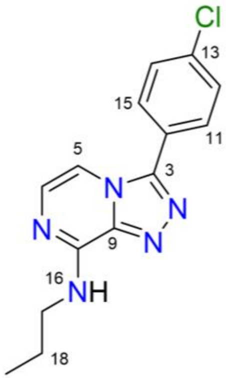

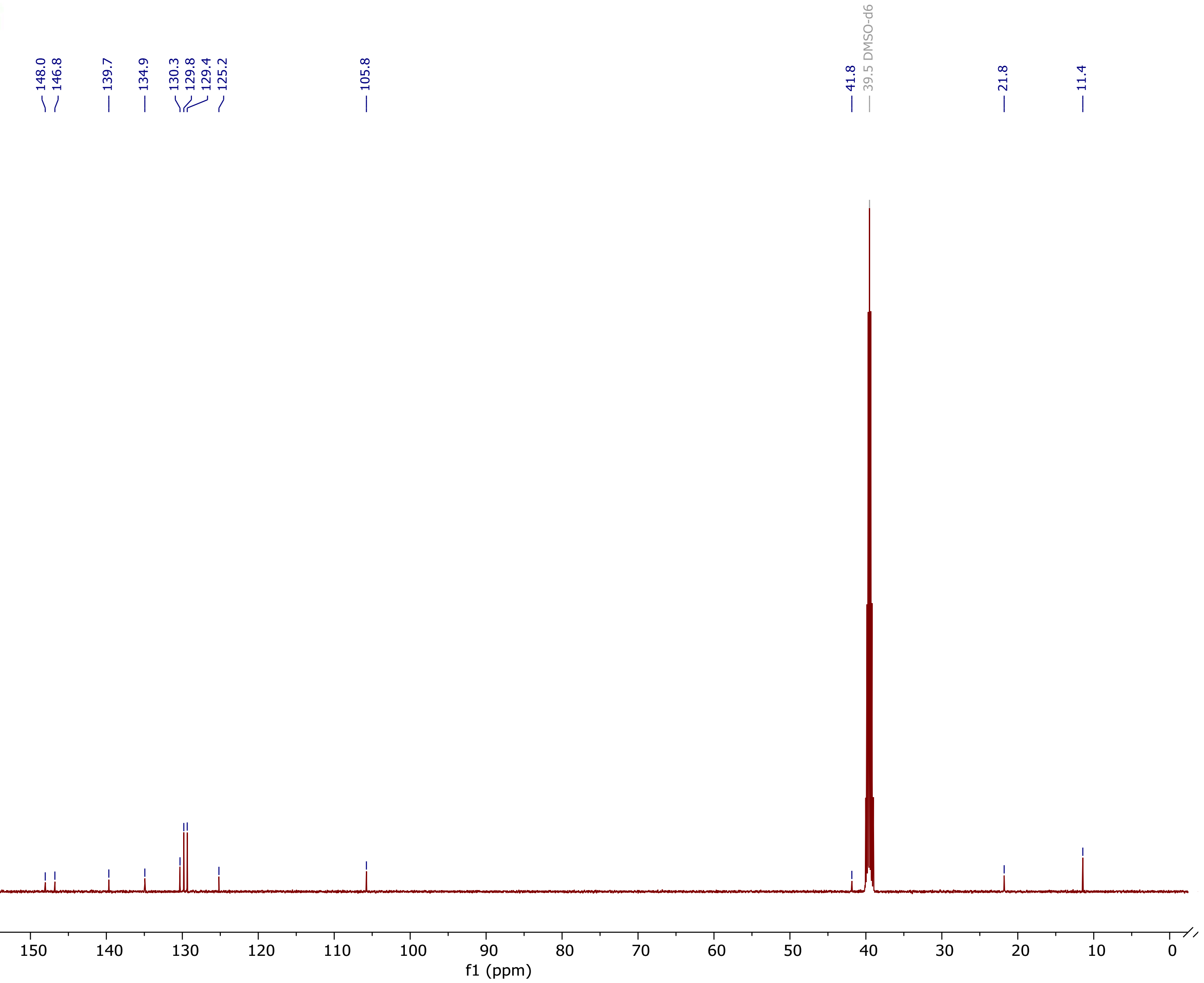

S18: NMR data table for compound **7**[a]

Position	δH, mult. (J in Hz), int.	δC, mult.	COSY	HMBC	ROESY
3		146.8, C			
5	7.73, d (4.8), 1H	105.7, CH	6	6, 9	6
6	7.35, d (4.8), 1H	130.3, CH	5	5, 8	5
8		148.0, C			
9		139.7, C			
10		125.2, C			
11	7.93, m, 1H	129.8, CH	12	3, 13, 15	12
12	7.69, m, 1H	129.3, CH	11	10, 14	11
13		134.9, C			
14	7.69, m, 1H	129.3, CH	15	10, 12	15
15	7.93, m, 1H	129.8, CH	14	3, 11, 13	14
16	8.22, brt (5.7), 1H		17		17
17	3.52, m, 2H	38.4, CH_2	16, 18		16, 18, 20, 21
18	1.54, m, 2H	37.5, CH_2	17, 19	20, 21	17, 20, 21
19	1.65, m, 1H	25.4, CH	18, 20, 21	20, 21	17[w], 20, 21
20	0.92, d (6.6), 3H	22.5, CH_3	19	18, 19, 21	17, 18, 19
21	0.92, d (6.6), 3H	22.5, CH_3	19	18, 19, 20	17, 18, 19

[a] Recorded in $(CD_3)_2SO$ 500 MHz (^1H NMR) and 125 MHz (^{13}C NMR) at 25 °C; [w] Weak.

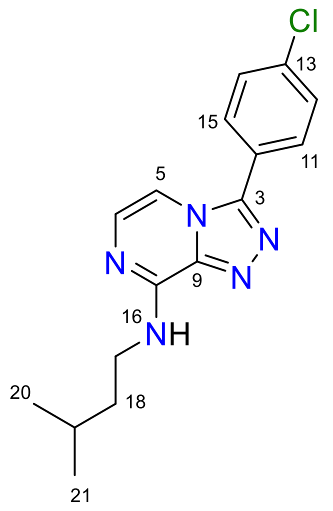

S19: ^{1}H NMR spectrum of compound **7** in $(CD_3)_2SO$

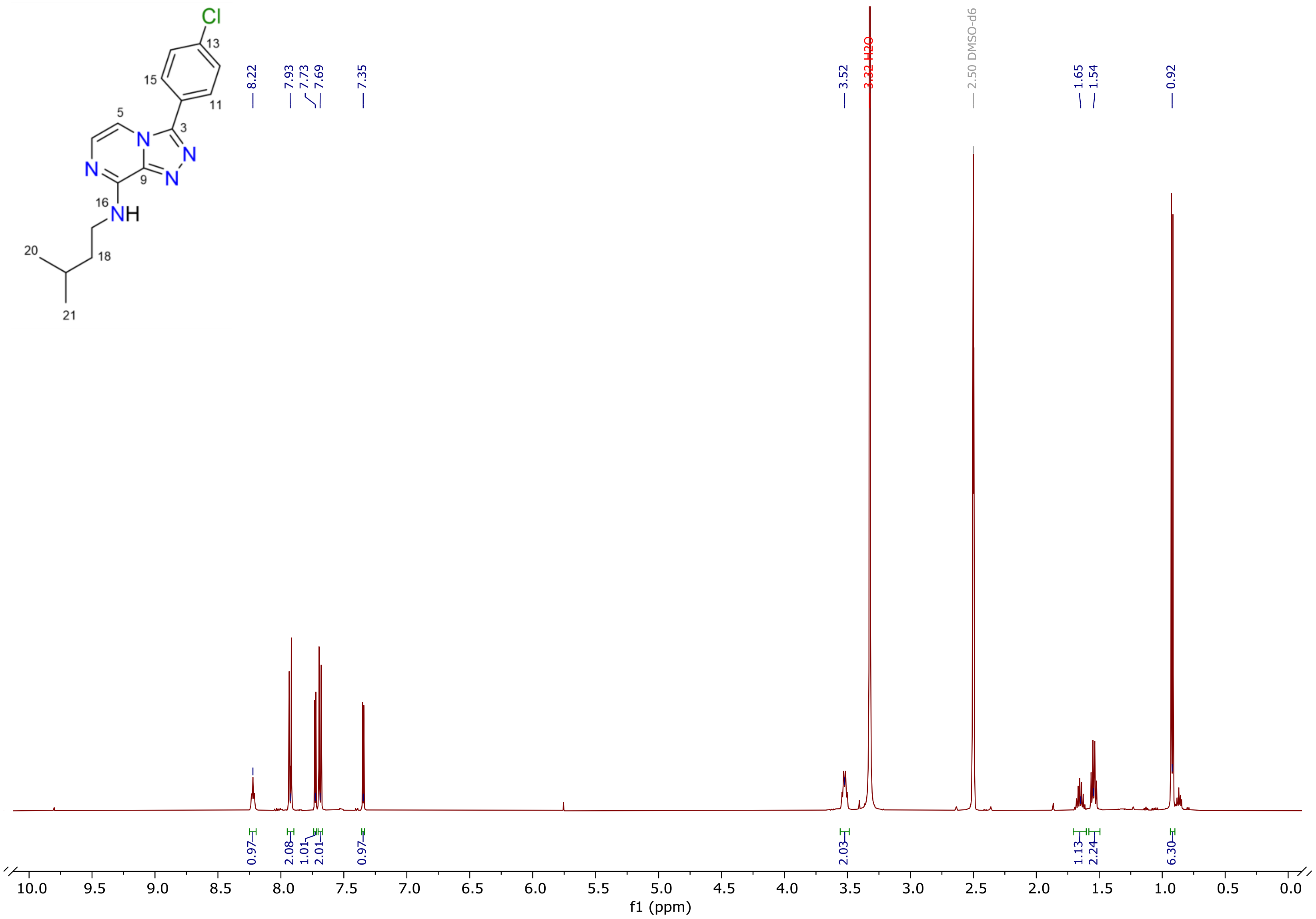

S20: ^{13}C NMR spectrum of compound **7** in $(CD_3)_2SO$

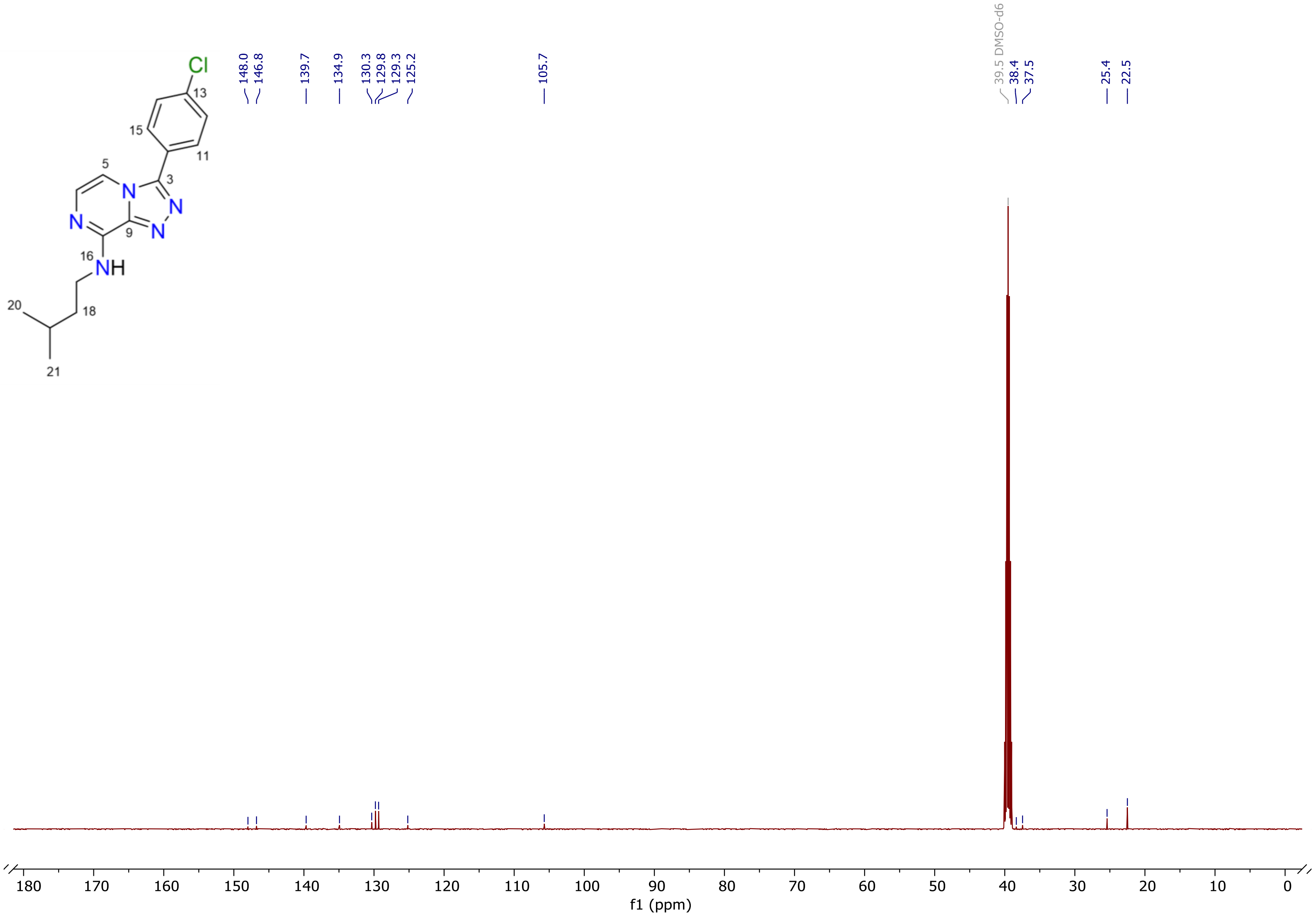

S21: NMR data table for compound **8**[a]

Position	δ_H, mult. (*J* in Hz), int.	δ_C, mult.	COSY	HMBC	ROESY
3		146.8, C			
5	7.72, d (4.8), 1H	105.7, CH	6	3, 6, 9	6, 11, 15
6	7.34, d (4.8), 1H	130.3, CH	5	5, 8	5
8		147.2, C			
9		139.6, C			
10		125.2, C			
11	7.92, m, 1H	129.8, CH	12	3, 13, 15	12
12	7.68, m, 1H	129.3, CH	11	10, 14	11
13		134.9, C			
14	7.68, m, 1H	129.3, CH	15	10, 12	15
15	7.92, m, 1H	129.8, CH	14	3, 11, 13	14
16	7.98, d (8.2), 1H		17	9, 18[w], 22[w]	17, 18, 22
17	4.07, m, 1H	49.0, CH	16, 18, 22		16, 18, 22
18	1.46, m, 1H	31.9, CH_2	17, 19	19, 20, 21	19
	1.91, m, 1H	31.9, CH_2	17, 19	19	19
19	1.33, m, 1H	25.0, CH_2	18, 20	18, 21, 22	18, 20
	1.76, m, 1H	25.0, CH_2	18, 20	18, 20	18, 20
20	1.63, m, 1H	25.3, CH_2	19, 21	18, 19, 21, 22	19, 21
	1.16, m 1H	25.3, CH_2	19, 21	22	19
21	1.33, m, 1H	25.0, CH_2	18, 20	18, 19	18, 20
	1.76, m, 1H	25.0, CH_2	18, 20	18, 20	18, 20
22	1.46, m, 1H	31.9, CH_2	17, 23	20	23
	1.91, m, 1H	31.9, CH_2	17, 23	19	23

[a] Recorded in $(CD_3)_2SO$ 500 MHz (^{1}H NMR) and 125 MHz (^{13}C NMR) at 25 °C; [w] Weak.

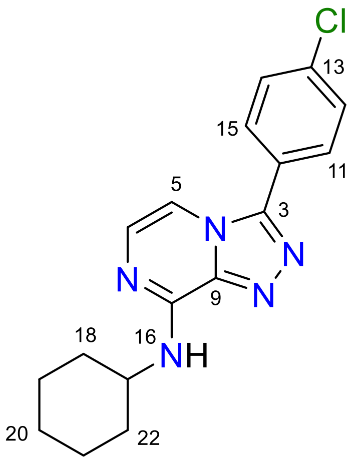

S22: ^{1}H NMR spectrum of compound **8** in $(CD_3)_2SO$

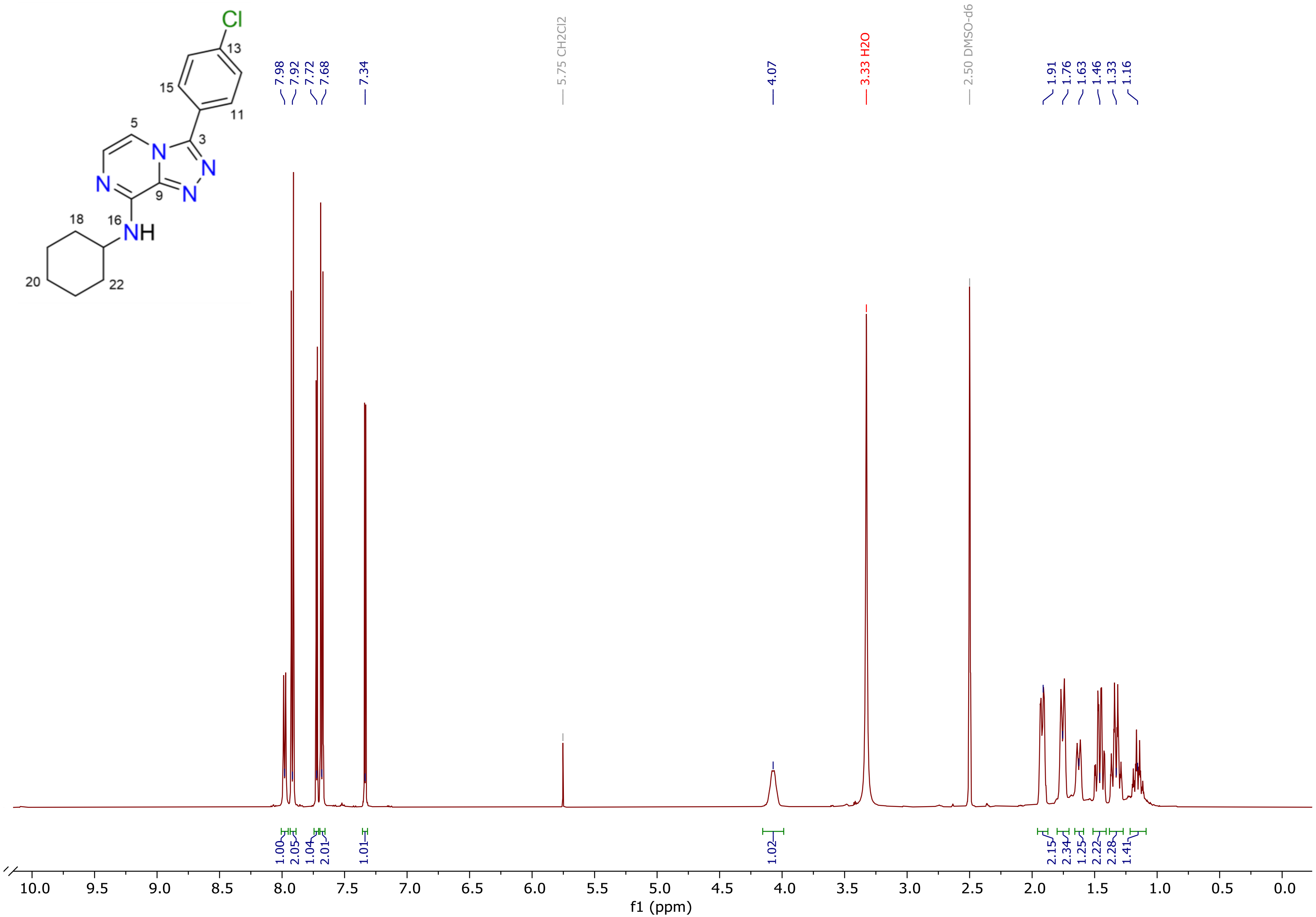

S23: ^{13}C NMR spectrum of compound **8** in $(CD_3)_2SO$

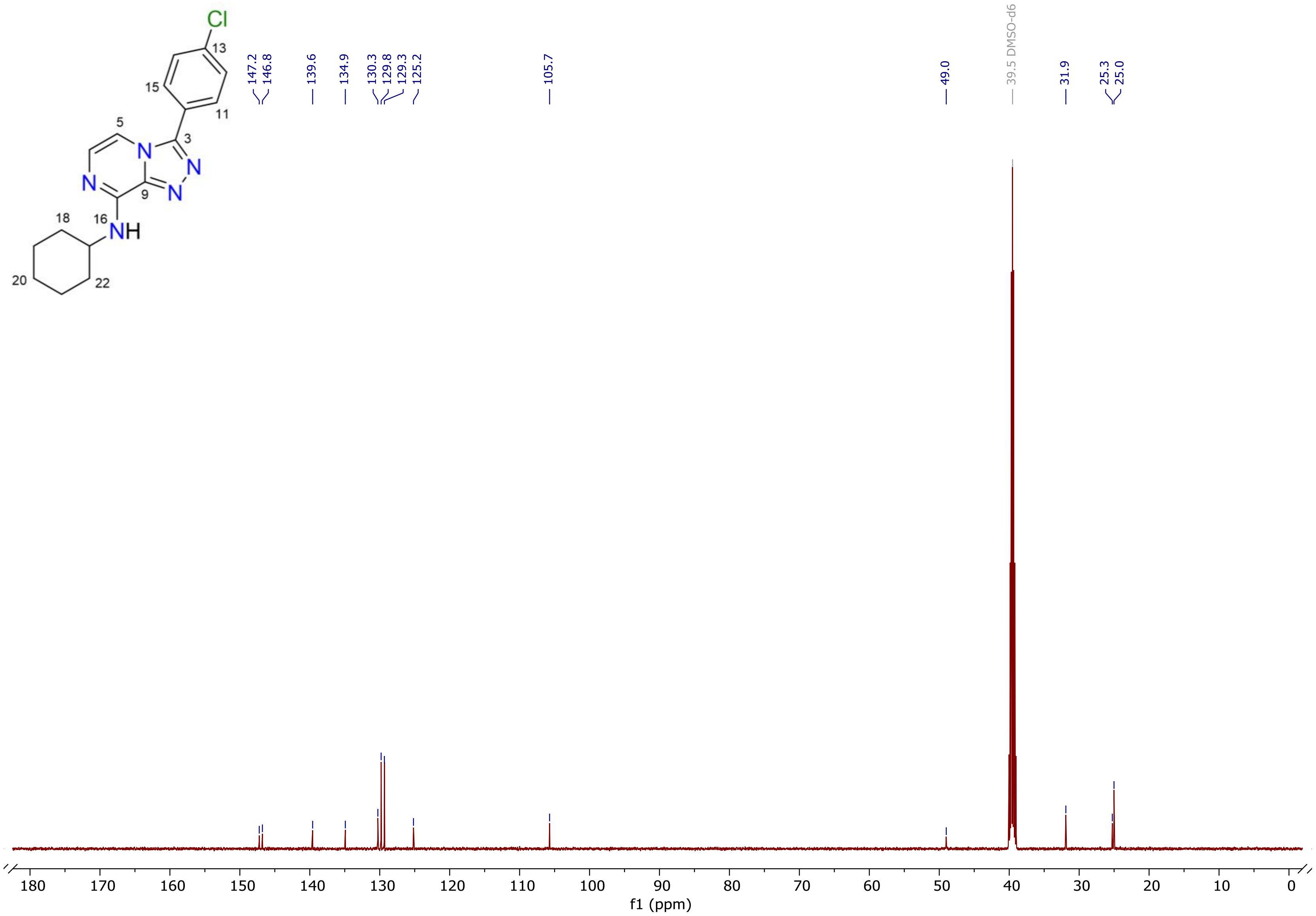

S24: NMR data table for compound **9**[a]

Position	δ_H, mult. (*J* in Hz), int.	δ_C, mult.	COSY	HMBC	ROESY
3		146.8, C			
5	7.76, d (4.8), 1H	106.2, CH	6	3, 6, 9	6
6	7.35, d (4.8), 1H	130.2, CH	5	5, 8	5
8		148.0, C			
9		139.7, C			
10		125.1, C			
11	7.92, m, 1H	129.8, CH	12	3, 13, 15	12
12	7.69, m, 1H	129.4, CH	11	10, 14	11
13		135.0, C			
14	7.69, m, 1H	129.4, CH	15	10, 12	15
15	7.92, m, 1H	129.8, CH	14	3, 11, 13	14
16	8.13, t (5.8), 1H		17		17
17	3.68, dt (5.8, 6.2), 2H	39.5, CH_2	16, 18	18[w]	
18	3.57, t (6.2), 2H	69.8, CH_2	17	17, 20[w]	
20	3.28, s, 3H	57.9, CH_3		18	

[a] Recorded in $(CD_3)_2SO$ 500 MHz (^1H NMR) and 125 MHz (^{13}C NMR) at 25 °C; [w] Weak.

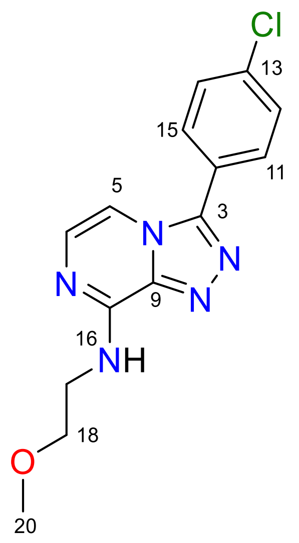

S25: ^{1}H NMR spectrum of compound **9** in $(CD_3)_2SO$

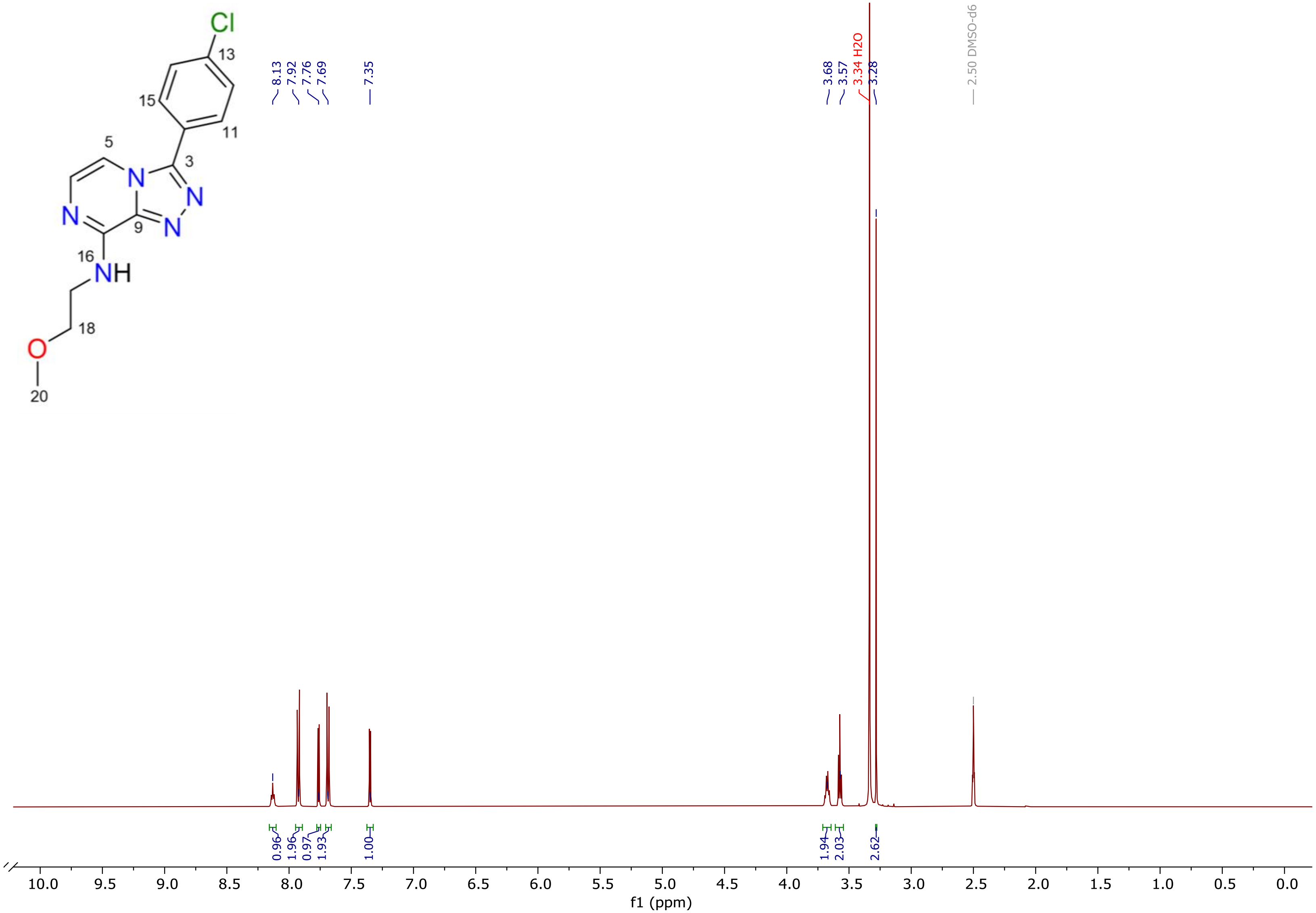

S26: ^{13}C NMR spectrum of compound **9** in $(CD_3)_2SO$

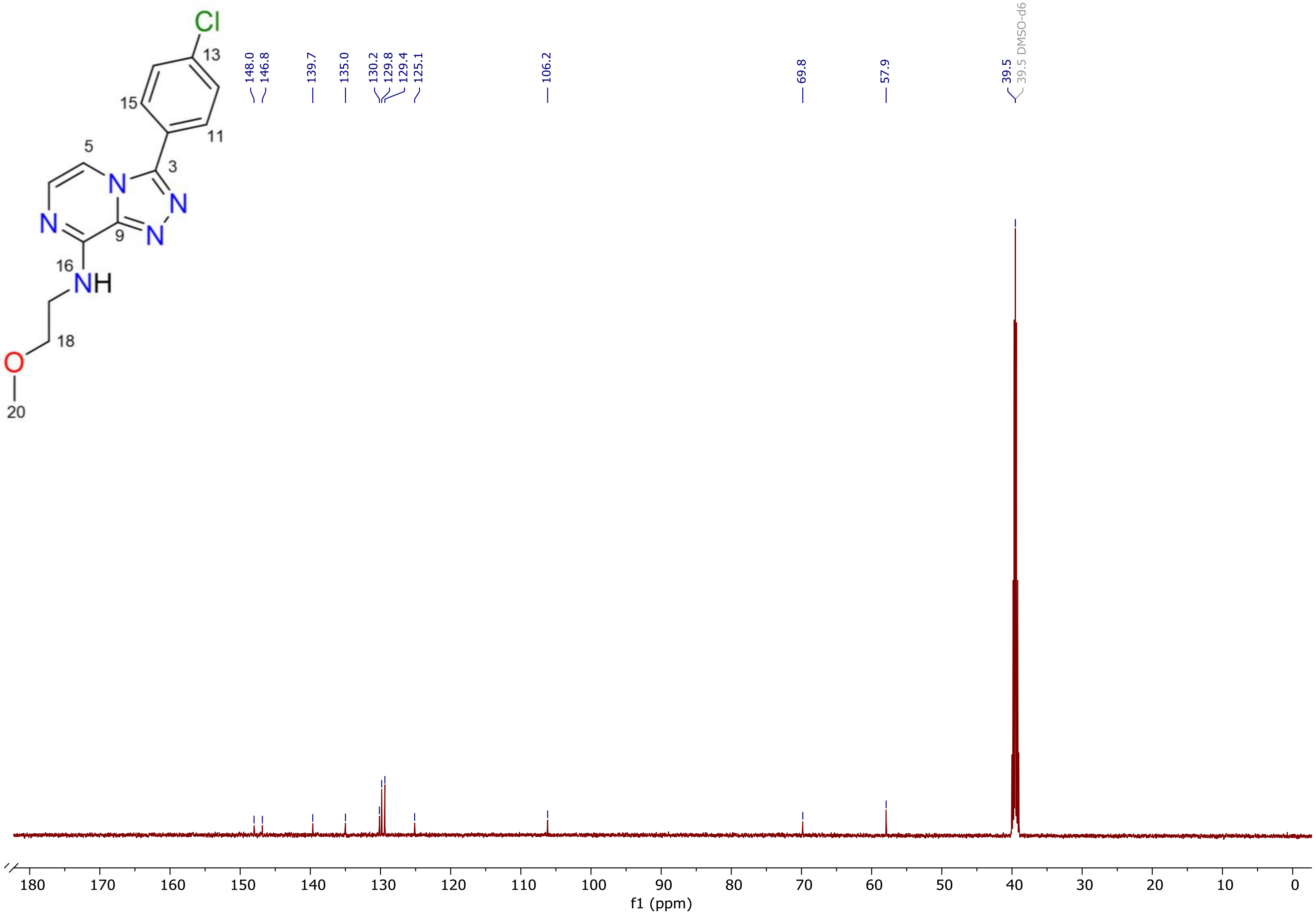

S27: NMR data table for compound **10**[a]

Position	δ_H, mult. (*J* in Hz), int.	δ_C, mult.	COSY	HMBC	ROESY
3		146.8, C			
5	7.74, d (4.8), 1H	106.0, CH	6	3, 6, 9	6, 11, 15
6	7.34, d (4.8), 1H	130.3, CH	5	5, 8	5
8		148.0, C			
9		139.7, C			
10		125.1, C			
11	7.91, m, 1H	129.8, CH	12	3, 13, 15	5, 12
12	7.67, m, 1H	129.4, CH	11	10, 14	11
13		135.0, C			
14	7.67, m, 1H	129.4, CH	15	10, 12	15
15	7.91, m, 1H	129.8, CH	14	3, 11, 13	5, 12
16	7.95, t (5.5), 1H		17	8, 17[w]	17
17	3.60, dt (5.5, 6.6), 2H	37.9, CH_2	16, 18	8, 18	16, 18, 20, 21
18	2.55, t (6.6), 2H	57.4, CH_2	17	17, 20, 21	17, 20, 21
20	2.22, s, 3H	45.1, CH_3		18, 21	17, 18
21	2.22, s, 3H	45.1, CH_3		18, 20	17, 18

[a] Recorded in $(CD_3)_2SO$ 500 MHz (^1H NMR) and 125 MHz (^{13}C NMR) at 25 °C; [w] Weak.

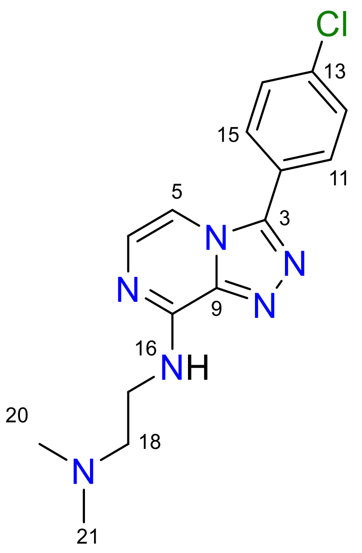

S28: ^{1}H NMR spectrum of compound **10** in $(CD_3)_2SO$

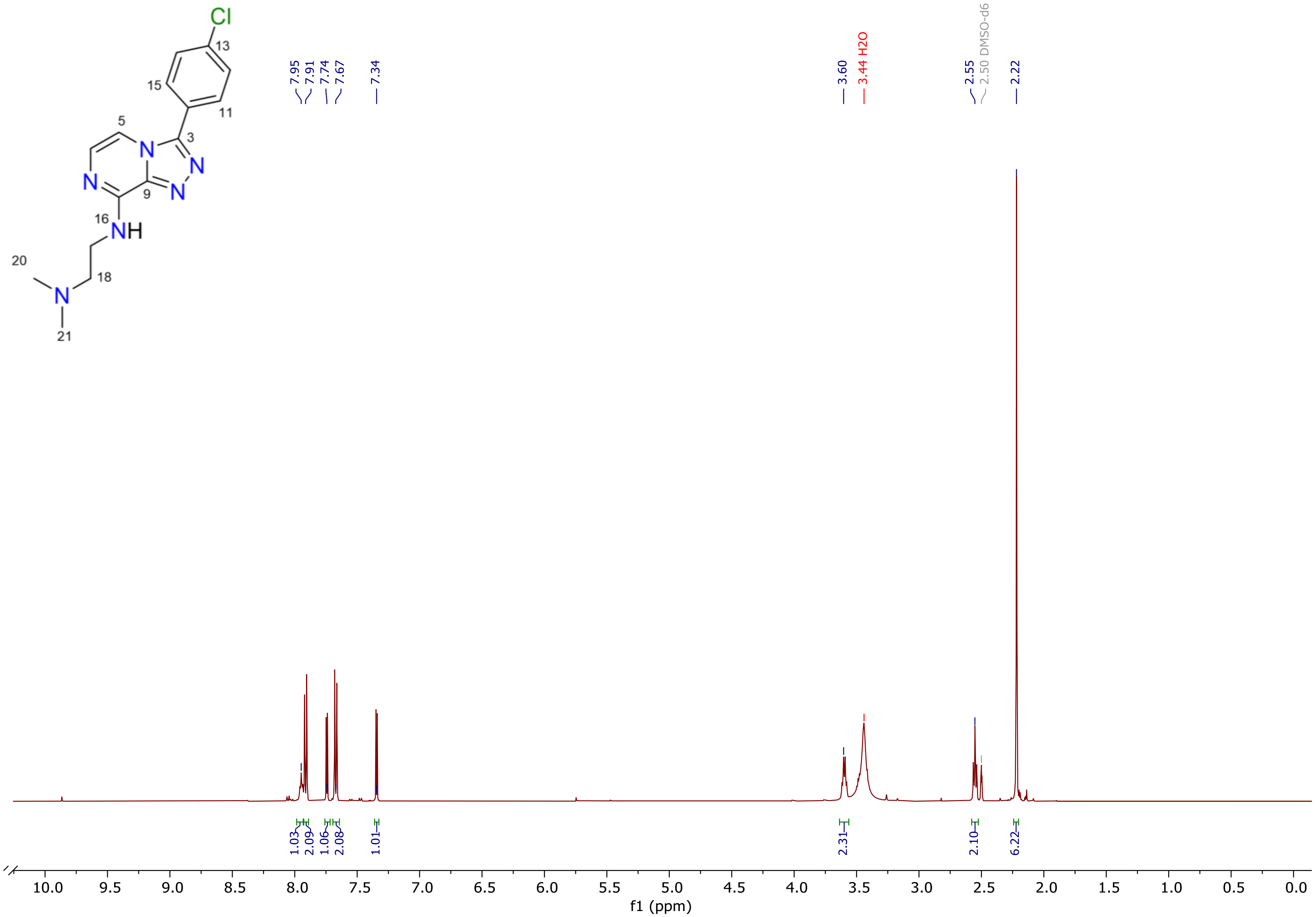

S29: ^{13}C NMR spectrum of compound **10** in $(CD_3)_2SO$

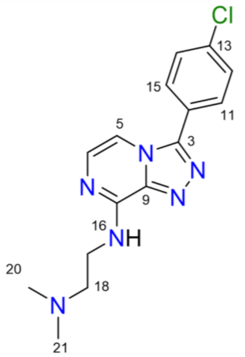

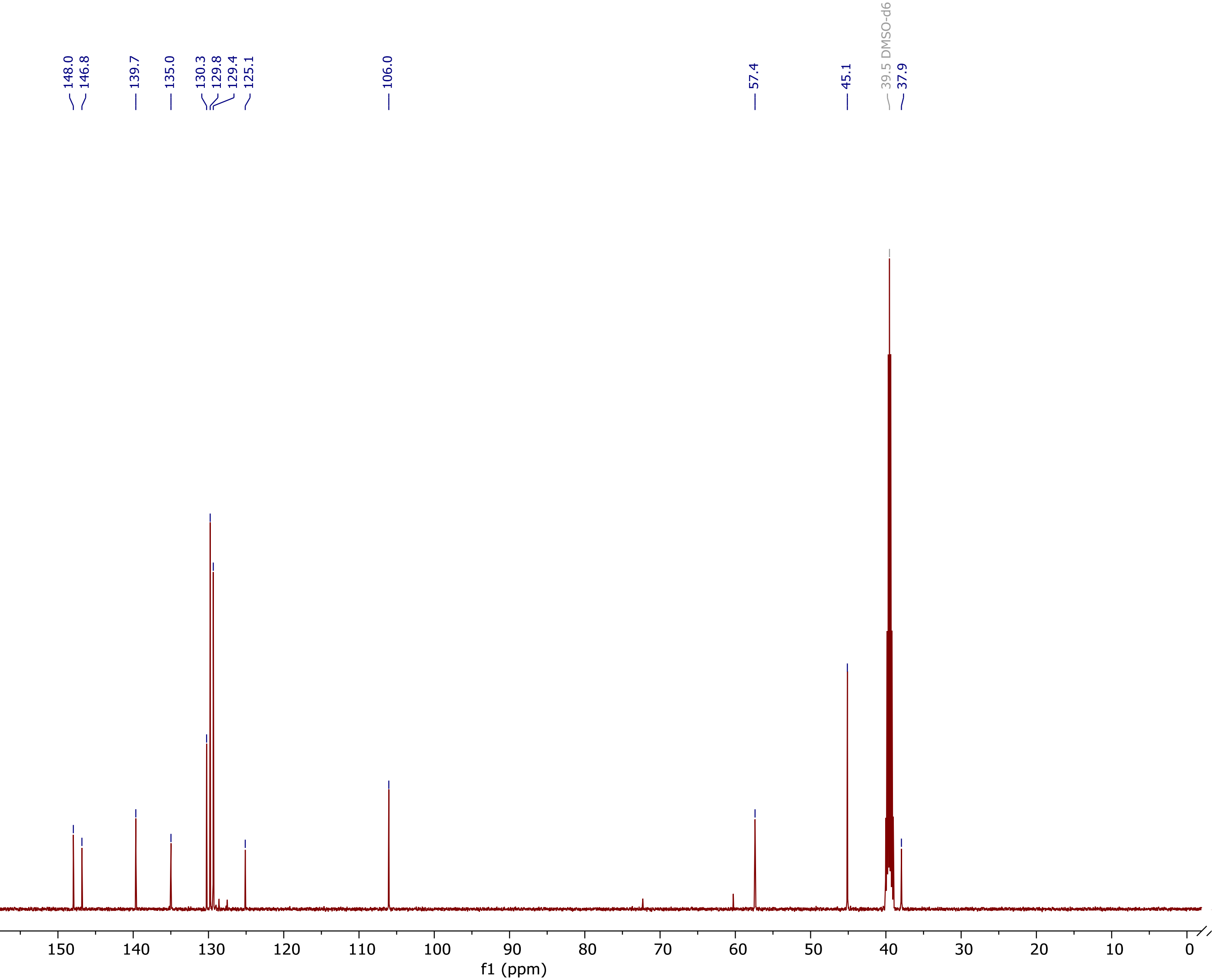

S30: NMR data table for compound **11**[a]

Position	δ_H, mult. (*J* in Hz), int.	δ_C, mult.	COSY	HMBC	ROESY
3		146.8, C			
5	7.75, d (4.8), 1H	106.0, CH	6	6, 9	6, 11, 15
6	7.35, d (4.8), 1H	130.3, CH	5	5, 8	5
8		148.0, C			
9		139.7, C			
10		125.1, C			
11	7.92, m, 1H	129.8, CH	12	3, 13, 15	12
12	7.68, m, 1H	129.4, CH	11	10, 14	11
13		135.0, C			
14	7.68, m, 1H	129.4, CH	15	10, 12	15
15	7.92, m, 1H	129.8, CH	14	3, 11, 13	14
16	7.94, t (5.8), 1H		17	8, 17 [w]	17, 18
17	3.57, dt (5.8, 7.1), 2H	38.0, CH_2	16, 18	8, 18	16, 18, 20, 22
18	2.69, t (7.1), 2H	50.8, CH_2	17	17, 20, 22	16, 17, 20, 21, 22, 23
20	2.55, q (7.1), 2H	46.6, CH_2	21	18, 21, 22	17, 18, 21
21	0.98, t (7.1), 3H	11.9, CH_3	20	20	18, 20
22	2.55, q (7.1), 2H	46.6, CH_2	23	18, 20, 23	17, 18, 23
23	0.98, t (7.1), 3H	11.9, CH_3	22	22	18, 22

[a] Recorded in $(CD_3)_2SO$ 500 MHz (^1H NMR) and 125 MHz (^{13}C NMR) at 25 °C; [w] Weak.

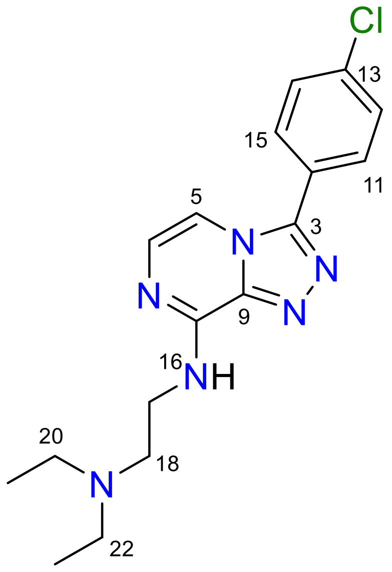

S31: ^{1}H NMR spectrum of compound **11** in $(CD_3)_2SO$

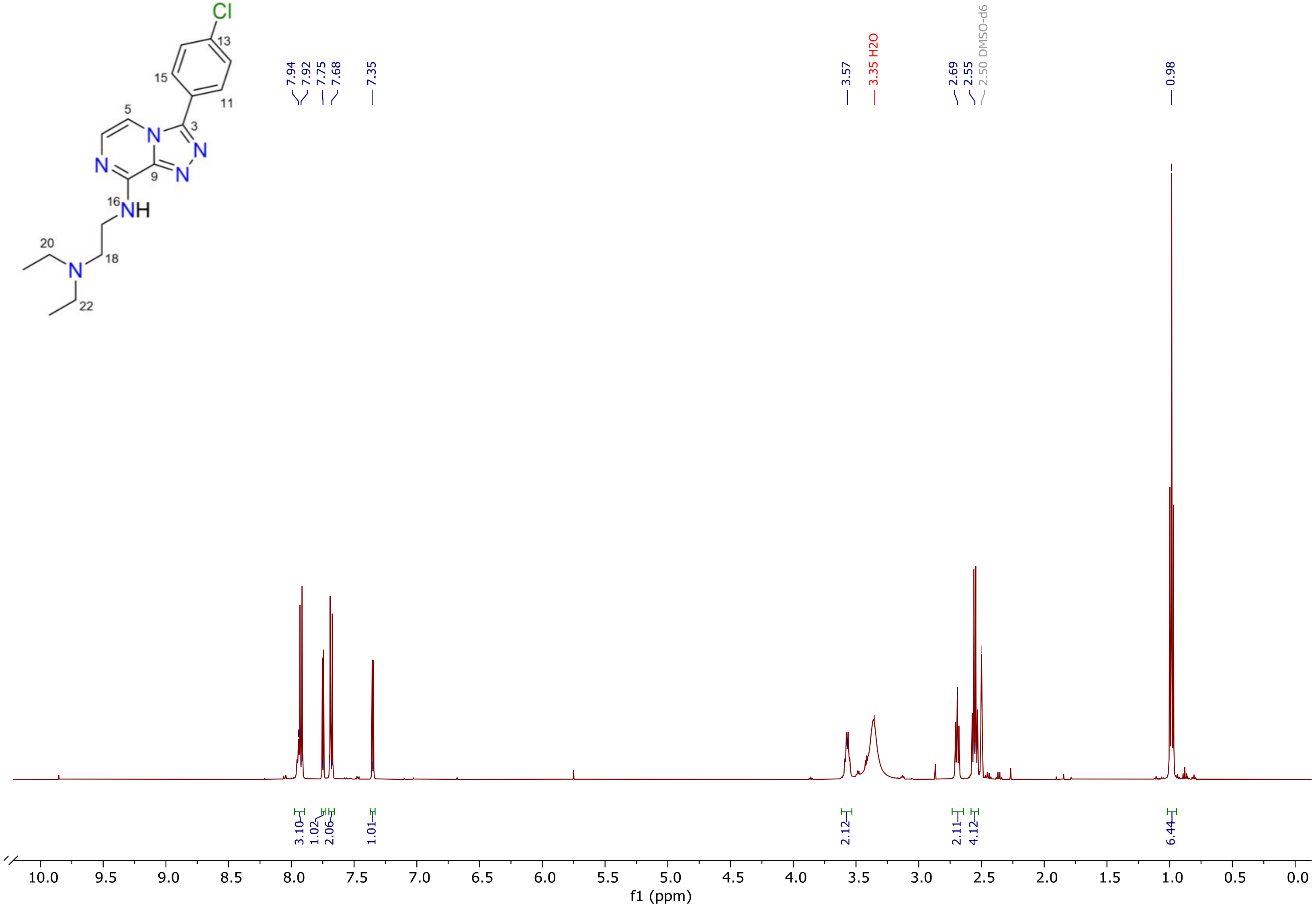

S32: ^{13}C NMR spectrum of compound **11** in $(CD_3)_2SO$

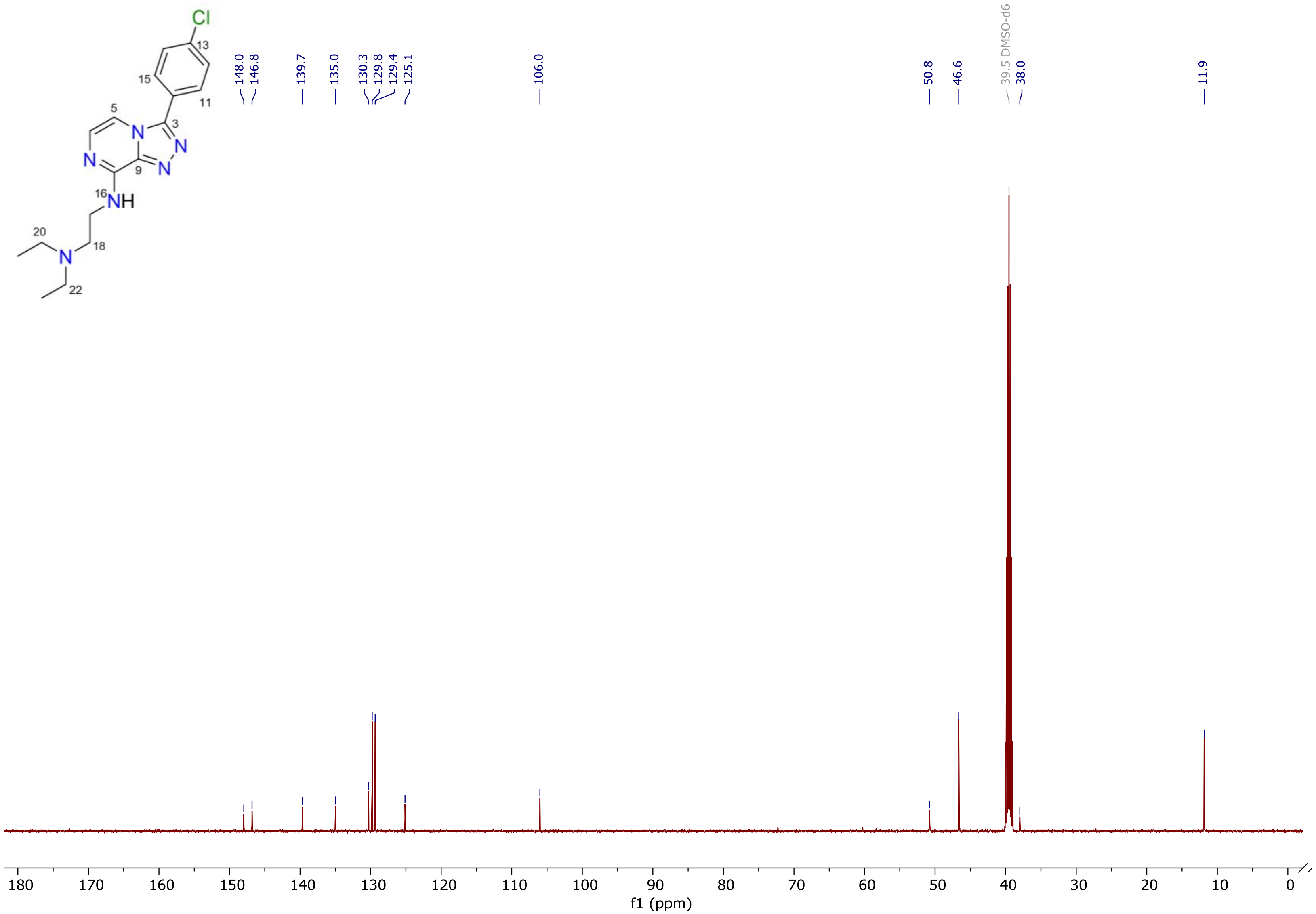

S33: NMR data table for compound **12**[a]

Position	δ_H, mult. (*J* in Hz), int.	δ_C, mult.	COSY	HMBC	ROESY
3		146.8, C			
5	7.75, d (4.8), 1H	105.9, CH	6	3, 6, 9	6, 11, 15
6	7.35, d (4.8), 1H	130.3, CH	5	5, 8	5
8		148.0, C			
9		139.7, C			
10		125.2, C			
11	7.92, m, 1H	129.8, CH	12	3, 13, 15	12
12	7.69, m, 1H	129.4, CH	11	10, 14	11
13		135.0, C			
14	7.69, m, 1H	129.4, CH	15	10, 12	15
15	7.92, m, 1H	129.8, CH	14	3, 11, 13	14
16	8.34, brt (5.5), 1H		17		17
17	3.54, m, 2H	38.4, CH_2	16, 18	8, 18[w]	16, 18
18	1.83, m, 2H	25.7, CH_2	17, 19	17, 19	17, 19, 21, 22
19	2.49, t (7.1), 2H	56.5, CH_2	18	17, 18[w], 21, 22	18, 21, 22
21	2.29, s, 3H	44.5, CH_3		19, 22	18, 19
22	2.29, s, 3H	44.5, CH_3		19, 21	18, 19

[a] Recorded in $(CD_3)_2SO$ 500 MHz (^{1}H NMR) and 125 MHz (^{13}C NMR) at 25 °C; [w] Weak.

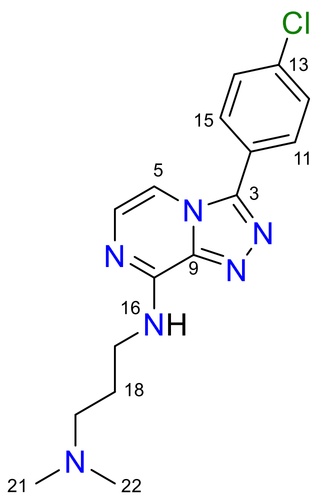

S34: ^{1}H NMR spectrum of compound **12** in $(CD_3)_2SO$

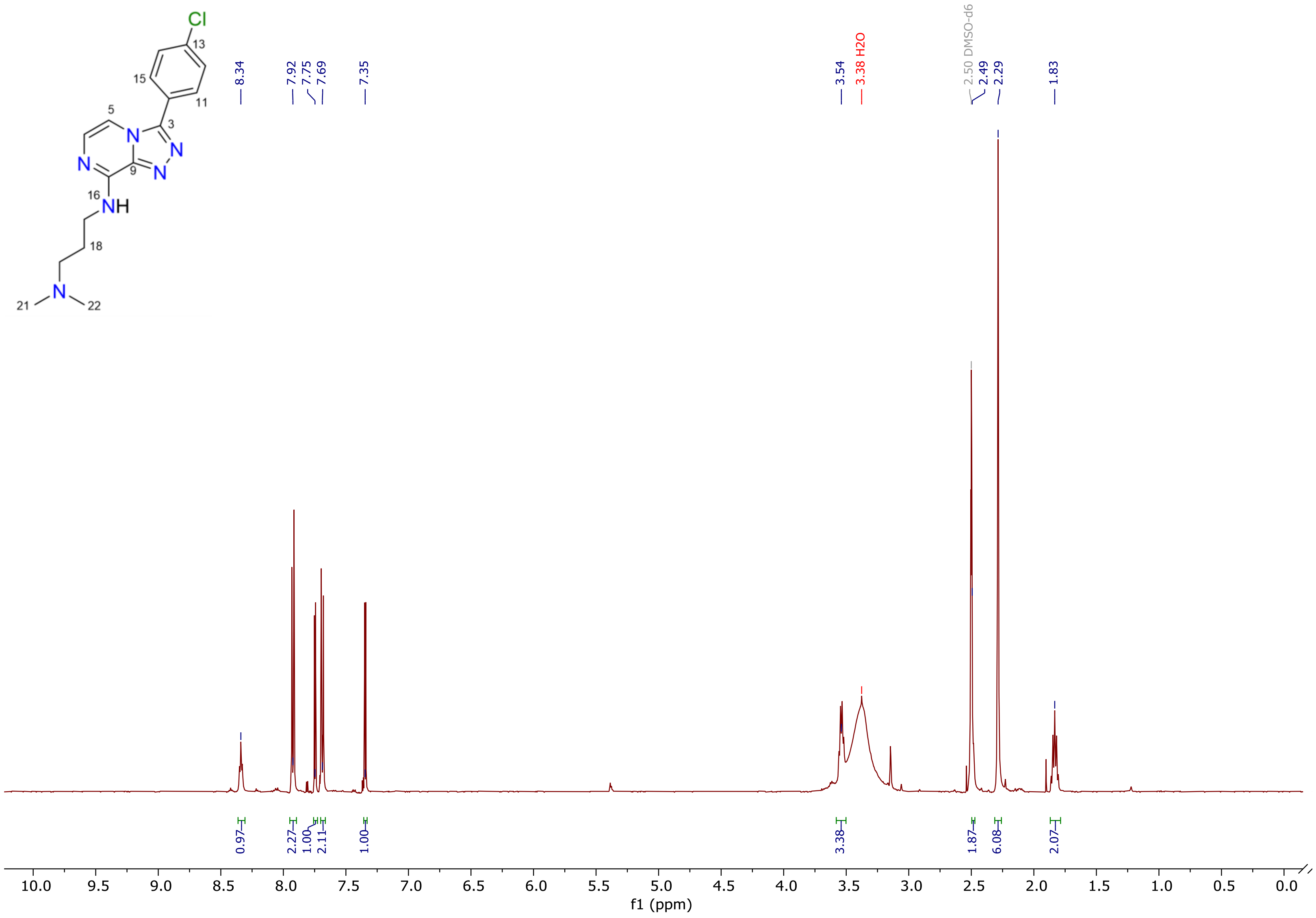

S35: ^{13}C NMR spectrum of compound **12** in $(CD_3)_2SO$

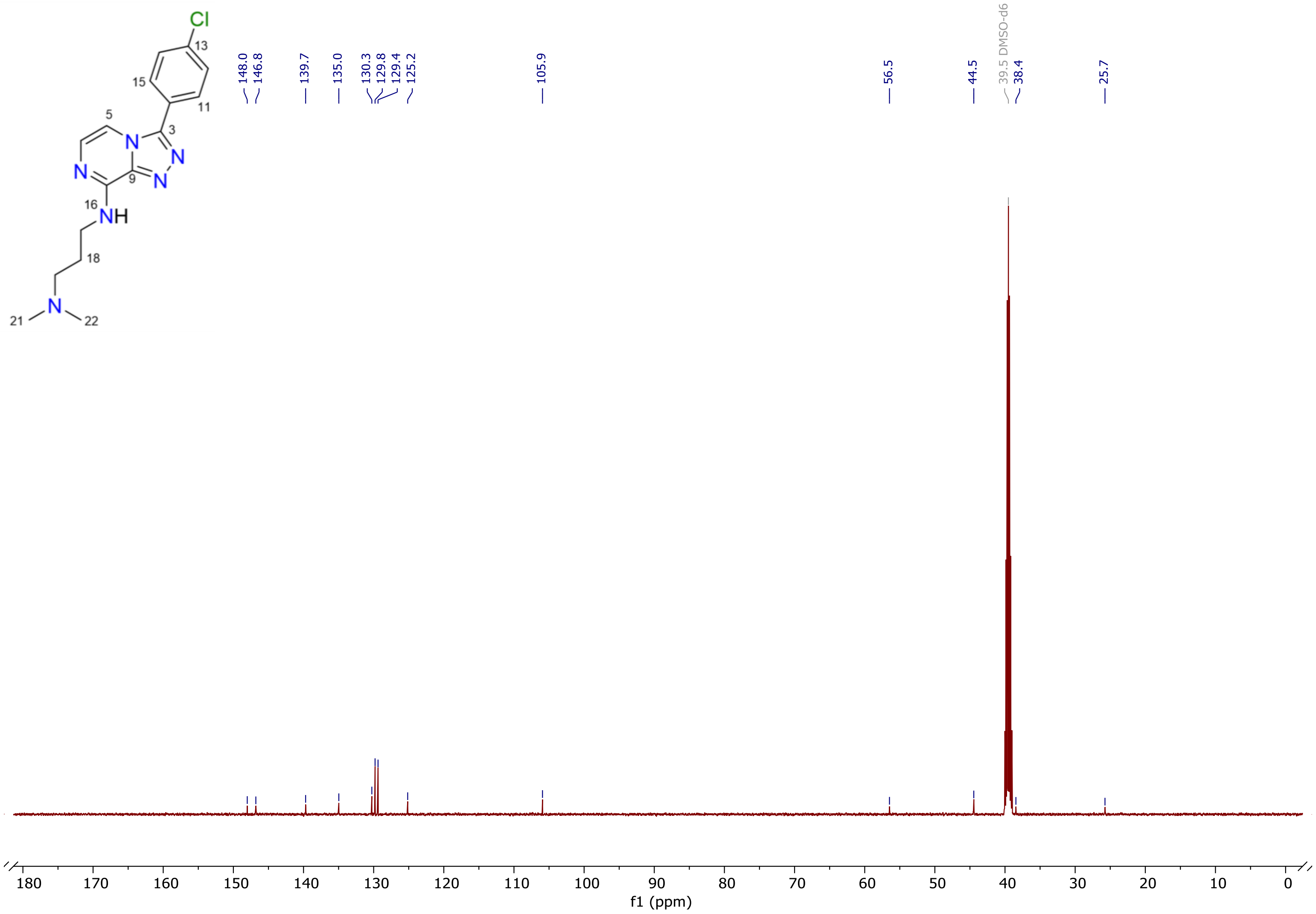

S36: NMR data table for compound **13**[a]

Position	δ_H, mult. (*J* in Hz), int.	δ_C, mult.	COSY	HMBC	ROESY
3		146.7, C			
5	7.73, d (4.8), 1H	105.9, CH	6	3, 6, 9	6, 11, 15
6	7.33, d (4.8), 1H	130.3, CH	5	5, 8	5
8		148.0, C			
9		139.7, C			
10		125.1, C			
11	7.91, m, 1H	129.7, CH	12	3, 13, 15	5, 12
12	7.66, m, 1H	129.3, CH	11	10,14	11
13		134.9, C			
14	7.66, m, 1H	129.3, CH	15	10, 12	15
15	7.91, m, 1H	129.7, CH	14	3, 11, 13	5, 14
16	8.44, t (5.3), 1H		17	8, 9	17
17	3.55, m, 2H	38.9, CH_2	16, 18	8, 18, 19	16, 18, 19
18	1.84, m, 2H	24.8, CH_2	17, 19	17, 19	17, 19, 21, 23
19	2.65, t (8.0), 2H	50.1, CH_2	18	17 [w], 18, 21, 23	17, 18
21	2.62, q (7.2), 2H	46.2, CH_2	22	19, 22, 23	22
22	1.03, t (7.2), 3H	10.7, CH_3	21	21	21
23	2.63, q (7.2), 2H	46.2, CH_2	24	19, 21, 24	24
24	1.03, t (7.2), 3H	10.7, CH_3	23	23	23

[a] Recorded in $(CD_3)_2SO$ 500 MHz (^1H NMR) and 125 MHz (^{13}C NMR) at 25 °C; [w] Weak.

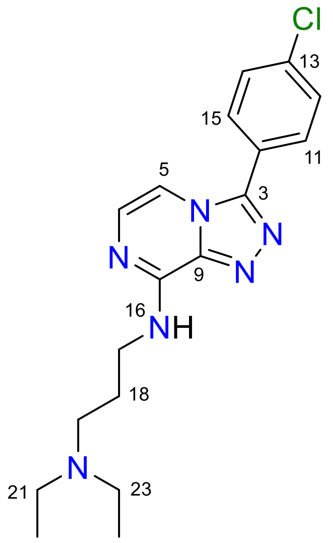

S37: ^{1}H NMR spectrum of compound **13** in $(CD_3)_2SO$

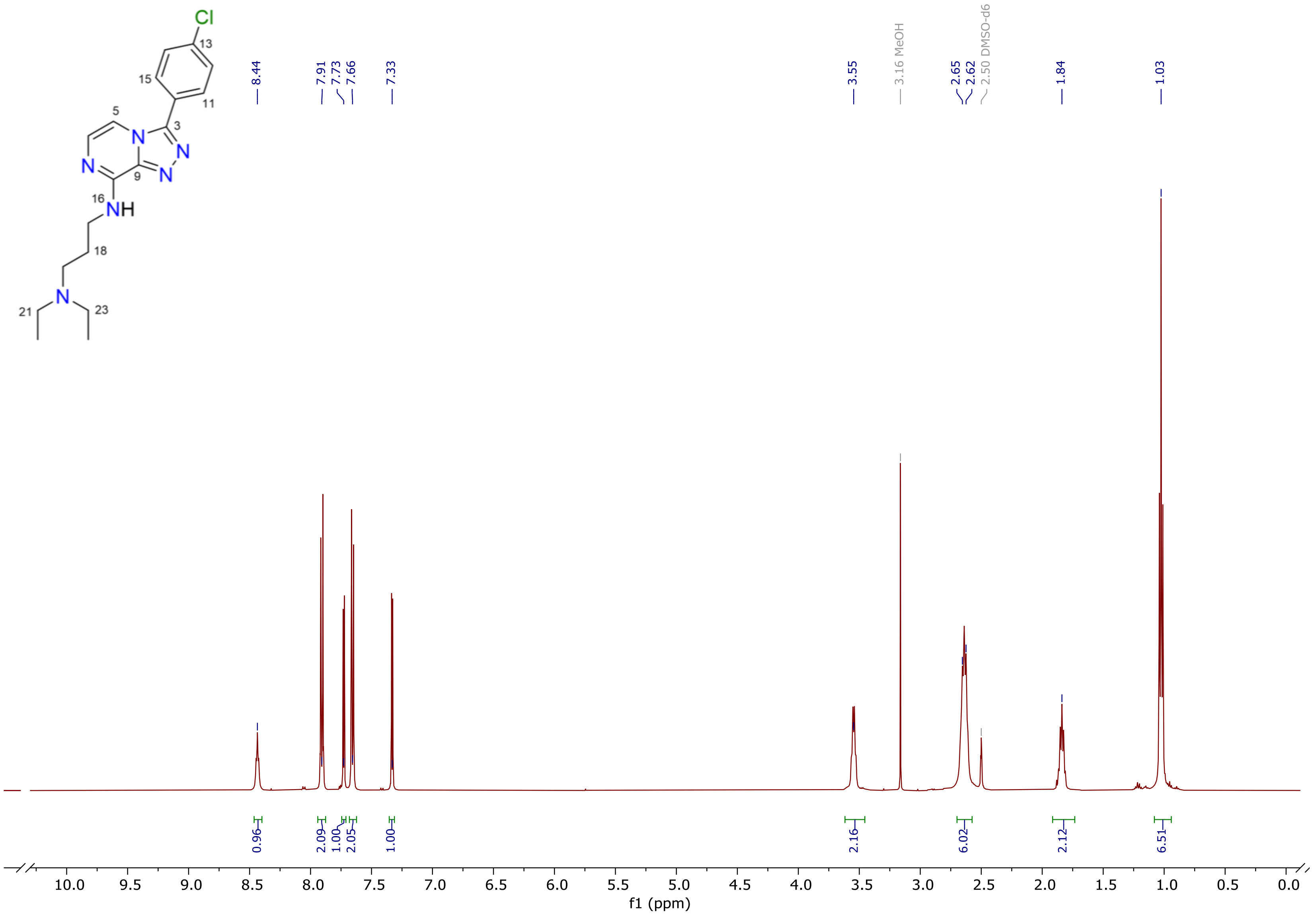

S38: ^{13}C NMR spectrum of compound **13** in $(CD_3)_2SO$

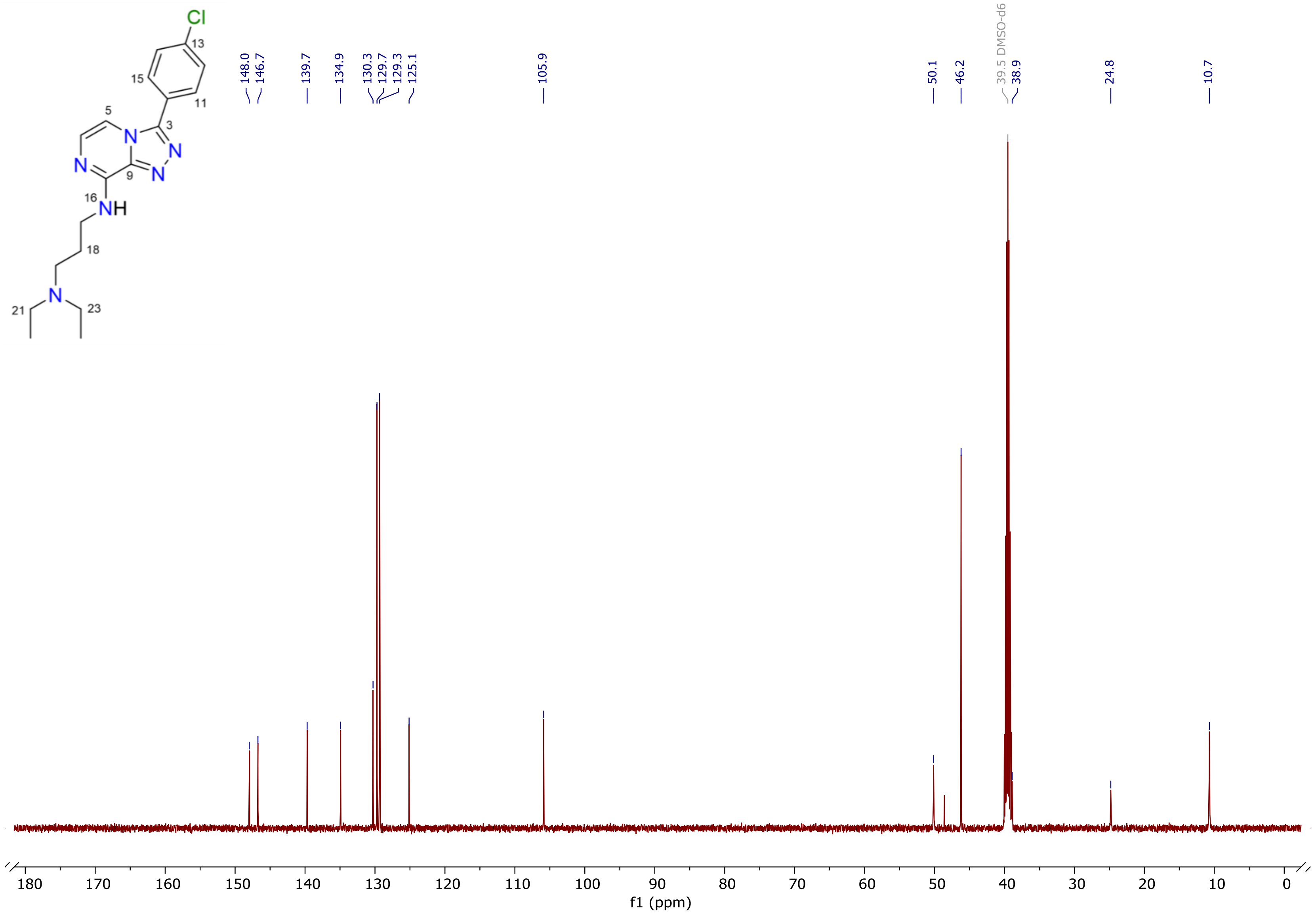

S39: NMR data table for compound **14**[a]

Position	δ_H, mult. (*J* in Hz), int.	δ_C, mult.	COSY	HMBC	ROESY
3		146.3, C			
5	7.81, d (4.7), 1H	107.0, CH	6	3, 6, 9	6, 11, 15
6	7.40, d (4.7), 1H	129.6, CH	5	5, 8	5
8		147.7, C			
9		140.6, C			
10		125.0, C			
11	7.88, m, 1H	130.1, CH_2	12	3, 13, 15	12
12	7.69, m, 1H	129.4, CH_2	11	10, 14	11
13		135.2, C			
14	7.69, m, 1H	129.4, CH_2	15	10, 12	15
15	7.88, m, 1H	130.1, CH_2	14	3, 11, 13	14
16	N.O.				
17	2.73, t (6.4), 2H	38.0, CH_2	18	18	18, 20, 24
18	2.40, t (6.4), 2H	59.6, CH_2	17	17, 20, 24	17, 20, 24
20	2.54, m, 2H	52.9, CH_2	21	18, 21	17, 21
21	4.26, m, 2H	45.8, CH_2	20		20
22	N.O.				
23	4.26, m, 2H	45.8, CH_2	24		24
24	2.54, m, 2H	52.9, CH_2	23	18, 21	18, 23

[a] Recorded in $(CD_3)_2SO$ 500 MHz (^1H NMR) and 125 MHz (^{13}C NMR) at 25 °C.

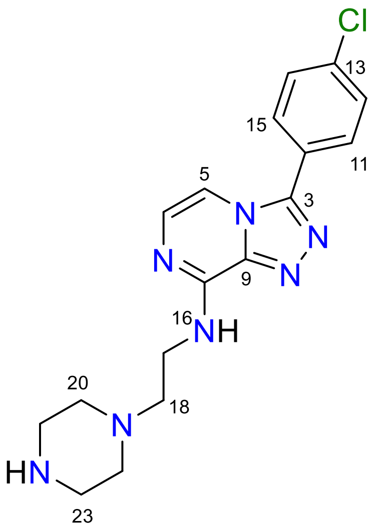

S40: ^{1}H NMR spectrum of compound **14** in $(CD_3)_2SO$

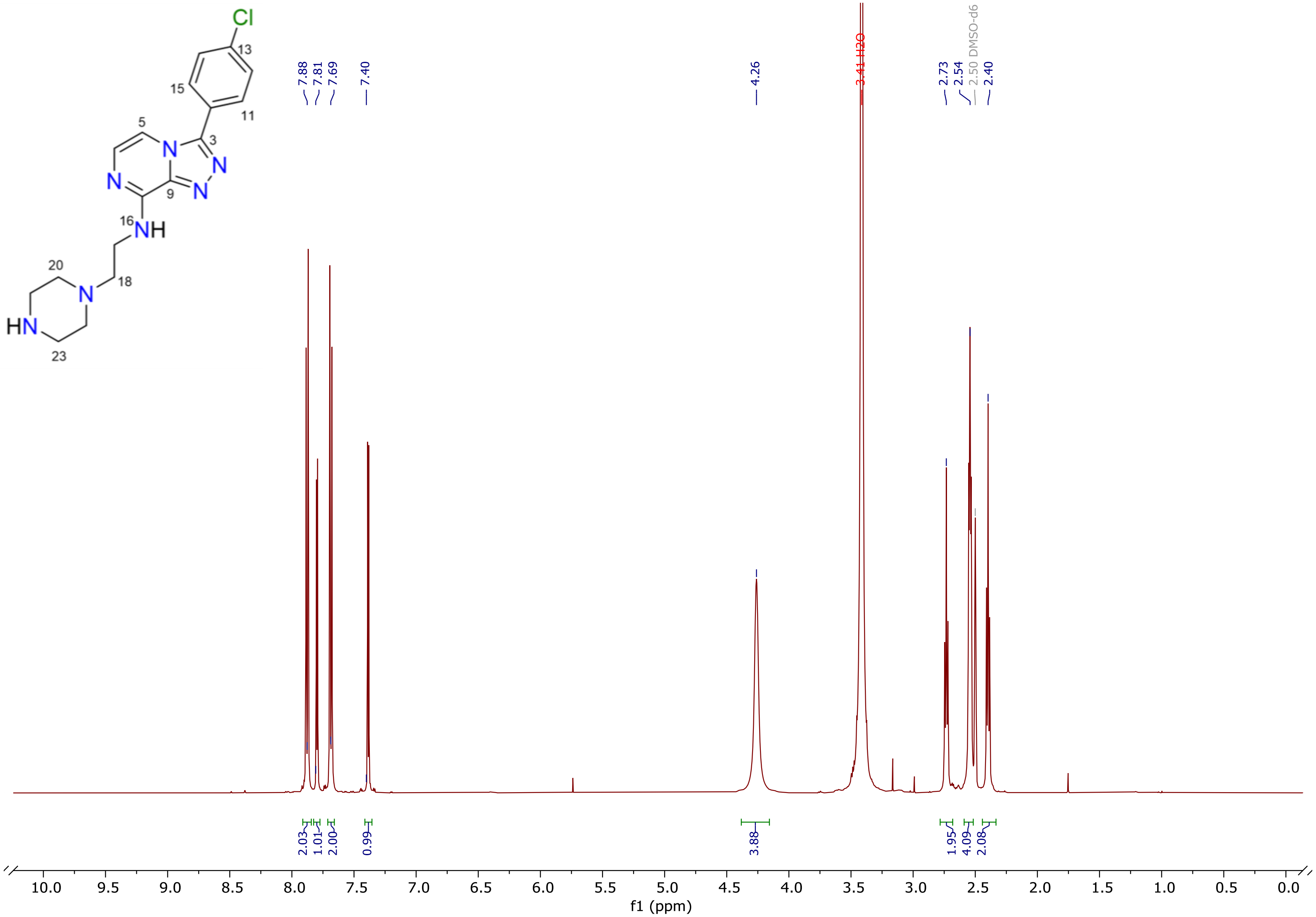

S41: ^{13}C NMR spectrum of compound **14** in $(CD_3)_2SO$

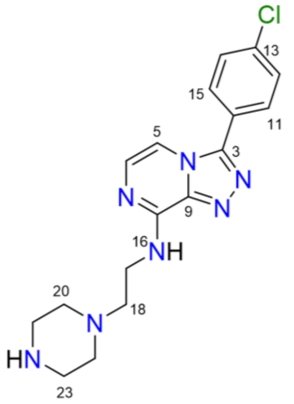

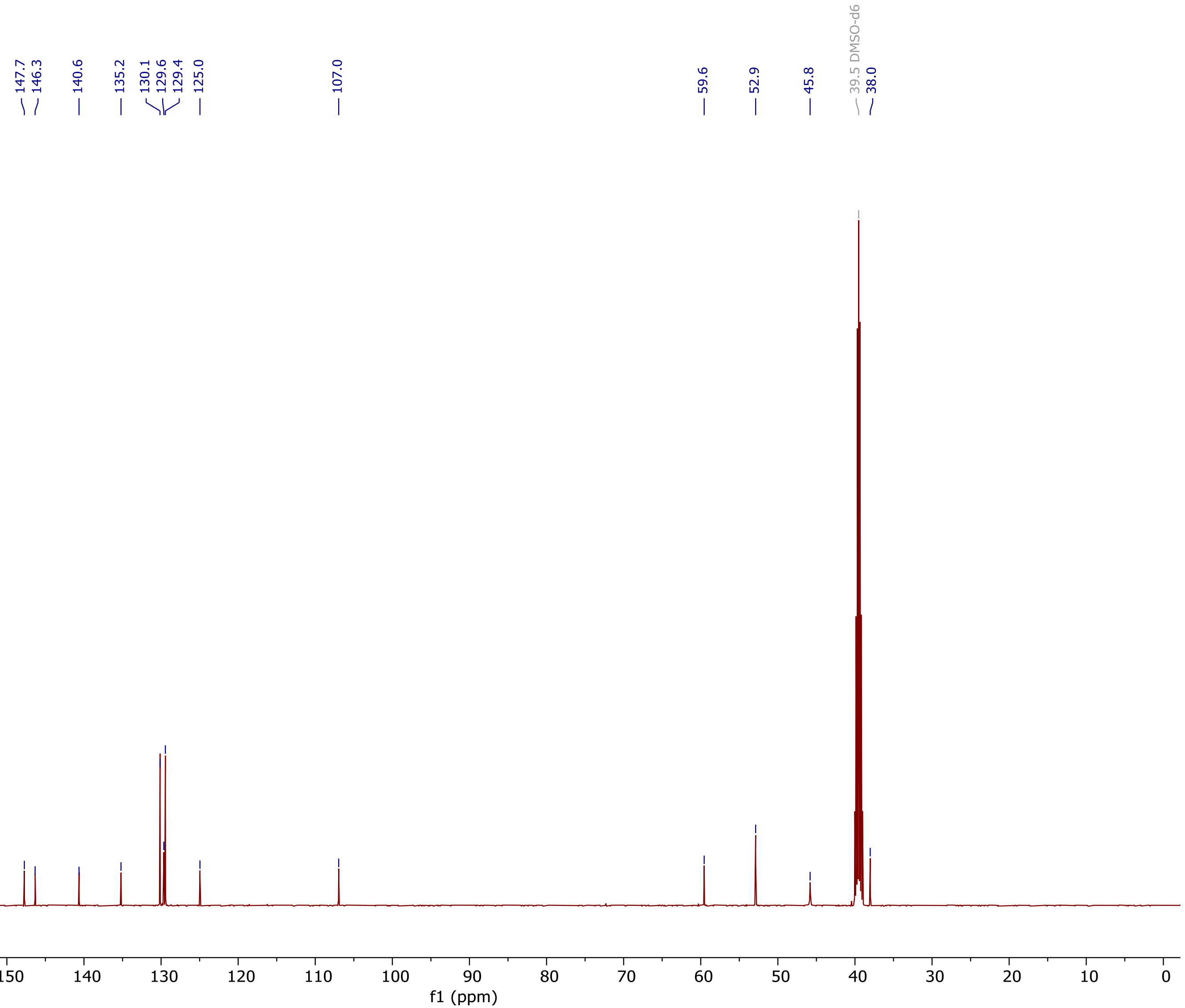

S42: NMR data table for compound **15**[a]

Position	δ_H, mult. (*J* in Hz), int.	δ_C, mult.	COSY	HMBC	ROESY
3		146.8, C			
5	7.71, d (4.8), 1H	105.8, CH	6	3, 6, 9	6
6	7.33, d (4.8), 1H	130.4, CH	5	5, 8	5
8		148.1, C			
9		139.8, C			
10		125.2, C			
11	7.90, m, 1H	129.8, CH	12	3, 13, 15	12
12	7.66, m, 1H	129.4, CH	11	10, 14	11
13		135.0, C			
14	7.66, m, 1H	129.4, CH	15	10, 12	15
15	7.90, m, 1H	129.8, CH	14	3, 11, 13	14
16	8.24, brt (5.7), 1H		17	8	17
17	3.48, m, 2H	39.8, CH_2	16, 18		
18	1.61, m, 2H	26.0, CH_2	17, 19		
19	1.44, m, 2H	27.2, CH_2	18, 20	18, 20	
20	2.94, m, 2H	39.4, CH_2	19, 21		19
21	6.79, t (5.6), 1H		20		20
22		155.7, C			
24		77.4, C			
25	1.35, s, 3H	28.3, CH_3		24	
26	1.35, s, 3H	28.3, CH_3		24	
27	1.35, s, 3H	28.3, CH_3		24	

[a] Recorded in $(CD_3)_2SO$ 500 MHz (^1H NMR) and 125 MHz (^{13}C NMR) at 25 °C.

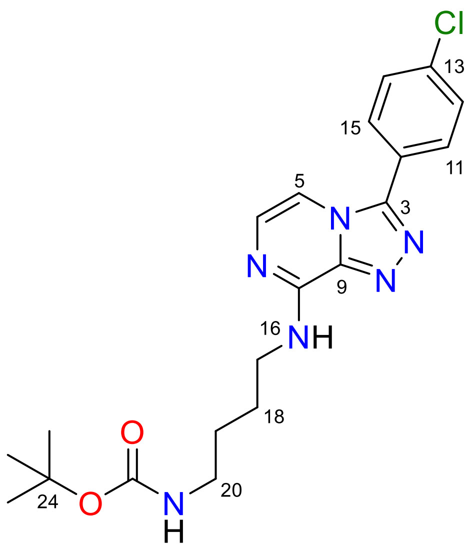

S43: ^{1}H NMR spectrum of compound **15** in $(CD_3)_2SO$

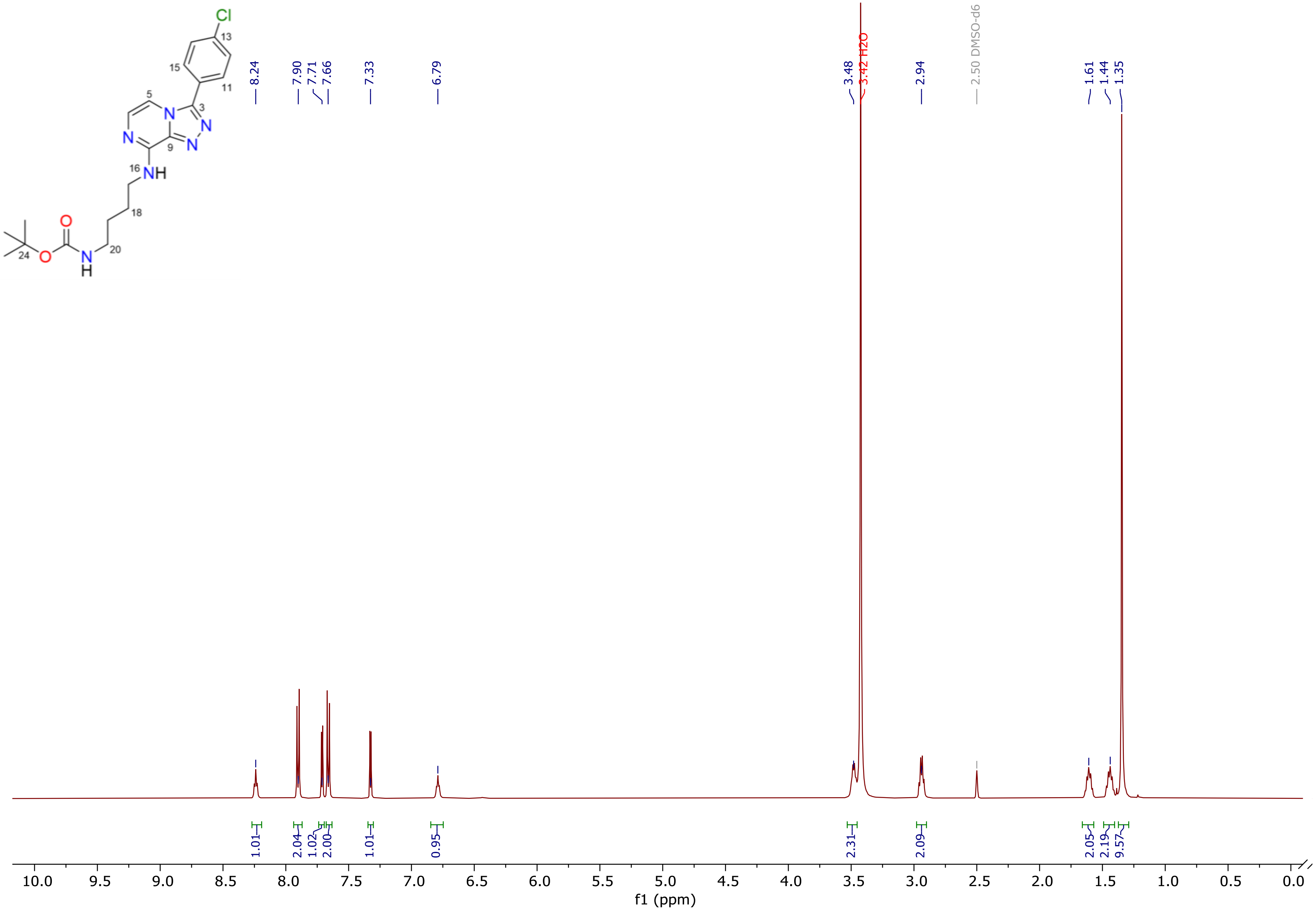

S44: ^{13}C NMR spectrum of compound **15** in $(CD_3)_2SO$

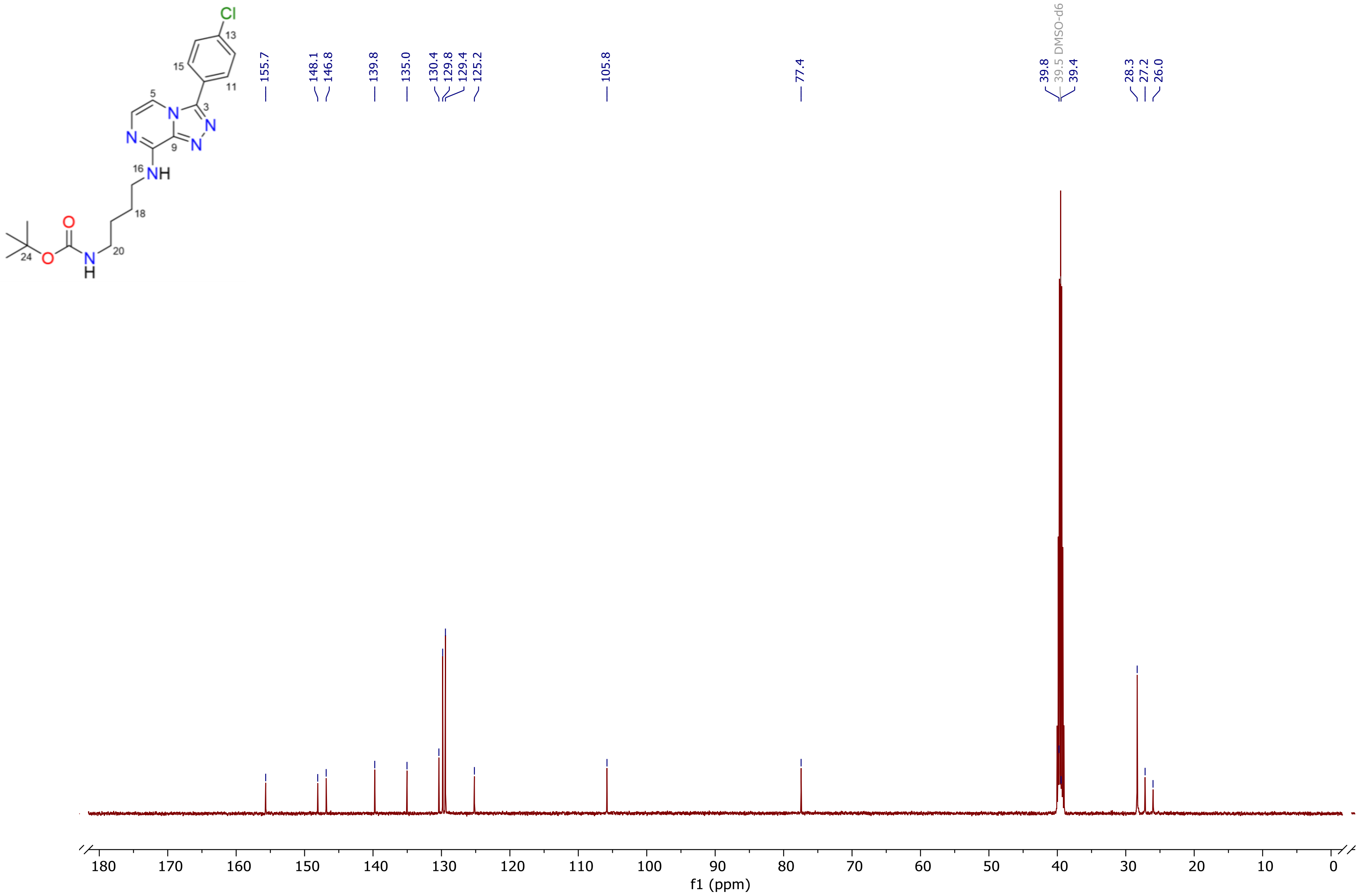

Supplement: File 1 — Complete experimental methods, crystallographic data for 2, 7, 10 and 15, characterisation data and 1D/2D NMR spectra (1H, 13C, COSY, HSQC and HMBC) for 2–15. [file Beilstein_J_Org_Chem-21-1126-s001.pdf]
